# Probing the Mechanism of l‑DOPA 2,3-Dioxygenase Using Synthetic Derivatives of 3,4-Dihydroxyhydrocinnamic Acid

**DOI:** 10.1021/acsomega.5c03691

**Published:** 2025-07-16

**Authors:** Amen Taye Demisew, Jon R. Cohen, Emma G. Gruss, Jennifer D. Bui, Jessica L. Steiner, Gisela Xhafkollari, Ryan N. Marasco, Mark Betonio, David Strzeminski, Sebastian Leyes Porello, Keri L. Colabroy, Larryn W. Peterson

**Affiliations:** † Department of Chemistry, 1143 Muhlenberg College, Allentown, Pennsylvania 18104, United States; ‡ Department of Chemistry, 5414 Rhodes College, Memphis, Tennessee 38112, United States

## Abstract

Extradiol dioxygenase chemistry is well-known in the degradation of natural and man-made sources of aromatic carbon; however, the extent to which biosynthetic dioxygenases like l-DOPA 2,3-dioxygenase can degrade lignin-derived aromatic carbon has not been examined. To understand if l-DOPA 2,3-dioxygenases also demonstrate a capacity or even a proclivity to cleave lignin-derived aromatic carbon into semialdehyde products, the l-DOPA 2,3-dioxygenase reaction was evaluated with derivatives of 3,4-dihydroxyhydrocinnamic acid (DHHCA), also known as hydrocaffeic acid, a catecholic example of lignin-derived carbon. DHHCA analogues, 3-(2-bromo-4,5-dihydroxyphenyl)­propanoic acid (6-bromoDHHCA, **4**), 3-(2-cyano-4,5-dihydroxyphenyl)­propanoic acid (6-cyanoDHHCA, **5**), and 3-(4,5-dihydroxy-2-nitrophenyl)­propanoic acid (6-nitroDHHCA, **6**) were synthesized, their redox potential and p*K*_a_ values were evaluated, and their activity as enzymatic substrates was characterized on two l-DOPA 2,3-dioxygenases from *S. lincolnensis* and *S. hygroscopicus* *jingganensis*. The results indicate that DHHCA, similar in p*K*_a_ values, but smaller and more readily oxidized compared to l-DOPA, is a competent substrate for both enzymes. 6-BromoDHHCA is also readily oxidized, and due to its larger size, is more readily accommodated and cleaved in the more spacious active site of the *S. hygroscopicus* *jingganensis* l-DOPA 2,3-dioxygenase versus the *S. lincolnensis* enzyme. 6-CyanoDHHCA is acidic and difficult to oxidize and its interaction was characterized by significant enzyme inactivation as observed in the presteady state. These results argue for an integrated understanding of substrate size, redox potential, and active site capacity to understand substrate–enzyme compatibility and provide actionable insights to fuel the pursuit of dioxygenase catalysts that robustly cleave 6-X-DHHCA and other synthetically modified lignin-derived catecholic monomers as starting points for functionalized semialdehydes.

## Graphical abstract

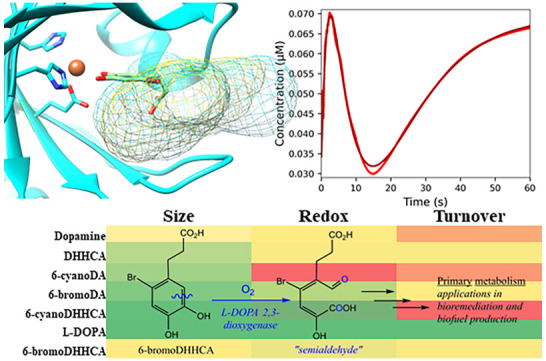

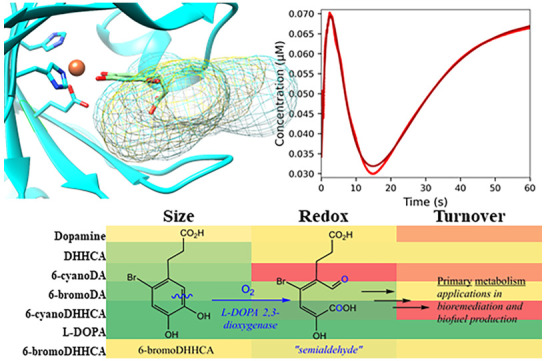

## Introduction

Lignin is the world’s second most abundant polymer, comprising 20–30% of woody plant biomass.^1,2^ It is an untapped source of functionalized catechols, 1,2-dihydroxybenzenes, that could be upcycled into feedstocks and natural products.^3^ Nature already effectively recycles lignin-derived carbon, and this natural rot of woody plant tissue includes a required enzymatic dioxygenation of the aromatic rings.^1^ This dioxygenation chemistry is well-known among bacteria and fungi and comes in two basic varieties: intradiol and extradiol. Intradiol cleavage of the ring by O_2_ occurs between the two hydroxy groups of the diol to yield a muconic acid, and extradiol cleavage adds molecular O_2_ across a double bond that is adjacent to the diol to yield a muconic semialdehyde (Figure 1).^4^

**Figure 1:**
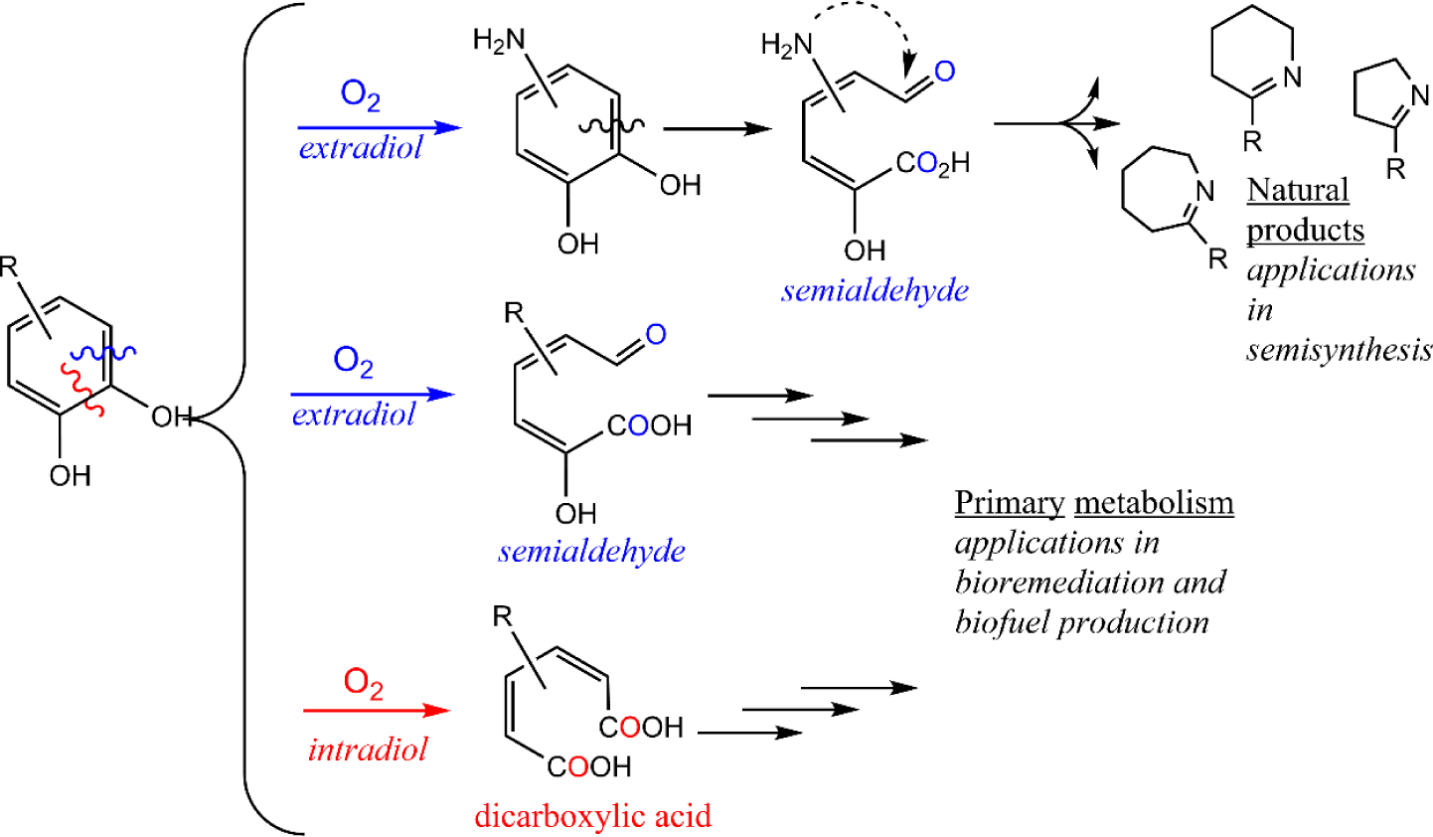
Dioxygenation of 1,2-dihydroxybenzenes is critical for recycling and upcycling of lignin-derived aromatic carbon. Extradiol cleavage (blue) results in a semialdehyde, while intradiol cleavage (red) results in a dicarboxylic acid.

Extradiol dioxygenase (EDX) chemistry has enormous potential for upcycling catecholic carbon because it can accomplish the conversion of 1,2-dihydroxybenzenes into unsaturated semialdehydes in one enzymatic step. For example, lignin-derived aromatic carbon, such as catechol, protocatechuate, and gallate monomers, are degraded to primary metabolites by pathways initiated with the extradiol dioxygenation of the 1,2-dihydroxybenzene core to yield an unsaturated semialdehyde.^5^ Semialdehydes are precursors to olefins, alcohols, and amines.^6,7^ Semialdehydes are also intermediates to dicarboxylic acids, such as muconic and glutaconic acid, which are valuable as monomers in the preparation of nylons, polyesters, and bioplastics.^6,8^ Indeed, semisynthetic routes combining enzymatic and synthetic steps have exploited the cleavage of catechols to access muconic acids for industrial purposes.^9,10^ Lastly, biosynthetic extradiol dioxygenases, such as l-DOPA 2,3-dioxygenase, turn catecholic monomers into natural products via cyclization of the product semialdehyde with an internal amine.^11–15^ This cyclization strategy has also been applied semisynthetically to the semialdehyde products of EDX ring cleavage to yield functionalized pyridines,^16^ which are high-value targets in the synthesis of pharmaceuticals and other specialty chemicals.^17,18^

Strong interest in bacterial extradiol dioxygenase chemistry for industrial purposes is also fueled by the flexibility and adaptability of extradiol dioxygenases to a variety of substrates. The degradation of polychlorinated biphenyls (PCBs) and petroleum hydrocarbons contaminating water and soil is driven by bacterial extradiol dioxygenases, and many of these same bacterial species can robustly cleave lignin-derived metabolites as well.^19–24^ Perhaps this is not surprising; it is plausible to propose that lignin-derived aromatic carbon was the *first* source of aromatic carbon that ancestral extradiol dioxygenases evolved to cleave. For example, bacteria capable of degrading PCBs are often found in close natural association with tree roots in PCB-contaminated sites.^25,26^ Additionally, in lignin-degrading *Burkholderia* sp., genes for lignin-fragment degradation have been found in pathways that metabolize aromatic xenobiotics, including petroleum-derived aromatic hydrocarbons.^27^ These observations imply that the extradiol dioxygenase enzymes responsible for degrading PCBs and petroleum-derived aromatic hydrocarbons are also capable of degrading lignin metabolites, as well. It is therefore plausible to consider that other extradiol dioxygenases found in peripheral or secondary pathways may also have the capacity to cleave lignin-derived aromatic carbon.

l-DOPA 2,3-dioxygenase is a small, evolutionarily distinct extradiol dioxygenase of the vicinal oxygen-chelate (VOC) superfamily. While distantly related to well-studied VOC extradiol dioxygenase enzymes,^28–30^ l-DOPA 2,3-dioxygenase is not primarily a degradation enzyme; rather it is found as part of biosynthetic pathways. l-DOPA 2,3-dioxygenase was first discovered as part of a biosynthetic gene cluster to the *S. lincolnensis*-derived antibiotic lincomycin.^31,32^ It was subsequently identified in five additional natural products,^33–37^ and most recently, within cryptic biosynthetic pathways in *S. sclerotialus* ^38^ and *S. hygroscopicus* *jingganensis*.^39^ In these systems, l-DOPA 2,3-dioxygenase cleaves l-3,4-dihydroxyphenylalanine (l-DOPA) to a muconic semialdehyde, which spontaneously cyclizes onto the amino-containing side chain to yield a pyrroline ring. This transformation appears as part of a biosynthetic mini-pathway to the synthon 3-vinyl-2,3-pyrroline-5-carboxylic acid (VPCA).^38^ In lincomycin and related natural products, this synthon is elaborated and embedded within the final product structure (Figure S1). This biosynthetic role makes l-DOPA 2,3-dioxygenases exceptions in the long history of extradiol dioxygenase chemistry in the degradation of natural and man-made sources of aromatic carbon. While l-DOPA 2,3-dioxygenase is already a promising catalyst for the cleavage of diverse 3,4-dihydroxyphenethylamine scaffolds to yield cyclized pyrrolines, its capacity for generating linear semialdehydes from lignin-derived catechols has not been examined.^37,38^ In order to understand if l-DOPA 2,3-dioxgenases possess the capacity or even a proclivity to cleave lignin-derived aromatic carbon to yield linear semialdehyde products, we chose the scaffold of 3,4-dihydroxyhydrocinnamic acid (DHHCA), also known as hydrocaffeic acid, a catecholic example of lignin-derived carbon, as the substrate of interest.^40,41^ DHHCA is similar in structure to the preferred substrate of l-DOPA 2,3-dioxygenase, l-DOPA, but it lacks a side chain amine, which makes cyclization to a pyrroline impossible. DHHCA is also amenable to synthetic functionalization of the aromatic ring with electron-withdrawing substituents of different sizes, providing us a platform to study the perturbations of these substituents on the enzymatic mechanism of l-DOPA 2,3-dioxygenase.

Here we report the synthesis of DHHCA analogues with nitro, bromo, and cyano substituents to probe the activity and mechanism of l-DOPA 2,3-dioxygenase enzymes (Figure 2). 3-(2-Bromo-4,5-dihydroxyphenyl) propanoic acid (6-bromoDHHCA, **4**), 3-(2-cyano-4,5-dihydroxyphenyl) propanoic acid (6-cyanoDHHCA, **5**), and 3-(4,5-dihydroxy-2-nitrophenyl) propanoic acid (6-nitroDHHCA, **6**) were also evaluated for their physical properties and activity as enzymatic substrates of two l-DOPA 2,3-dioxygenases from *S. lincolnensis* and *S. hygroscopicus* *jingganensis.* Our results indicate that DHHCA is a reasonable substrate for both enzymes, while 6-bromoDHHCA can be accommodated and processed as a substrate in the more spacious active site of the *S. hygroscopicus* *jingganensis* l-DOPA 2,3-dioxygenase. 6-CyanoDHHCA is characterized by significant inactivation of the enzyme as observed in the presteady state. Taken together, these results argue for an integrated understanding of substrate size, redox potential, and active site capacity to understand substrate–enzyme compatibility and provide actionable insights to fuel the pursuit of dioxygenase catalysts that robustly cleave 6-X-DHHCA and other synthetically modified, lignin-derived catecholic monomers as starting points for functionalized semialdehydes.

**Figure 2:**
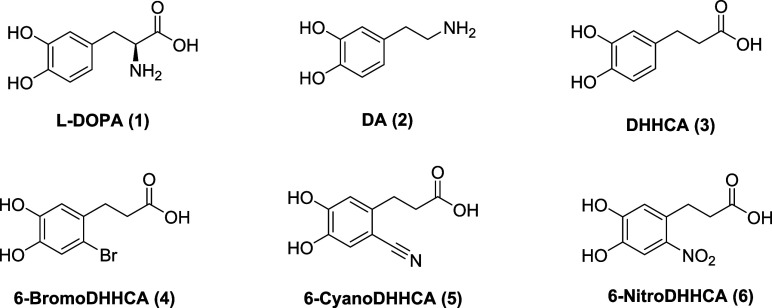
Structures of l-DOPA (1), dopamine (DA, **2**), 3,4-dihydroxyhydrocinnamic acid (DHHCA, **3**), and derivatives investigated in this study: 6-bromoDHHCA (**4**), 6-cyanoDHHCA (5), and 6-nitroDHHCA (**6**).

## Materials and Methods

### General Methods for Small Molecule Synthesis and Analysis

All anhydrous solvents were commercially obtained and stored in Sure-Seal bottles under an argon atmosphere, unless otherwise indicated. All other reagents and solvents were purchased as the highest grade available from Acros or Sigma-Aldrich and were used without further purification. Moisture-sensitive reactions were handled with anhydrous solvents with an argon atmosphere in a conventional flask. The transfer of reagents and solvents was performed using disposable and glass syringes that were obtained commercially. When necessary, purification of the compounds, as determined by TLC, was completed with column chromatography using silica gel (Silicycle 55–65 Å). Analysis of the product after each synthesis step was performed with (^1^H) proton and (^13^C) carbon NMR spectra using a Varian 400 MHz or a Bruker Avance 400 MHz spectrometer. Chemical shifts (δ) are reported in parts per million (ppm) and referenced to ^1^H (CHCl_3_ at 7.26, CH_3_OH at 3.31) and ^13^C (CDCl_3_ at 77.16, CD_3_OD at 49.15). Coupling constants (*J*) are reported in Hz throughout. The NMR spectra are included in Figures S3–S20. High-resolution mass spectra (HRMS) were obtained at the Ole Miss Mass Spectrometry Facility using a QTOF spectrometer utilizing nanospray ionization in negative ion mode. The purity of the final compounds was determined with HPLC analysis: Shimadzu LC-6AD pumps, SPD-M20A PDA, CBM-20A communication, and a Thermo Scientific column (Hypersil GOLD, 250 × 4.6 mm, particle size = 5 μm). The HPLC chromatograms for final compounds **4–6** are included in Figures S21–S23.

### Synthesis of Methyl 3-(3,4-Dimethoxyphenyl)­Propanoate (7)

3-(3,4-Dimethoxyphenyl)­propanoic acid (1.00 g, 4.76 mmol) was dissolved in CH_3_OH (12.5 mL) under an Ar atmosphere at rt. SOCl_2_ (0.69 mL, 9.51 mmol) was added dropwise, and the resulting solution was stirred for 23 h at rt. The solution was poured over ice water (30 mL), and the organic layer was extracted with EtOAc (4 × 15 mL), dried with Na_2_SO_4_, and filtered through cotton. The resulting solution was concentrated under reduced pressure and high vacuum evaporation. The resulting oil was then allowed to crystallize in the freezer for 2 h, yielding methyl 3-(3,4-dimethoxyphenyl)­propanoate **(7)** as a white crystalline solid with a yellow tint (930 mg, 87.2%). ^**1**^ **H NMR** (400 MHz, CDCl_3_) δ 6.83–6.67 (m, 3H), 3.86 (2x s, 6H), 3.67 (s, 3H), 2.90 (t, *J* = 7.8 Hz, 2H), 2.61 (t, *J* = 7.8 Hz, 2H). ^**13**^ **C NMR** (101 MHz, CDCl_3_) δ 173.41, 148.92, 147.56, 133.15, 120.09, 111.63, 111.29, 55.91, 55.82, 51.63, 36.01, 30.61.

### Synthesis of Methyl 3-(2-Bromo-4,5-Dimethoxyphenyl) Propanoate (8)

Methyl 3-(3,4-dimethoxyphenyl) propanoate **(7)** (330 mg, 1.57 mmol) was dissolved in AcOH (2.50 mL) under an Ar atmosphere at rt. Br_2_ (0.1 mL, 1.94 mmol) and AcOH (0.70 mL) were combined under an Ar atmosphere before being added to the solution dropwise. The resulting solution was allowed to stir for 2.5 h at rt. The reaction solution was dissolved with CH_2_Cl_2_ (10 mL), washed with sodium thiosulfate (1 M, 30 mL), and extracted with CH_2_Cl_2_ (3 × 10 mL). The resulting solution was washed with saturated NaHCO_3_ solution (4 × 20 mL) and then washed again with saturated brine solution (3 × 20 mL). The organic layer was then dried with anhydrous Na_2_SO_4_ and concentrated under pressure to yield the crude product. The resulting product was then purified using column chromatography (1.2:1 Hex:EtOAc) and concentrated under pressure to yield methyl 3-(2-bromo-4,5-dimethoxyphenyl) propanoate **(8)** as a clear oil (240 mg, 50.4%). **R**_**f**_ (1.2:1 Hex:EtOAc): 0.79. ^**1**^ **H NMR** (400 MHz, CDCl_3_) δ 6.99 (s, 1H), 6.77 (s, 1H), 3.85 (2x s, 6H), 3.68 (s, 4H), 2.99 (t, *J* = 7.8 Hz, 2H), 2.62 (t, *J* = 7.8 Hz, 2H). ^**13**^ **C NMR** (101 MHz, CDCl_3_) δ 173.07, 148.35, 148.16, 131.65, 115.57, 113.92, 113.14, 56.12, 56.01, 51.62, 34.24, 31.13.

### Synthesis of 3-(2-Bromo-4,5-Dimethoxyphenyl) Propanoic Acid (9)

Methyl 3-(2-bromo-4,5-dimethoxyphenyl) propanoate (**8)** (180 mg, 0.501 mmol) was dissolved in a minimal amount of CH_3_OH (1.5 mL). KOH (1.07 M, 3.5 mL, 3.75 mmol) was added dropwise and stirred at room temperature for 2.5 h. The solution was diluted with EtOAc (5 mL) and acidified with 1 M HCl (4 mL) until a pH of 2. The solution was extracted with EtOAc (3 × 5 mL). The solution was washed with brine (10 mL) and dried with anhydrous Na_2_SO_4_. The resulting solution was concentrated under reduced pressure and high vacuum evaporation to afford 3-(2-bromo-4,5-dimethoxyphenyl) propanoic acid **(9)** as a white powder solid (146 mg, 65.1%). ^**1**^ **H NMR** (400 MHz, CDCl_3_) δ 6.99 (s, 1H), 6.77 (s, 1H), 3.83 (2x s, 6H), 2.98 (t, *J* = 6.8 Hz, 2H), 2.67 (t, *J* = 7.7 Hz, 2H). ^**13**^ **C NMR** (101 MHz, CDCl_3_) δ: 179.18, 148.35, 148.23, 131.27, 115.62, 113.97, 113.19, 56.13, 56.01, 34.22, 30.82.

### Synthesis of 3-(2-Bromo-4,5-Dihydroxyphenyl) Propanoic Acid (4)

3-(2-Bromo-4,5-dihydroxyphenyl) propanoic acid **(9)** (131 mg, 0.453 mmol) was dissolved in anhydrous CH_2_Cl_2_ and cooled to −78 °C under an Ar atmosphere. BBr_3_ (1 M in CH_2_Cl_2_, 1.40 mL, 1.40 mmol) was then added dropwise, and the reaction mixture was stirred at the cooled temperature for 15 min. The reaction solution was then brought to rt and stirred for 2.5 h. The reaction was quenched, while still under an Ar atmosphere, with H_2_O:CH_3_CN (1:3, 1 mL). The solvent was removed under reduced pressure, still under an Ar atmosphere, and then the product was extracted with Et_2_O (12 mL). The solvent was removed under reduced pressure, yielding 3-(2-bromo-4,5-dihydroxyphenyl) propanoic acid **(4)** as a tannish-white solid (120 mg, quant.). ^**1**^ **H NMR** (400 MHz, CD_3_OD) δ 6.91 (s, 1H), 6.72 (s, 1H), 2.85 (t, *J* = 7.8 Hz, 2H), 2.52 (t, *J* = 7.8 Hz, 2H). ^**13**^ **C NMR** (101 MHz, CD_3_OD) δ: 175.13, 144.80, 144.63, 130.46, 118.71, 116.48, 111.67, 34.02, 30.40. **HPLC:** 95% at 275 nm (t_R_: 1.694 min). **HRMS** (ESI): *m*/*z* calc for C_9_H_9_BrO_4_ [M-H]^−^ 258.9651 was found to be 258.9732.

### Synthesis of Methyl 3-(2-Iodo-4,5-Dimethoxyphenyl) Propanoate (10)

Iodine monochloride (0.22 M in AcOH, 19.4 mL, 4.28 mmol) was added dropwise, and methyl 3-(3,4-dimethoxyphenyl) propanoate (**9**, 296 mg, 1.32 mmol) was dissolved under an Ar atmosphere. The solution was stirred overnight at rt for 18 h. The reaction mixture was diluted with CH_2_Cl_2_ (10 mL), washed with sodium thiosulfate (1 M, 20 mL), and extracted with CH_2_Cl_2_ (3 × 15 mL). The solution was then washed with a saturated solution of NaHCO_3_ (10 mL) and extracted with CH_2_Cl_2_ (10 mL). The solution was washed a third time with brine (20 mL) and extracted with CH_2_Cl_2_ (10 mL). The solution was dried with Na_2_SO_4_ and was concentrated *in vacuo* to yield the crude product. The crude product was purified using column chromatography run in 1:1 hexanes:EtOAc to yield methyl 3-(2-iodo-4,5-dimethoxyphenyl) propanoate (**10**) as a white-yellow solid (360 mg, 77.9%). **R**_**f**_ (1:1 Hex:EtOAc): 0.75. ^**1**^ **H NMR** (400 MHz, CDCl_3_) δ 7.15 (s, 1H), 6.73 (s, 1H), 3.78 (2x s, 6H), 3.63 (s, 3H), 2.93 (t, *J* = 7.2 Hz, 2H), 2.55 (t, *J* = 7.2 Hz, 2H). ^**13**^ **C NMR** (101 MHz, CDCl_3_) δ 172.89, 149.30, 148.04, 135.37, 121.65, 112.35, 87.66, 56.10, 55.87, 51.63, 35.52, 34.52.

### Synthesis of Methyl 3-(2-Cyano-4,5-Dimethoxyphenyl) Propanoate (11)

Methyl 3-(2-iodo-4,5-dimethoxyphenyl) propanoate (**10,** 559 mg, 1.60 mmol) and CuCN (157 mg, 1.76 mmol) were dissolved in anhydrous DMF (2.40 mL). The solution was stirred for 6 h at 130 °C under an Ar atmosphere. The reaction solution was diluted with EtOAc (5 mL) and filtered through cotton (2×), and diluted again with EtOAc (5 mL), all over ice. The solution was then hydrolyzed with NH_4_OH (10% v/v, 10 mL) and the crude product was extracted with EtOAc (2 × 10 mL). The resulting solution was dried with MgSO_4_, and the crude product was concentrated *in vacuo*. The crude product was purified via column chromatography in a 1:1.5 solution of hexanes:EtOAc to yield methyl 3-(2-iodo-4,5-dimethoxyphenyl)­propanoate **(11)** as a white crystalline solid (270 mg, 67.9%). **R**_**f**_ (1:1.5 Hex:EtOAc): 0.77. ^**1**^ **H NMR** (400 MHz, CDCl_3_) δ 7.01 (s, 1H), 6.81 (s, 1H), 3.89 (2x s, 6H), 3.67 (s, 3H), 3.10 (t, *J* = 7.5 Hz, 2H), 2.69 (t, *J* = 7.5 Hz, 2H). ^**13**^ **C NMR** (101 MHz, CDCl_3_) δ 172.70, 152.75, 147.74, 138.92, 118.30, 114.26, 112.27, 103.35, 77.36, 56.26, 56.18, 51.90, 35.01, 29.78, 29.41.

### Synthesis of 3-(2-Cyano-4,5-Dimethoxyphenyl) Propanoic Acid (12)

Methyl 3-(2-cyano-4,5-dimethoxyphenyl) propanoate (**11**, 125 mg, 0.501 mmol) was dissolved in a minimal amount of CH_3_OH (1.1 mL). NaOH (1M, 2.50 mL, 2.50 mmol) was added dropwise and stirred at rt for 2.5 h. The solution was acidified with 2 M HCl until a pH of 4 was reached. The solution was extracted with EtOAc (3 × 5 mL). The solution was washed with brine (10 mL), extracted with EtOAc (10 mL), and dried with Na_2_SO_4_. The solution was concentrated *in vacuo* to yield 3-(2-cyano-4,5-dimethoxyphenyl) propanoic acid (**12**) as a white powdery solid (110 mg, 93.2%). ^**1**^ **H NMR** (400 MHz, CDCl_3_) δ 7.03 (s, 1H), 6.83 (s, 1H), 3.92 (s, 3H), 3.88 (s, 3H), 3.12 (t, *J* = 7.5 Hz, 2H), 2.75 (t, *J* = 7.5 Hz, 2H). ^**13**^ **C NMR** (101 MHz, CDCl_3_) δ 177.48, 152.90, 147.94, 138.59, 118.26, 114.47, 112.40, 103.49, 56.35, 56.25, 34.76, 29.14.

### Synthesis of 3-(2-Cyano-4,5-Dihydroxyphenyl) Propanoic Acid (5)

3-(2-Cyano-4,5-dimethoxyphenyl) propanoic acid (**12**, 284 mg, 1.14 mmol) was dissolved in anhydrous CH_2_Cl_2_ (8.5 mL) under an Ar atmosphere. At −78 °C, BBr_3_ (1 M in CH_2_Cl_2_, 4.50 mL, 4.50 mmol) was added dropwise, and the mixture was stirred for 15 min. The reaction was then warmed to rt and the reaction continued to stir for 2.5 h. The reaction was quenched with 1:3 water:CH_3_CN (1 mL) and stirred for 25 min at rt. The solution was concentrated *in vacuo* and extracted with Et_2_O (3 × 5 mL). The solvent was concentrated *in vacuo* to yield 3-(2-cyano-4,5-dihydroxyphenyl) propanoic acid (**5**) as a pale orange solid (215 mg, 91.0%). ^**1**^ **H NMR** (400 MHz, CD_3_OD) δ 6.98 (s, 1H), 6.79 (s, 1H), 2.96 (t, *J* = 7.3 Hz, 2H), 2.60 (t, *J* = 7.3 Hz, 2H). ^**13**^ **C NMR** (101 MHz, CD_3_OD) δ 174.43, 151.77, 145.60, 138.56, 119.54, 117.36, 102.61, 35.79, 29.95. **HPLC**: 95% at 253 nm (t_R_: 1.700 min). **HRMS** (ESI): *m*/*z* calc for C_10_H_9_NO_4_ [M-H]^−^ 206.0459 was found to be 206.0414.

### Synthesis of 3-(4,5-Dihydroxy-2-Nitrophenyl) Propanoic Acid (6)

3,4-Dihydroxyhydrocinnamic acid (**3**, DHHCA, 253 mg, 1.38 mmol) and NaNO_2_ (476 mg, 6.90 mmol) were dissolved in H_2_O (3 mL), stirred, and cooled to 0 °C in an ice bath for 5 min. H_2_SO_4_ (5%, 1.8 mL, 33.6 mmol) was added dropwise to the reaction and continued to stir for 15 min in an ice bath. The product was extracted with EtOAc (3 × 10 mL) and dried with Na_2_SO_4_. The product was concentrated *in vacuo*, yielding 3-(4,5-dihydroxy-2-nitrophenyl) propanoic acid (**6**) as a light orange solid (159 mg, 50.5%). ^**1**^ **H NMR** (400 MHz, CD_3_OD) δ 7.56 (s, 1H), 6.80 (s, 1H), 3.13 (t, *J* = 7.2 Hz, 2H), 2.64 (t, *J* = 7.1 Hz, 2H). ^**13**^ **C NMR** (101 MHz, CD_3_OD) δ 176.40, 152.36, 145.23, 141.43, 131.01, 118.66, 113.32, 35.67, 29.80. **HPLC**: 95% at 352 nm (t_R_: 2.419 min). **HRMS** (ESI): *m*/*z* calc for C_9_H_9_NO_6_ [M-H]^−^ 226.0357 was found to be 226.0318.

### Determination of p*K*_a_ Values

A buffer system utilizing five different buffers was prepared to cover the pH range of 3.0 to 12.50. A Thermo Scientific Orion Star A211 pH meter was used to measure and determine the pH of each buffer. The pH meter was calibrated using three- and four-point calibration with pH values of 4.00, 7.01, and 10.01 and 4.00, 7.01, 10.01, and 12.00, respectively.

The buffer systems were prepared as previously described^42^ and included: NaOAc/HOAc buffers (*C* = 0.1 M; *I* = 0.1 M) were used to cover the pH range of 3.0 to 5.50. KH_2_PO_4_/K_2_HPO_4_ buffers (*C* = 0.05 M; *I* = 0.1 M) were used to cover the pH range of 6.00 to 8.00. CHES buffers (*C* = 0.05 M; *I* = 0.1 M) were used to cover the pH range of 8.50 to 10.50. Na_2_HPO_4_/Na_3_PO_4_ buffers (*C* = 0.05 M; *I* = 0.1 M) were used to cover the pH range of 11.00 to 12.00. KCl buffers (*C* = 0.1 M; *I* = 0.1 M) were used to cover the pH range of 12.00 to 12.50.

The absorbance spectra of the compounds were determined at each pH as previously described.^42^ Briefly, a stock solution was prepared for each compound by weighing 2 mg of the compound using a high-precision analytical balance. Each compound was then dissolved in DMSO at a concentration of 10 mM. A UV-transparent 96-well microplate (Corning clear acrylic 96-well UV plate with a UV-transparent flat bottom) was loaded with 196 μL of buffer solution and 4 μL of the catechol stock in each well to get a final concentration of 200 μM. DMSO (4 μL) was added to 196 μL of the appropriate buffer to produce a blank solution for each buffer.

The 96-well plate was loaded into the microplate reader and incubated at 30 °C and shaken for 10 min before the reading. Absorbance readings were completed by using a Synergy HTX 15032517 microplate reader. Each well was read at wavelengths from 210 to 600 nm with 2-nm increments for each scan over a duration of 60 min.

The data were analyzed as previously described.^42^ Raw UV-spectra scans were exported to the Excel program. They were first processed by subtracting the reading of the blank solutions from the reading of each compound at the corresponding wavelengths. Then, these data were normalized where absorbance at 600 nm was zero by subtracting the absorbance at 600 nm from the absorbance at all other wavelengths for each corresponding buffer. Finally, the spectral difference between the pH 3.0 buffer and the spectra at all other pH values was plotted. Additionally, the spectral difference between the pH 4.0 buffer and the spectra at all other higher pH values was plotted. The wavelengths for the maximum positive and negative absorbance were determined by using the graphs of the spectral difference plots, both for the pH 3.0 and pH 4.0 spectral difference plots. The absolute maximum positive and total absorbance differences at these chosen wavelengths were plotted against the pH of each buffer. The data were imported into the Origin 6.0 program, and the p*K*_a_ values were determined with a nonlinear regression using eq 1:

1 $$\mathrm{Absorbance}\;\mathrm{total}=\frac{\varepsilon_{HA}-\varepsilon_{A}-\left[10^{\left(\mathrm{pH}-pKa\right)}\right]}{1+10^{\left(\mathrm{pH}-pKa\right)}}\times \left[S_{t}\right]$$

ε_HA_ is the extinction coefficients of the acid form of the compound, and ε_A_^–^ is the extinction coefficient of the base form of the compound (i.e., the minima and maxima of the absorbance difference curve, respectively), and [*S*_t_] is the total compound concentration. The raw UV spectra, the normalized spectra, and the Origin plots can be found in Figures S24–S39.

### Cyclic Voltammetry

Reduction and oxidation potentials were experimentally performed by cyclic voltammetry (CV) at biological pH 7.4, as previously described.^42^ All samples were run on a CH Instruments 600E Potentiostat in a one-compartment cell equipped with a three-electrode system arrangement. The three-electrode system included a saturated calomel electrode as the reference electrode, a platinum wire as the auxiliary electrode, and a glassy carbon electrode (GCE, *d* = 2 mm) as the working electrode. The GCE was polished between each sample scan with an aqueous slurry of alumina powder on a microcloth pad. The electrodes were rinsed with distilled water prior to the next scan. All scans were conducted under nitrogen for oxygen displacement and at room temperature (rt, 25 ± 1 °C). All samples were prepared as close to the scan as possible (approximately 2 min each). Each sample (5 mL) contained 0.1 mM catecholic substrate and 1 μM DMSO in 0.1 M phosphate buffer at a pH of 7.4 or 6.0. To facilitate comparison among catecholic substrates, all synthetic and commercially available catechols were prepared, scanned as a single experimental set, and normalized to the *E*_1/2_ (mV) of dopamine. The cyclic voltammograms for the compounds at both pH 6.0 and 7.4 are available in Figures S40–S51.

### Overexpression and Purification of l-DOPA 2,3-Dioxygenases

The standard molecular biology procedures for bacterial growth, plasmid preparation, and transformation of competent cells were performed as described by Sambrook et al.^43^ The plasmid pET16B1, which contains l-DOPA 2,3-dioxygenase from *Streptomyces lincolnensis* (GenBank ID: X79146.1, NCBI protein: CAA55747, LmbB1) was a generous gift from W. Piepersberg.^32^ The pET28a-derived plasmid pShjingDDO containing l-DOPA 2,3-dioxygenase from *Streptomyces hygroscopicus* *jingganensis* (*Shj*DDO gene: SHJG_8633, NCBI: AEY93898.1, UniProt: H2JL11) was generated as previously described.^39^ Both plasmids were transformed into *Escherichia coli* BL21 (DE3) cells (Thermo Fisher).

Transformed *E. coli* cells were used to inoculate 100 mL of Luria–Bertani medium containing 50 μg/mL kanamycin (for *Shj*DDO) or 200 μg/mL ampicillin (for LmbB1) and incubated at 37 °C with agitation (220–225 rpm) overnight. The resulting 100 mL starter cultures were used to inoculate 900 mL of Luria–Bertani medium with a final concentration of 50 μg/mL kanamycin (for *Shj*DDO) or 200 μg/mL ampicillin (for LmbB1). Once the cultures reached an OD_600_ of ∼0.6–0.8, the temperature was lowered to 18 °C, and isopropyl-β-thiogalactoside was added to a final concentration of 0.1 mM. The cultures were then incubated for an additional 18–24 h. Cells were harvested by centrifugation at 5000× *g* for 20 min at 4 °C and resuspended in a lysis buffer [50 mM phosphate, 300 mM NaCl, 10 mM imidazole, 10% glycerol, 0.1% Triton X-100] at a ratio of 5 mL per gram of cell pellet. The resuspension was kept chilled at 4 °C and lysed by sonication (Hielscher), followed by bead milling with 0.1-mm glass beads according to the manufacturer’s instructions (BioSpec Products, Bartlesville, OK). Cell debris and beads were removed by centrifugation at 11 000× *g* for 40 min at 4 °C. The polyhistidine-tagged LmbB1 protein was purified from cell-free crude extract (CFCE) using manual Ni-NTA affinity chromatography (HisPur Ni-NTA Resin, Thermo Scientific) with wash buffer [50 mM NaH_2_PO_4_, 300 mM NaCl, 10–20 mM imidazole, pH 8.0] and elution buffer [50 mM NaH_2_PO_4_, 300 mM NaCl, 250–350 mM imidazole at pH 8.0]. The CFCE containing *Shj*DDO was first diluted with lysis buffer and then filtered through 1.0- and 0.22-μm filters before being purified using an automated Ni-NTA affinity chromatography system (AKTA Go, Cytiva) and an EconoFit Profinity IMAC cartridge (5 mL, Bio-Rad) with binding buffer [50 mM KH_2_PO_4_, 300 mM KCl, 10 mM imidazole, pH 8.0] and elution buffer [50 mM KH_2_PO_4_, 300 mM KCl, 250 mM imidazole, pH 8.0]. Following purification, the protein was frozen at −80 °C in elution buffer with 10% glycerol.

### Reconstitution of l-DOPA 2,3-Dioxygenases

Purified l-DOPA 2,3-dioxygenase in elution buffer exhibited no detectable enzymatic activity and, therefore, required reconstitution with Fe^II^ prior to performing any enzymatic assays. The concentration of purified LmbB1 protein was first measured by absorbance at 280 nm (*A*_280_) after dilution in freshly prepared 20 mM NaH_2_PO_4_ (pH 7) with 6 M guanidine hydrochloride to denature the protein. *A*_280_ was converted to protein concentration using the Beer–Lambert law (*A*_280_ = ε*bc*), where *b* = 1 cm, *c* is the concentration in mol/L, and ε is an extinction coefficient in M^–1^cm^–1^ calculated from the protein primary sequence using eq 2.^44^ In contrast, the protein concentration of purified *Shj*DDO was determined using the Bradford assay (Coomassie Protein Plus Reagent, Thermo Fisher) because *Shj*DDO does not fully denature in guanidine hydrochloride, making the Bradford assay more suitable.

2 $$\varepsilon_{280}\left(M^{-1}{\mathrm{cm}}^{-1}\right)=\left(\#\mathrm{Tyr}\times 1280\right)+\left(\#\mathrm{Trp}\times 5690\right)+\left(\#\mathrm{Cys}-\mathrm{Cys}\times 125\right)$$

The concentration of purified l-DOPA 2,3-dioxygenase was then used to calculate the target Fe^II^ concentration for reconstitution with 3–4 times molar excess of enzyme. Iron­(II) sulfate heptahydrate was dissolved at 100-fold the target concentration in 0.6 M HCl Milli-Q water containing sodium ascorbate to ensure all dissolved iron remained in the +2 oxidation state and was then diluted into 0.5 M *N*-2-hydroxyethylpiperazine-*N*′-2-ethanesulfonic acid (HEPES) to 10-fold the target concentration to adjust pH to ∼7. Immediately, 100 μL of the 10× iron­(II) sulfate heptahydrate at ∼pH 7 was added to the purified l-DOPA 2,3-dioxygenase in elution buffer and incubated for 5–10 min on ice. Gel filtration (EconoPac 10DG, Bio-Rad) was then performed to remove residual reconstitution agents, with the enzyme passed either into a steady-state reaction buffer containing 0.05 M HEPES, 0.154 M NaCl, 10% glycerol (pH 7.5) or a stopped-flow reaction buffer contained 0.25 M HEPES, 0.154 M NaCl over a range of pH 7.25–7.35 (see Section Presteady State Kinetic Assay of l-DOPA 2,3-Dioxygenase).

Since the reconstitution occurred in a neutral, aqueous, and aerobic environment, the presence of Fe^II^ in the mixture was verified using a calibrated Ferrozine assay (MilliporeSigma), buffered to pH 5 with HPLC-grade ammonium acetate.^45^ After gel filtration, the reconstituted enzyme was aliquoted and stored at −80 °C. The Fe^2+^ concentration of the reconstituted l-DOPA 2,3-dioxygenase was reevaluated using the same Ferrozine assay before use in experiments. All concentration measurements were performed in triplicate, with errors reported as standard deviations.

### UV–Visible Spectroscopy and Steady-State Assay of l-DOPA 2,3-Dioxygenases

Purified, reconstituted l-DOPA 2,3-dioxygenases were assessed for activity by monitoring the cleavage of catecholic substrates using UV–visible spectroscopy. 3,4-Dihydroxyhydrocinnamic acid (DHHCA, **3**) was purchased from Sigma-Aldrich. 6-CyanoDHHCA (**5**) and 6-bromoDHHCA (**4**) were synthesized as described above. Michaelis–Menten steady-state constants *K*_M_, *k*_cat_, *V*_max_, and *k*_SP_ were obtained by reacting l-DOPA 2,3-dioxygenases (0.25–4.0 μM) with DHHCA/6-X-DHHCA derivative analogues in oxygen-saturated reaction buffer (50 mM HEPES, 10% glycerol, pH 7.5) and by detecting the continuous formation of the steady-state products at their respective λ_max_. *k*_SP_ or the “specificity constant” is equivalent to *k*_cat_ × *K*_M_^–1^ and defines the relative rates of turnover for competing substrates.^46^

Molecular oxygen (AirGas) was bubbled directly into the reaction buffer through a glass-bonded silica gas diffuser (Bubblemac Industries, Inc.) or as a gas mixture blended with air (AirGas). Solutions were bubbled with oxygen for several minutes prior to use and for the duration of the experiment. To determine the *K*_M_ for oxygen (i.e., *K*_MO2_), l-DOPA 2,3-dioxygenases (0.25–4.0 μM) were reacted with a saturating concentration of the catecholic substrate DHHCA/6-X-DHHCA derivatives (2–10× the *K*_M_ sufficient to achieve *k*_cat_) and various oxygen concentrations (21–100% or 270–1300 μM at 22 °C). The concentration of oxygen was varied by changing the percentage of oxygen in the blend using a galvanic oxygen sensor and oxygen monitor (AirGas) and then bubbling solutions, as described above. Molecular oxygen percentages were converted to molar concentrations using a reference temperature of 22 °C.^47^ The reaction of l-DOPA 2,3-dioxygenases with DHHCA/6-X-DHHCA derivatives as a function of oxygen concentration was followed by the appearance of the steady-state cleavage product by using UV–visible spectroscopy.

The extinction coefficients for steady-state cleavage products of DHHCA and synthetic 6-X-DHHCA derivatives were determined by reacting various concentrations of DHHCA/6-X-DHHCA derivatives at 22 °C with a 10-fold stoichiometric excess of enzyme (quantified by Fe^II^—see *Reconstitution*) in 50 mM HEPES buffer with 154 mM NaCl at pH 7.5, bubbled with 100% oxygen to a final concentration of 780–910 μM, depending on the volume of oxygenated buffer that comprised the reaction (60–70%). Substrate solutions were prepared from authentic synthetic standards using an analytical balance, and reactions were run to completion as assessed by the appearance of the steady-state product. The λ_max_ and extinction coefficients of DHHCA, 6-cyanoDHHCA, and 6-bromoDHHCA were determined herein as ε_380_ = 40 740 ± 820 M^–1^cm^–1^, ε_385_ = 33 256 ± 484 M^–1^cm^–1^, and ε_380_ = 35 282 ± 1190 M^–1^cm^–1^, respectively (Figure S52).

### Nonlinear Least-Squares Fitting

Steady-state kinetic data were obtained through a UV–visible spectroscopic assay. Initial rates were obtained from the slopes of the linear regions of the progress curves and plotted versus substrate concentration to yield a hyperbola, which was analyzed using the Michaelis–Menten expression and nonlinear least-squares fitting (NLSF) with Origin 6.0 (Microcal). Two possible derivations of the Michaelis–Menten expression were used: 1) the traditional expression *y* = *k*_cat_ × *x*/(*K*_M_ + *x*), where *y* is Rate/*E*_0_ or the rate of product formation (μM/min) divided by the starting enzyme concentration (μM), *x* is substrate concentration (μM) and *k*_SP_ (also known as *k*_cat_ × *K*_M_^–1^)—that is, the second-order rate constant of enzyme and substrate—is extracted from the fitted constants; or 2) the alternative derivation^46^ that extracts *k*_SP_ and *k*_cat_ directly from the fit: *y* = (*k*_SP_ × *x*)/((1 + *k*_SP_ × *x*)/*k*_cat_), and where *y* is Rate/*E*_0_ or the rate of product formation (μM/min) divided by the starting enzyme concentration (μM), *x* is substrate concentration (μM), *k*_SP_ is equivalent to *k*_cat_×*K*_M_^–1^, and *K*_M_ is extracted from *k*_cat_/*k*_cat_×*K*_M_^–1^.

### Homology Model Building, Small Molecule Docking, and SurfNet Calculations

The *S. sclerotialus* l-DOPA 2,3-dioxygenase holo structure^38^ (SsDDO, PDB: 6ON3) was modified by isolating one dimer and deleting l-DOPA from the active site. The sequences for the l-DOPA 2,3-dioxygenase from *S. lincolnensis* (LmbB1; accession code: CAA55747.1) and *S. hygroscopicus* *jingganensis* (*Shj*DDO; accession code: AEY93898) were aligned with the sequence for SsDDO/6ON3, and the alignment was used to predict a structural model for each homologue using Modeller.^48^ Substrate structures were drawn and MM2 energy-minimized using Chem3D. Net formal charges of −2, −1, and 0 were applied to each 6-X/DHHCA derivative using Chem3D, and atomic partial charges were computed using the AM1-BCC method.^49^ The receptor (i.e., the homology models of *Shj*DDO and LmbB1) and ligand structures (i.e., catecholic substrates) were prepared using the dock prep function in UCSF Chimera.^50^

Ligand structures were docked into the active site of each l-DOPA 2,3-dioxygenase homology model using AutoDock Vina.^51^ The search volume was centered on the ligand-binding site using grid box dimensions of 13.0 × 9.5 × 11.0 Å. Poses were qualitatively evaluated based on their scores and pose similarity to the natural orientation of l-DOPA in the published 6ON3 crystal structure. Poses were quantitatively evaluated by measuring the distances from the catecholic oxygens of the ligand to the Fe^II^, Y141 (chain B), H72 (chain A), and Thr51 (chain A) for the model of *Shj*DDO and Y139 (chain B), H69 (chain A), and Thr48 (chain A) for the model of LmbB1.

SurfNet calculations were performed within UCSF Chimera on the models of *Shj*DDO and LmbB1 by visually identifying the residues lining the active site pocket in both models and defining those residues—either whole residue with side chain or the backbone Cα and N depending on how the residue was oriented relative to the pocket—to the SurfNet calculation^52^ as follows: for the model of *Shj*DDO, atom Set 1 (i.e., chain B):128–138 and Atom Set 2 (i.e., chain A): 42,44,46,47:43,45@CA,N. For the model of LmbB1, atom Set 1 (i.e., chain B):126–136 and atom Set 2 (i.e., chain A): 39,41,43,44:40,42@CA,N. The residues used in the calculation for each model were located in the same space according to the sequence and structural alignment.

### Presteady State Kinetic Assay of l-DOPA 2,3-Dioxygenase

Presteady state observation of *S. hygroscopicus* *jingganensis* l-DOPA 2,3-dioxygenase (*Shj*DDO) reaction with DHHCA (**3**) and 6-cyanoDHHCA (**5**) was carried out on an SF300x stopped-flow spectrometer (KinTek Corporation). Reaction temperature was controlled at 14 ± 0.5 °C by a circulating water bath. Reactions were performed in HEPES (250 mM) and NaCl (154 mM) titrated with HCl or NaOH such that the pH would be 7.35 or 7.25 at the reaction temperature. Stock solutions of DHHCA (**3**) were prepared in acidified Milli-Q water (pH ∼ 3) to prevent oxidation. Stock solutions of 6-cyanoDHHCA (**5**) were prepared in DMSO. The reaction buffer was bubbled with 100% O_2_ in an ice bath for 10–20 min, and DHHCA (**3**) or 6-cyanoDHHCA (**5**) solutions were prepared by diluting the stock into the oxygenated buffer to the desired concentration immediately prior to loading on the instrument. *Shj*DDO was reconstituted with iron­(II) sulfate heptahydrate, gel filtered into the reaction buffer, and loaded into the drive syringe. For each reaction, the instrument mixed 20 μL of each reactant (1:1 dilution), which was observed at 380 nm to detect the formation of the steady-state products. Fifteen to twenty progress curves from the same experimental conditions were averaged in the KinTek Stopped Flow software and exported to KinTek Explorer for modeling. Between experiments, the instrument drive syringes were cleaned with acid, base, and water; soaked in 1% Hellmanex detergent for 30 min at ∼30–35 °C; thoroughly rinsed with water; and stored in 10% ethanol.

### Modeling of the Presteady State Kinetic Mechanism

Presteady state kinetic data were analyzed by fitting to simulated mechanisms using the KinTek Explorer software (KinTek Corp, Austin, TX).^53^ KinTek Explorer simulates experimental results by using direct numerical integration of rate equations for the kinetic model and yields estimates for the rate constants of microscopic steps. Presteady state experiments were modeled using KinTek Explorer according to a defined reaction pathway, as described in the Results and Discussion and shown in Scheme 1. The extinction coefficients of the DHHCA and 6-cyanoDHHCA semialdehyde products were independently determined as described above and fixed during fitting. Some rates were fixed as either “fast” (i.e., 1 × 10^9^) or very slow (1 × 10^–12^, i.e., ∼0) as described in the text. The equilibrium represented by *k*_1_ and *k*_–1_ was fixed at the ratio of substrate charge states as predicted by the experimentally determined p*K*_a_ values (Table 1) and the Henderson–Hasselbach equation. Extinction coefficients were fitted and/or fixed during fitting as described in the text and interpreted using the observable expression: a*­(ESQ–ESQ_X) + b*­(EP+P) – *c*, where *a* is the extinction coefficient applied to ESQ, *b* is the independently experimentally determined extinction coefficient on P, and *c* is a *y*-axis offset. E and S_A and S_B are the enzyme and two charge states of the substrate, in this case binding prior to O_2_; however, the software consistently drove one of the pathways to the ES complex to zero, where it was fixed during FitSpace calculations. ESO_2_ is the ternary complex of enzyme, substrate, and oxygen. ESQ represents the putative semiquinone intermediate, a UV-absorbing species, and ESQ_X is the species formed from the nonproductive quenching of the semiquinone. EI represents subsequent intermediates that are UV-inactive. The Fe-alkylperoxo intermediate is predicted to follow the semiquinone (Figure 3), but this intermediate and additional intermediates are UV-inactive, and therefore rates surrounding their appearance and disappearance are not constrained by these data; therefore, the rate for EI = EP represents all the rates for the appearance and disappearance of UV-inactive species between the semiquinone intermediate and the semialdehyde product (P). EP is the enzyme–product complex, and P is the free semialdehyde product. P can then degrade nonenzymatically to the spectroscopically silent P_X.

**Scheme 1:**
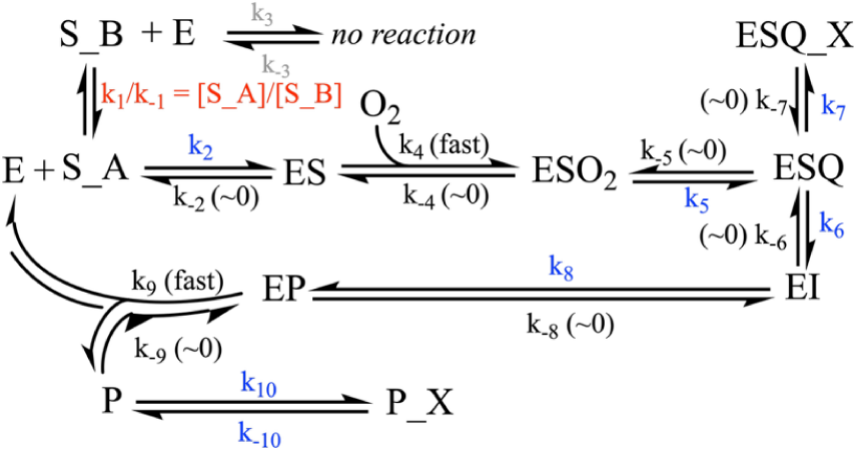
Reaction Pathway used for Modeling the Presteady State Experiments

**Table 1:** p*K*_a_ Values for l-DOPA, DA, DHHCA, and Synthetic Derivatives Determined Spectroscopically from pH 3 to 12.5^a^

	p*K*_a_ value	p*K*_a_ value of dopamine counterpart^42^
l-DOPA (**1**)	8.81 ± 0.19	-
Dopamine (DA, **2**)	8.99 ± 0.05	-
DHHCA (**3**)	8.87 ± 0.12	-
6-BromoDHHCA (**4**)	8.36 ± 0.07	8.09 ± 0.03
6-CyanoDHHCA (**5**)	7.51 ± 0.06	7.03 ± 0.06
6-NitroDHHCA (**6**)	6.94 ± 0.06	6.23 ± 0.03

a Dopamine data reprinted with permission from *Biochemistry* 2021, 60, 32, 2492–2507. Copyright 2021 American Chemical Society.

**Figure 3:**
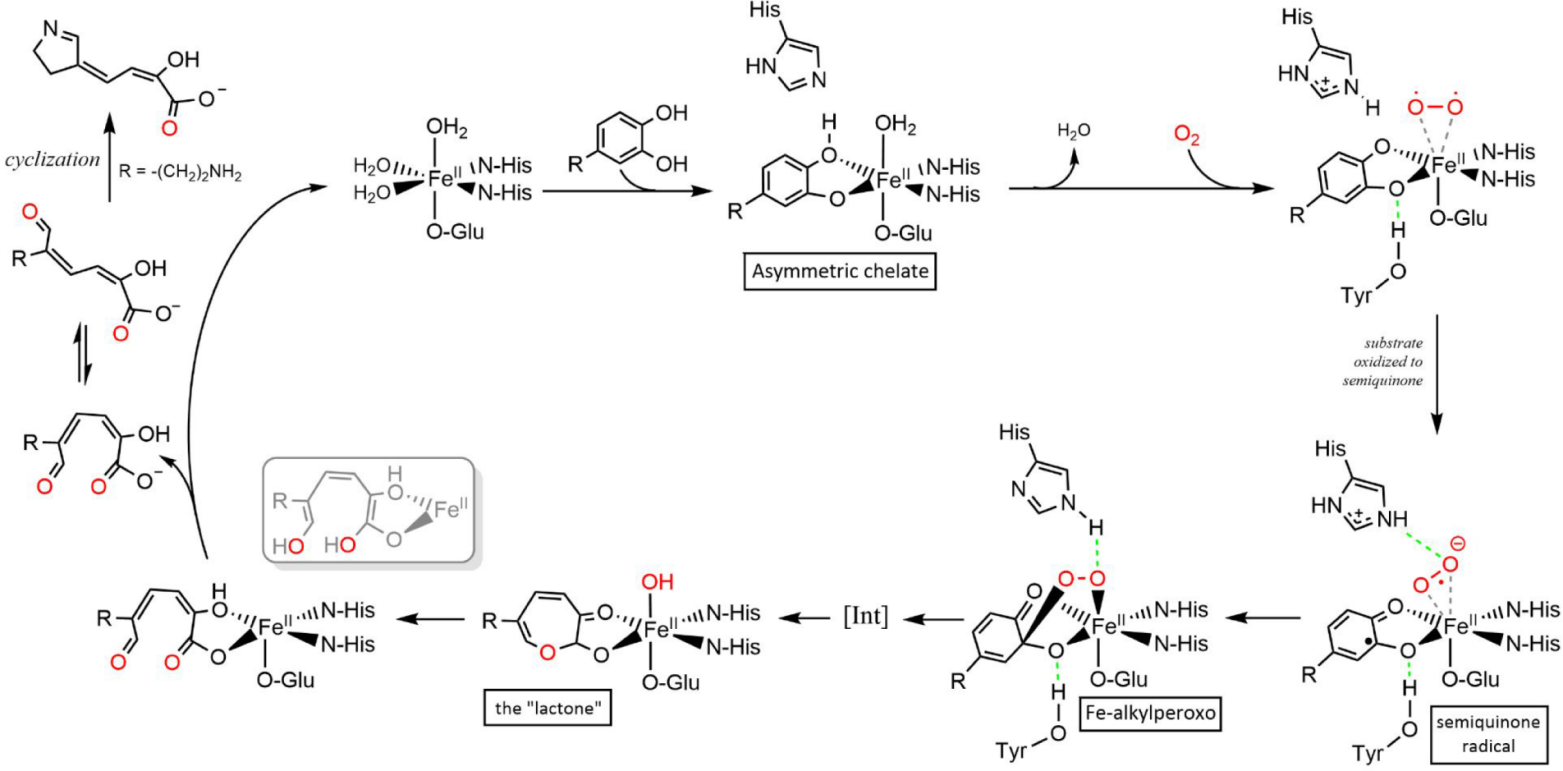
A proposed mechanism for EDX cleavage of a catecholic substrate within the active site of an extradiol dioxygenase.^58,59,67,68^ The cycle begins when the substrate catechol is proposed to bind as a monoanion, displacing two molecules from around the Fe^II^ to form the asymmetric chelate. The monoanionic nature of the substrate is a function of the catecholic p*K*_a_ value. Alternative substrates with low catecholic p*K*_a_ values can bind as a dianion. The oxygen molecule binds to the Fe^II^ molecule, displacing the final water molecule. Subsequently, the substrate is oxidized to the semiquinone radical via the metal ion. The radical iron-bound superoxo species and the semiquinone radical recombine to yield the Fe-alkylperoxo intermediate. O–O and C–C bond cleavage affords a ring expansion which likely proceeds in a stepwise manner through several additional intermediates (not shown).^28,59^ The resulting lactone is hydrolyzed by the metal-bound hydroxide to afford the ring-opened product. Tautomerization to the enol form is possible,^59^ and the ring-opened product likely isomerizes upon release from the enzyme. If the R group contains an amino functionality, then cyclization to an imine follows. His, Glu, and Tyr are conserved active site residues. Reprinted with permission from *Biochemistry* 2021, 60, 32, 2492–2507. Copyright 2021 American Chemical Society.

Errors on each fit were calculated from 2D Fitspace^54^ at 0.83 Chi^2^. The KinTek Explorer program FitSpace explores the uniqueness of a particular fit to the data by measuring the dependence of the sum square error (SSE) on each pair of parameters, while allowing all other parameters to vary in seeking the best fit.^54^ The confidence intervals reported for each of the fitted parameters are determined by a Chi^2^ Threshold of 0.83 (SSE_x,y_/SS*E*_min_) and include the range of values for which the fits have X^2^ that deviate values no more than 20% from the lowest X^2^ value. These confidence intervals are also represented as two-dimensional plots (confidence contours) of the reciprocal sum square error (SS*E*_min_/SSE_x,y_); constrained parameters have a well-defined region in which the SSE_x,y_ approaches SS*E*_min_ (red in a contour plot).

## Results and Discussion

### Synthesis of 3,4-Dihydroxyhydrocinnamic Acid (DHHCA) Derivatives

As we have noted previously,^42^ careful study of extradiol dioxygenase activity has been historically limited by the availability of sterically and electronically diverse substrates to probe reactivity and mechanism. 3,4-Dihydroxyhydrocinnamic acid (DHHCA, **3**), also known as hydrocaffeic acid, is a catecholic example of lignin-derived carbon^40,41^ that is similar in structure to the preferred substrate of l-DOPA 2,3-dioxygenase, l-3,4-dihydroxyphenylalanine (l-DOPA, **1**), and is also amenable to synthetic functionalization of the aromatic ring. The design and synthesis of 6-substituted derivatives of DHHCA not only add to the toolbox of electronically and sterically diverse catecholic substrates of EDXs but also shed light on a possible proclivity of l-DOPA 2,3-dioxygenases to cleave lignin-derived aromatic carbon.

The synthesis of 6-bromoDHHCA (**4**) was accomplished according to Scheme 2 in an overall 28% yield from the starting material 3-(4,5-dimethoxyphenyl) propanoic acid (DMPPA). The design of Scheme 2 is based on our previous work.^42,55^ Although we were able to brominate DMPPA directly using bromine in acetic acid in 45% yield, we found that it resulted in a final product that was not easy to handle, and we determined that the DMPPA needed to first be esterified. Therefore, the starting material, DMPPA, was esterified using thionyl chloride in methanol, producing **7** in an 87% yield.^56^ Compound **7** was then brominated to yield **8**. The ester was cleaved before the methyl ethers were removed by treatment with a 1 M solution of boron tribromide in dichloromethane, resulting in the desired product 6-bromoDHHCA (**4**) in quantitative yield. The final product did not precipitate like 6-bromodopamine due to the lack of salt formation, but a solid was obtained by dissolving the crude product in anhydrous diethyl ether, separating it, and concentrating it under reduced pressure.

**Scheme 2:**
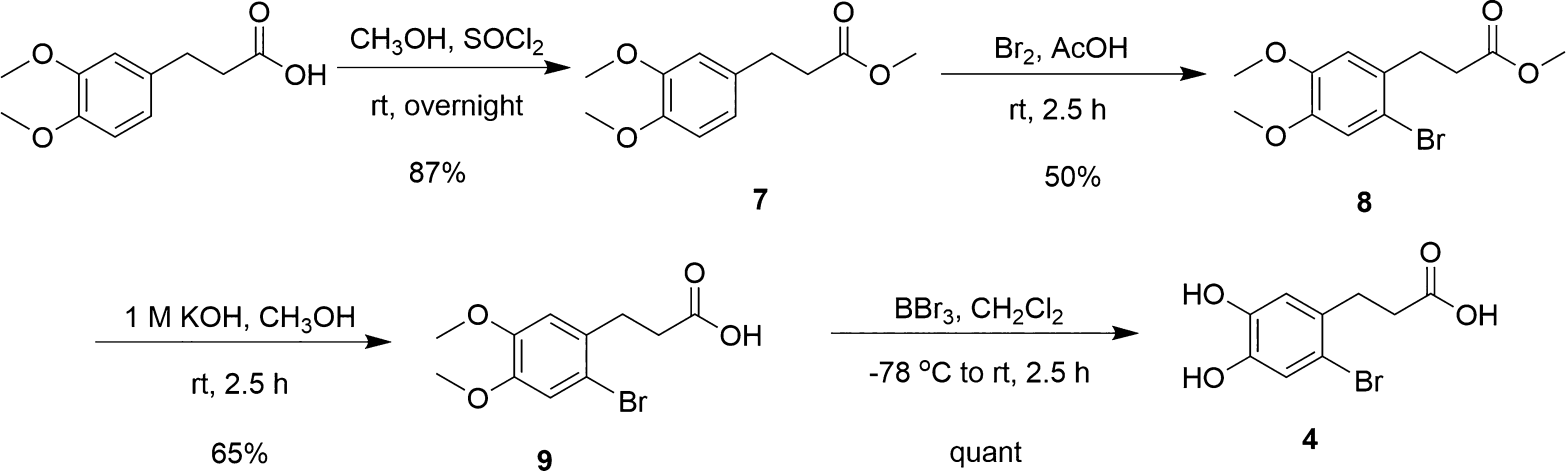
Synthesis of 6-BromoDHHCA (**4**)

The synthesis of 3-(2-cyano-4,5-dihydroxyphenl) propanoic acid (6-cyanoDHHCA, **5**) was accomplished according to Scheme 3 in an overall 45% yield from **7**. The iodine was installed with iodine monochloride in acetic acid, yielding **10** in 78% yield. Unfortunately, we could not find column conditions for the separation of **7** and **10**, and therefore, it was essential to run the reaction to completion before moving to the next step. The iodo substituent was then converted to a cyano group using copper­(I) cyanide to yield **11**.^55^ As with the bromo analogue, de-esterification of **11** with sodium hydroxide successfully resulted in 6-cyanoDMPPA (**12**).^56^ Subsequent treatment with boron tribromide converted 6-cyanoDMPPA to 6-cyanoDHHCA (**5**), our intended product, in 91% yield.

**Scheme 3:**
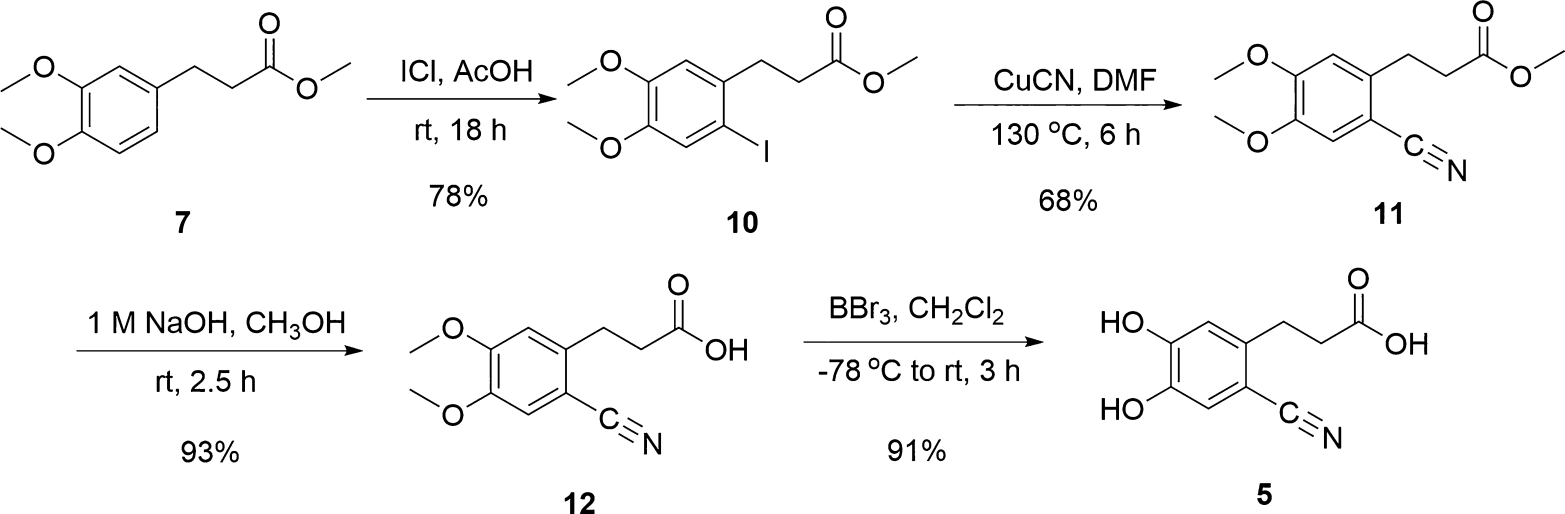
Synthesis of 6-CyanoDHHCA (**5**)

3-(4,5-Dihydroxy-2-nitrophenyl) propanoic acid (6-nitroDHHCA, **6**) was synthesized from DHHCA (**3**) as shown in Scheme 4. Initial attempts at the synthesis of **6** were unsuccessful as the nitrated product did not precipitate and was not easily isolated as previously observed for the structurally analogous 6-nitrodopamine.^55^ Additionally, the use of 20–30% sulfuric acid^55^ did not yield the desired result. Successful mononitration of **3** to **6** was finally accomplished with a shorter reaction time (i.e., 15 min), a lower sulfuric acid concentration (i.e., 5%), and a colder reaction temperature (i.e., 0 °C). These conditions were imperative to avoid further nitration of the catechol on the other open aromatic carbons or oxidation of the catechol core. The product was extracted using ethyl acetate, yielding **6** as an orange solid with a 51% yield.

**Scheme 4:**
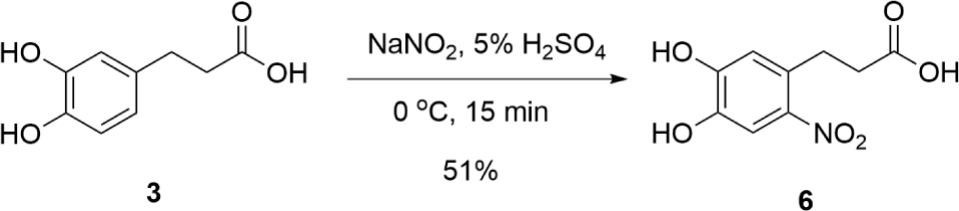
Synthesis of 6-NitroDHHCA (**6**)

### Characterization of Acidity and Redox Potential for 6-Substituted DHHCA Derivatives

Existing studies on the EDX mechanism predict that deprotonation of the catecholic oxygen at carbon-3 of the catecholic ring is an essential step in substrate binding and precedes a one-electron oxidation of the ring to the semiquinone radical (Figure 3).^57–59^ Indeed, our own work has underscored the relevance of substrate p*K*_a_ values and one-electron oxidation potentials to the rates of enzymatic cleavage by l-DOPA 2,3-dioxygenase. Building on this previous work, p*K*_a_ values for acidic protons were measured along with the one-electron redox potentials of 6-substituted DHHCAs.

The p*K*_a_ values for the first catecholic deprotonation of l-DOPA (**1**), dopamine (**2**), DHHCA (**3**), and the synthetic 6-substituted DHHCA derivatives were determined by spectrophotometric titration over the pH range from 3 to 12.5 (Table 1). The p*K*_a_ values for the first catecholic deprotonation for l-DOPA (**1**), DA (**2**), and DHHCA (**3**) are similar to the literature values.^60,61^ As expected, the p*K*_a_ values for the synthetic DHHCA derivatives became more acidic as the electron-withdrawing capacity of the 6-substituent increased (Table 1). The p*K*_a_ values in Table 1 were compared with the Hammett sigma *para* constants and the Hammett sigma *meta* constants (Figure S2). Although the *R* ^2^ values are both greater than 0.9, the correlation was stronger with the sigma *para* constants (*R* ^2^ = 0.9806), consistent with the initial deprotonation of the C3-OH, which is *para* to the substituent at the 6-position.^62^

These data confirm that 6-X-DHHCA substituents are electron-withdrawing groups, but the effect of that electron withdrawal is mitigated by the presence of the carboxylate, which is undoubtedly ionized under physiological conditions. Therefore, p*K*_a_ values for the 6-X-DHHCAs are less acidic relative to those of the similarly substituted dopamine due to the perturbation of the propanoate side chain. Conversely, the ethanamine side chain on each similarly substituted dopamine contributes a positive charge at physiological pH due to a p*K*_a_ value of ∼10 for the primary amine.^60^ Therefore, the energetic cost of deprotonating the C3-OH of 6-X-DHHCA is higher than the corresponding deprotonation of the similarly substituted dopamine because it adds a second negative charge to the conjugate base.

Following the predicted deprotonation of catecholic oxygen at C3 of the catecholic ring,^63–65^ the semiquinone radial is formed by a one-electron oxidation of the ring mediated by the active site Fe^II^.^66^ When examining a variety of substrate derivatives, we have observed that experimental measurements of the oxidation potential by cyclic voltammetry are essential for predicting the ability of the enzyme to oxidatively cleave a given substrate.^42^ As is the case for deprotonation and C3-OH p*K*_a_ value, the nature of the side chain (i.e., amino acid, amine or carboxylic acid) and the nature of the substituent at the 6-position of synthetic substrates both affect the redox potential (Figure 3). While electron-withdrawing substituents at the 6-position create substrates that are generally more difficult to oxidize, experimental measurements of redox potential indicate that the presence of varied side chain motif complicates this interpretation.

The redox properties of l-DOPA (**1**), DA (**2**), and DHHCA (**3**) and 6-substituted DHHCA analogues (Table 2) were determined at pH 6.0 and compared to the redox properties of similarly substituted dopamine analogues^42^ at pH 7.4. Both DHHCA and the similarly substituted dopamine derivative are modified at the 6-position of the ring, but the DHHCA derivatives have a propanoate side chain at the 1-position, while the dopamine analogues have an ethanamine side chain, and l-DOPA has both functional groups. Interestingly, DA (**2**) is much easier to oxidize than both the carboxylic acid containing l-DOPA (**1**) and DHHCA (**3**).

**Table 2:** Anodic (Oxidation (*E*_p_)_a_) and Cathodic (Reduction (*E*_p_)_c_) Peak Potentials from Cyclic Voltammograms of Dopamine or Dopamine Derivative Solutions at pH 6.0 and 7.4 on a GCE (*d* = 2 mm)

	pH 7.4					pH 6.0				
Compound	(*E*_p_)_a_ Oxidation (mV)	(*E*_p_)_c_ Reduction (mV)	*E*_1/2_ (mV)	Δ*E*_p_ (mV)	*I*_pa_/*I*_pc_	(*E*_p_)_a_ Oxidation (mV)	(*E*_p_)_c_ Reduction (mV)	*E*_1/2_ (mV)	Δ*E*_p_ (mV)	*I*_pa_/*I*_pc_
l-DOPA (1)	315	-	-	-	-	362	101	231.5	261	5.90
DA (2)	178	54	116	124	1.32	354	84	219	270	2.89
DHHCA (3)	333	–95	119	428	1.30	388	–9	198.5	379	2.54
6-Bromo DHHCA (**4**)	195	59	127	136	1.21	267	154	210.5	113	1.43
6-Cyano DHHCA (**5**)	272	147	209.5	125	1.96	358	260	309	98	3.92
6-Nitro DHHCA (**6**)	288	-	-	-	-	444	-	-	-	-

The oxidation potential ((*E*_p_)_a_) of the DHHCA analogues increased as the 6-substituents became more electron-withdrawing, consistent with previous observations of the dopamine (DA) analogues.^42^ The results of the DHHCA derivatives with strong electron-withdrawing groups, 6-cyanoDHHCA (**5**) and 6-nitroDHHCA (**6**), having the lowest catecholic p*K*_a_ value is consistent with the high oxidation potentials, meaning the compound is likely to be deprotonated at the C3-OH as a catecholic monoanion. 6-BromoDHHCA (**4**) has a lower oxidation potential, which was confirmed when handling the compound, and is likely due to the electron-donating ability of the bromo substituent.

### Steady-State Kinetics of DHHCA and 6-X-DHHCA Derivatives with l-DOPA 2,3-Dioxygenases

To assess the activity of l-DOPA 2,3-dioxygenases on DHHCA and its 6-substituted derivatives, the aerobic, enzymatic reaction was observed by UV–visible spectroscopy. The extradiol cleavage of DHHCA and its derivatives produces a yellow semialdehyde that is readily visualized at or around 380 nm with a λ_max_ that is substrate dependent. When a steady-state product was observed, the enzymatic reaction was interrogated with steady-state kinetic methods as a means of comparing substrates.

DHHCA (**3**), 6-bromoDHHCA (**4**), and 6-cyanoDHHCA (**5**) exhibited measurable activity with both *S. lincolnensis* l-DOPA 2,3-dioxygenase (i.e., LmbB1) and the l-DOPA 2,3-dioxygenase from *S. hygroscopicus* *jingganensis* (i.e., *Shj*DDO) and steady state kinetics of both the catecholic and oxygen substrates were measured, and these results are summarized in Table 3 and Figures S53–S56 and analyzed in Table 4. In comparison, no turnover was detected for 6-nitroDHHCA (**6**). The purpose of measuring activity on two homologues of l-DOPA 2,3-dioxygenase was 2-fold: 1) to examine the extent to which either dioxygenase: the original l-DOPA 2,3-dioxygenase belonging to lincomycin biosynthesis,^31,32^ or a more recently identified enzyme from *S. hygroscopicus* *jingganensis*,^39^ exhibited activity on an example of lignin-derived carbon (i.e., DHHCA) and measure the resilience of that activity to electronic perturbation in the form of electron-withdrawing substituents; and 2) to probe the interface of active site volume and catalysis by evaluating the same lignin-derived catecholic substrates across two enzymes. The second goal is part of a larger effort to understand the barriers to harnessing EDX chemistry, namely, the propensity for enzymatic inactivation and variability in activity on the same substrates between active sites.

**Table 3:** Steady-State Kinetic Parameters for DHHCA and 6-X-DHHCA Derivatives with l-DOPA 2,3-Dioxygenases from *S. lincolnensis* and *S. hygroscopicus* *jingganensis*

Compound	l-DOPA 2,3-dioxygenase homologue	*K*_M_ DHHCA/6-X-DHHCA (μM) at 100% O_2_ saturation	*k*_cat_ (sec^–1^) at 22 °C and 100% O_2_ saturation	*k*_SP catechol_ × 10^2^ at 22 °C (μM^–1^ min^–1^)	*K*_M_ O_2_ (μM)	*k*_SP,O2_ × 10^2^ at 22 °C (μM^–1^ min^–1^)
DHHCA (**3**)	*S. lincolnensis*	_922 ± 152_	_2.1 ± 0.2_	_13.4 ± 2.5_	_192 ± 33_	_19.2 ± 3.2_
	*S. hygroscopicus* *jingganensis*	_422 ± 94_	_6.7 × 10_^–1^ _± 5.0 × 10_^–2^	_9.5 ± 2.3_	_1059 ± 246_	_17.7 ± 2.6_
6-BromoDHHCA (**4**)	*S. lincolnensis*	_205 ± 24_	_1.6 × 10_^–1^ _± 4.6 × 10_^–3^	_4.6 ± 0.5_	_280 ± 45_	_3.5 ± 0.5_
	*S. hygroscopicus* *jingganensis*	_926 ± 169_	_1.5 ± 0.1_	_9.8 ± 1.9_	_759 ± 66_	_15.8 ± 1.0_
6-CyanoDHHCA (**5**)	*S. lincolnensis*	_131 ± 16_	_6.7 × 10_^–4^ _± 2.8 × 10_^–5^	_3.1 × 10_^–2^ _± 3.9 × 10_^–3^	_563 ± 150_	_1.4 × 10_^–2^ _± 3.0 × 10_^–3^
	*S. hygroscopicus* *jingganensis*	_228 ± 53_	_1.1 × 10_^–3^ _± 1.0 × 10_^–4^	_2.8 × 10_^–2^ _± 7.1 × 10_^–3^	_1019 ± 236_	_4.0 × 10_^–2^ _± 6.0 × 10_^–3^

**Table 4:**
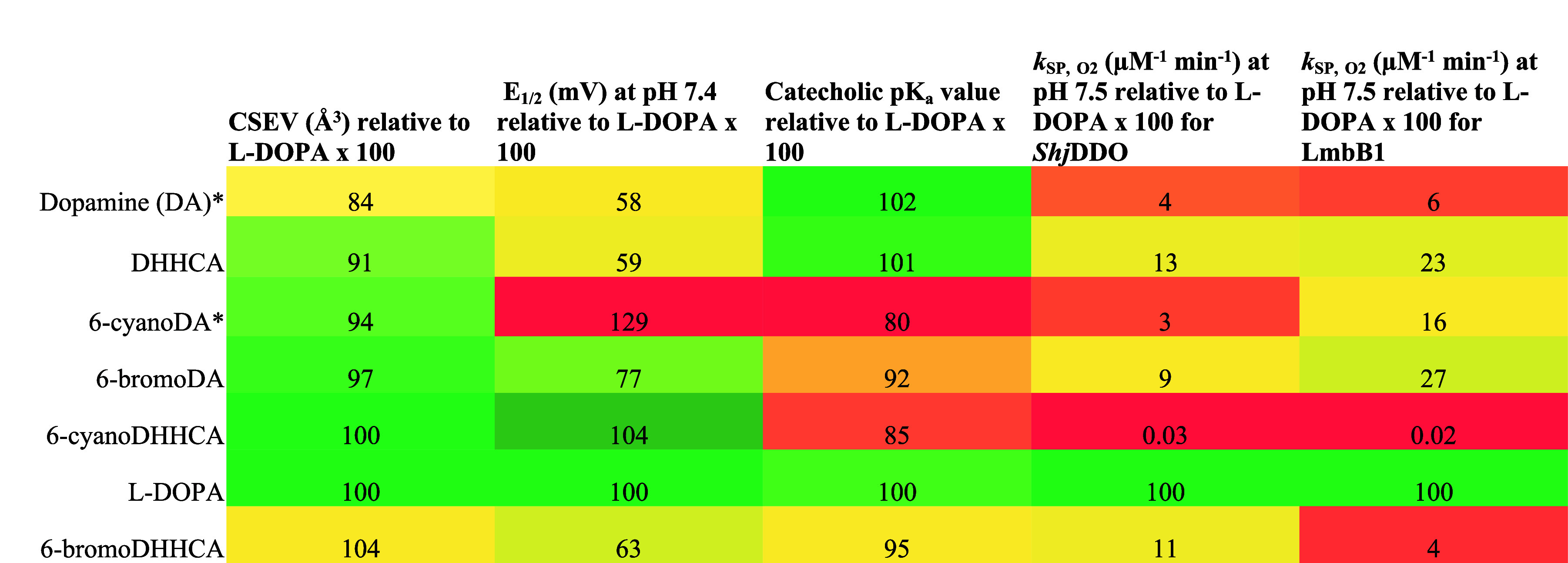
Physical Properties of Catecholic l-DOPA 2,3-Dioxygenase Substrates and Rates of Reaction with the Enzyme and Cosubstrate Oxygen Collectively Expressed as a Percentage Relative to the Value Determined for l-DOPA^a^

a The cells of the table are colored to express the comparison to l-DOPA, with parameters similar to or equal to those of l-DOPA colored green (100%) and parameters different to that of l-DOPA colored a different shade of green, yellow, or red (≠100%). Physical property CSEV (Å^3^) is shown in column 2, where substrates different in volume than l-DOPA are colored yellow (≠100%). Redox potential, as represented by *E*_1/2_ (mV), is shown in column 3. Catechols that are more difficult to oxidize (i.e., have a higher *E*_1/2_) than l-DOPA are colored red (>100%), while those that are easier to oxidize (i.e., have a lower *E*_1/2_) than l-DOPA are colored yellow (<100%). The p*K*_a_ value for the catecholic proton is shown in column 4. Substrates with p*K*_a_ values slightly less acidic relative to l-DOPA are colored darker green (>100%), while those more acidic than that of l-DOPA are colored yellow to red (<100%). Lastly, *k*_SP,O2_ rates (μM^–1^ min^–1^) of catecholic substrates with the cosubstrate oxygen are expressed for the two l-DOPA 2,3-dioxygenase enzymes from *S. hygroscopicus* *jingganensis* (*Shj*DDO) and *S. lincolnensis* (LmbB1) in columns 4 and 5, respectively. Catechols that react with l-DOPA 2,3-dioxygenase and the cosubstrate oxygen with a *k*_SP,O2_ rate slower than that of l-DOPA are colored red (<100%). *Adapted with permission from *Biochemistry* 2021, 60, 32, 2492–2507. Copyright 2021 American Chemical Society.^42^

Taking a broad view of the steady-state parameters for DHHCA (**3**), 6-bromoDHHCA (**4**), and 6-cyanoDHHCA (**5**) across both enzymes, upon initial inspection, there are two clear observations. First, the *K*_M_ values for *Shj*DDO are—on the whole—weaker than the *K*_M_ values for LmbB1, which implies that *Shj*DDO may be a less discriminating enzyme. Second, it is readily apparent that 6-cyanoDHHCA (**5**) is the weakest substrate in total. The *k*_cat_, *k*_SP,catechol_ (i.e., *k*_cat_/*K*_M_), and *k*_SP,O2_ for 6-cyanoDHHCA (**5**) are the slowest for both enzymes. Examining the p*K*_a_ values (Table 1) and *E*_1/2_ values (Table 2) confirms that 6-cyanoDHHCA (**5**) has the lowest p*K*_a_ value and the highest *E*_1/2_ among the substrates that produced any turnover with either enzyme. Only 6-nitroDHHCA (**6**) has a lower p*K*_a_ value and is more difficult to oxidize, and 6-nitroDHHCA (**6**) did not undergo turnover with either enzyme. As expected, oxidizability is a significant variable in predicting reactivity with l-DOPA 2,3-dioxygenases; however, oxidizability is not the only variable at work in this system. As shown in Table 2, 6-bromoDHHCA (**4**) is readily oxidized, more so than DHHCA (**3**), and in Table 3, the *K*_M_ for 6-bromoDHHCA is 4.5-fold tighter than DHHCA. Yet, 6-bromoDHHCA is a poorer substrate than DHHCA in the LmbB1 active site as it is slower by more than 10-fold in *k*_cat_ and more than 5-fold by *k*_SP,O2_. In contrast, the *Shj*DDO active site accommodates 6-bromoDHHCA (**4**) with a *K*_M_ that is 2-fold weaker, but a *k*_cat_ that is 2-fold faster than DHHCA, and a *k*_SP,O2_ that is equivalent to DHHCA (**3**) within error. In order to interpret these data and provide a governing hypothesis of substrate compatibility with l-DOPA 2,3-dioxygenase, we took the following three-part approach: 1) situate these steady-state data (Table 3) within the larger body of steady-state data that we have accumulated on these enzymes and other structurally analogous substrates, 2) interpret these data in the context of the unique enzyme active site using homology modeling and docking, and 3) apply presteady state examination to interrogate our hypotheses.

### Integrated Analysis of Substrate Physical Properties with Steady-State Enzyme Kinetics and Docking

A comprehensive model of substrate compatibility with l-DOPA 2,3-dioxygenases, which is broadly applicable to other homologous enzymes, must consider the physical properties of each catecholic substrate, including redox potential, p*K*_a_ value, and size, alongside kinetic parameters describing the reaction in a particular active site. To capture the significance of redox potential, p*K*_a_ value, and substrate size relative to the enzyme under study, the physical properties of synthetic and commercially available compounds used in this study (Tables 1 and 2) and in our previous work are represented in Table 4 as a percentage of the experimental value relative to the catecholic substrate, l-DOPA. The catechol l-DOPA was chosen as the standard for comparison because l-DOPA is consistently the most effective enzymatic substrate in terms of *k*_SP,O2_ (μM^–1^ min^–1^). Redox potential was represented with *E*_1/2_ because this is the property that correlates with the p*K*_a_ value (Figure S69). While there are several kinetic parameters (e.g., *k*_cat_, *k*_SP_) that could arguably be chosen as the standard for comparison, we have chosen *k*_SP,O2_ for two reasons: *k*_SP_ is equivalent to *k*_cat_/*K*_M_ ^46^ and expresses a second-order rate constant for the reaction of enzyme and substrate; it is therefore the most informative of the steady-state parameters when asking questions of catalytic efficiency between enzyme active sites, and 2) because the l-DOPA 2,3-dioxygenase reaction has two substrates: catechol and molecular oxygen, we chose *k*_SP,O2_ because the reaction of oxygen with the substrate is the most inactivation-prone step of the reaction.^39^ As shown in Figure 3, the catecholic substrate must be activated by oxidation to the semiquinone and the cosubstrate oxygen is activated by reduction to superoxide, both via the active site Fe^2+^. The predicted quenching of the semiquinone comes by radical recombination with the superoxide to generate the Fe-alkylperoxo intermediate. We have observed inactivation of l-DOPA 2,3-dioxygenase likely attributable to this step because consumption of oxygen occurs without ring cleavage.^39^ It is therefore consistent that any compromise to the radical recombination of superoxide and semiquinone (Figure 3) should be reflected indirectly in the steady-state *k*_SP,O2_ and directly in the formation and decay of the semiquinone observable by presteady-state methods (see Presteady-State Kinetics of DHHCA and 6-CyanoDHHCA Derivative with l -DOPA 2,3-Dioxygenase section).

Furthermore, using the aforementioned understanding of the mechanism, we can see that the redox potential, p*K*_a_ value, and size of the catecholic substrate contribute independently and cumulatively to the success of the reaction. A substrate that is more difficult to oxidize (e.g., 6-cyanoDHHCA, **5**) should be kinetically delayed in accumulating the semiquinone. Conversely, a substrate that is small relative to the available active site volume (i.e., dopamine, **2** ^42^ or DHHCA, **3**) results in too much available active site space for activated oxygen to sample. The excess space makes it less likely that the activated oxygen (i.e., superoxide) adopts the binding mode that results in effective radical recombination to form the Fe-alkylperoxo and progression of the reaction to cleavage product, and more likely that the superoxide escapes the active site, destructively terminating the reaction pathway. This integrated understanding of catecholic substrate oxidizability, active site volume, and the effective reaction of activated oxygen with the semiquinone could be summarized as a “Goldilocks principle”. A “Goldilocks principle” is one in which several variables must be ideally tuned to accomplish an objective.^69^ In this case, the objective is the successful combination of the catecholic substrate and molecular oxygen within a given l-DOPA 2,3-dioxygenase active site. The variables are those expressed in Table 4: the size of the catecholic substrate, the oxidizability of the catecholic substrate, and the particular enzyme active site. The oxidizability of the catecholic substrate contributes to the success of the reaction pathway because a readily oxidizable substrate forms the semiquinone easily (Figure 3); however, the fate of that semiquinone—and the progress of the reaction—depends on the activated oxygen (i.e., superoxide) successfully combining with the substrate radical to generate the Fe-alkylperoxo and, ultimately, the cleavage product. If the catecholic substrate is small relative to the available active site volume and there is too much active-site space for the activated substrate and superoxide to sample, then successful radical recombination is less likely, and loss of superoxide—with inactivation—of the enzyme is more likely. This explains the behavior of dopamine, which is an easily oxidized substrate with a surprisingly fast *k*_SP,catechol_ and overall *k*_cat_, but with significant inactivation of the enzyme visible in the steady state and presteady states.^39,42^ This is consistent with our previous observations that dopamine easily forms a semiquinone but reacts inefficiently with activated oxygen.

If we turn our attention to DHHCA and the 6-X-DHHCAs, an integrated understanding of oxidizability, size, and active site—i.e., the “Goldilocks principle”—is also essential for predicting the behavior of these substrates. DHHCA (**3**) is more readily oxidized than l-DOPA (**1**), with an *E*_1/2_ that is 59% of L-DOPA’s, compared to 58% for dopamine (**2**) (Table 4). DHHCA (**3**) is also smaller than l-DOPA (i.e., 91% of the CSEV), but not as small as dopamine (Table 4). Applying the Goldilocks principle, DHHCA should have a better *k*_SP,O2_ than dopamine, and indeed, it does across both enzymes. At the other end of the spectrum is 6-cyanoDHHCA (**5**) with a *k*_SP,O2_ that is <0.1% of the activity for l-DOPA, consistent with a violation of a variable contributing to the Goldilocks principle. While 6-cyanoDHHCA is the same size as l-DOPA, it is considerably more difficult to oxidize, and this is the most plausible explanation for the very low *k*_SP,O2_. We further examined this hypothesis with presteady-state experiments in the following section. Lastly, 6-bromoDHHCA (**4**) is figuratively located between DHHCA and 6-cyanoDDHCA. 6-BromoDHHCA (**4**) is large—the largest substrate we tested at 104% of the size of l-DOPA—and it is also easy to oxidize, with an *E*_1/2_ that is 63% of l-DOPA’s. 6-BromoDHHCA is a functional substrate for *Shj*DDO, though less so for LmbB1. Herein lies the third variable of the Goldilocks principle at work in this system: the particular enzyme active site. Looking across the substrates examined, LmbB1 has more effective *k*_SP,O2_ rates for *smaller* substrates, while *Shj*DDO appears to be capable of tolerating the larger 6-bromoDHHCA. This observation is consistent with a *Shj*DDO active site that is more spacious than LmbB1.

To examine the hypothesis of active site volume as a contributing variable to the Goldilocks principle at work in this system, the crystal structure of *S. sclerotialus* l-DOPA 2,3-dioxygenase (i.e., SsDDO, PDB: 6ON3)^38^ was used as a template for homology modeling of LmbB1 and *Shj*DDO enzymes. LmbB1 (accession code: CAA55747) is 53% identical, and *Shj*DDO (accession code: AEY93898) is 50% identical to SsDDO (accession code: WP_051872352). Using Modeller^48,70^ within a UCSF Chimera environment,^50^ two-chain homodimers of LmbB1 and *Shj*DDO were modeled, and then MM2-minimized catechol structures were docked into the active site in three protonation states (Figure S60). Poses were evaluated based on distances of the catecholic oxygens on C3 and C4 to key active site residues and the active site iron (Figures S61–S68). In addition, the boundaries of the active site pocket in each model were visually identified by residue side chain or backbone, and a SurfNet calculation^52^ was performed on the same physical area within each model in order to calculate the relative active site volume for each model. As predicted by our kinetic data, active site volume for the LmbB1 model measures 644 Å^3^ versus the active site volume for *Shj*DDO, which measures 1038 Å^3^. The *Shj*DDO active site is nearly 40% more spacious compared to LmbB1, and examining the sequence alignments reveals that this difference in size is created by the “floor” of the active site (i.e., the region in Figure 4 located below the docked ligand in the orientation provided), which is not conserved. In contrast, the “ceiling” of the active site (i.e., the region above the docked ligand as oriented in Figure 4) is conserved between the two proteins.

**Figure 4:**
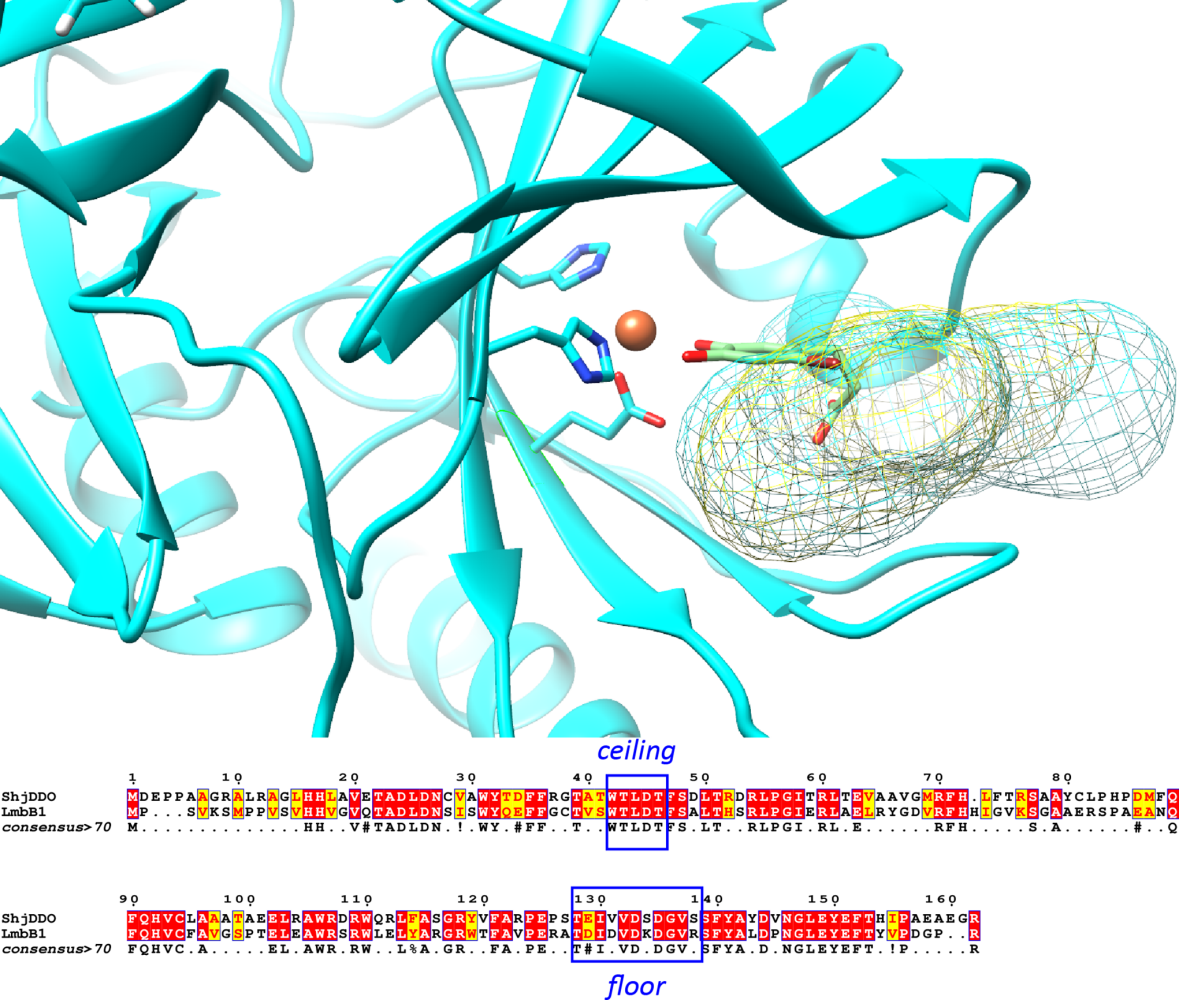
*(upper)* The two-chain homology model of l-DOPA 2,3-dioxygenase from *S. hygroscopicus* *jingganensis* in aqua (*Shj*DDO) built on the template 6ON3 ^38^ with Fe^II^ binding residues and Fe^II^ (bronze) articulated. 6-BromoDHHCA (green) was docked at the active site Fe^II^ using AutoDock Vina^51^ and UCSF Chimera.^50^ The active site volume for *Shj*DDO (blue netting) and LmbB1 (yellow netting) were each calculated using SurfNet^52^ within UCSF Chimera. The relative active site volumes for *Shj*DDO and LmbB1 are superimposed within the *Shj*DDO model. *(lower)* A multiple sequence alignment of *Shj*DDO and LmbB1 using M-Coffee and rendered in ESPript. The regions of sequence used in the SurfNet calculation are boxed in blue and labeled as the “ceiling” and the “floor” of the active site.

Similarly, when docking within equivalent 3-dimensional rectangular prisms positioned in the same way on each protein model, the *Shj*DDO and LmbB1 models produced differently docked poses (Figures S61–S68). For example, when docked in the LmbB1 model, 6-bromoDHHCA (**4**) is not able to approach the active site Fe^II^ as effectively: the catecholic oxygens are bound asymmetrically with an elongated distance between C3-OH and Fe^II^ (Figure S66). These observations contrast with those of l-DOPA (**1**) itself, which docks reasonably well into the LmbB1 model (Figure S68), and 6-bromoDHHCA docked into *Shj*DDO. 6-BromoDHHCA (**4**) can approach Fe^II^ within the *Shj*DDO model with better symmetry and closer distances to essential active site residues (Figure S62). While docking within models should not be overinterpreted, these results are consistent with a less spacious active site for LmbB1, which affords it catalytic advantages on small substrates, and a more spacious active site for *Shj*DDO, for which costs to individual substrate *K*_M_ (Table 3) afford broader substrate tolerance and more effective catalysis on larger substrates. These interpretations are consistent with the larger hypothesis that a tighter active site limits the room that smaller substrates have to sample and likely allows their semiquinone intermediates to more effectively combine with the superoxide radical (Figure 3). The roomier *Shj*DDO active site can accommodate a larger substrate (i.e., 6-bromoDHHCA).

### Presteady-State Kinetics of DHHCA and 6-cyanoDHHCA Derivative with l-DOPA 2,3-Dioxygenase

As described in the previous section, a substrate that is difficult to oxidize should delay the accumulation of the semiquinone intermediate and consequently have a large capacity for inactivating the enzyme. Because of 6-cyanoDHHCA’s spectroscopic properties and the stability of *Shj*DDO at high concentrations, this was an ideal catalytic pair to examine in the presteady state over a single turnover of the reaction pathway.

In modeling the l-DOPA 2,3-dioxygenase reaction with 6-cyanoDHHCA (**5**), the first conclusion was that the strong feature appearing and disappearing within the 2–3 s (Figure 5) was consistent with the transient accumulation of a semiquinone species that precedes Fe-alkylperoxo formation (Figure 3). Fe-alkylperoxo formation breaks the ring aromaticity, and the UV–visible signal at longer wavelengths is lost until ring cleavage of “the lactone” (Figure 3) reestablishes the continuous sp^2^ character and the long-wavelength UV–visible absorbance of the semialdehyde product. The data in Figure 5A were fit to the model shown in Figure 5C in which two charge states of 6-cyanoDHHCA are present in solution (i.e., S_A and S_B) due to the acidic p*K*_a_ value of 7.51 (Table 1). The ratio of the two protonation states of the substrate was calculated from the experimental p*K*_a_ value and reaction pH using the Henderson–Hasselbach equation and fixed in the model. While we allowed the model to propagate either protonation state to the ES complex, the software consistently drove one path to zero, which implies that only one protonation state is competent to form the ES complex.^65,71^ This was also the case when we modeled the enzymatic reaction at pH 7.25 (Figure S58). After ES complex formation, oxygen binds rapidly and irreversibly,^72,73^ followed by semiquinone formation. The steps that form the semiquinone and other intermediates were also fixed as irreversible, which is consistent with the thermodynamics of the oxidative ring cleavage.^66^ After the semiquinone species (i.e., ESQ) forms, it decays to a spectroscopically silent EI that is consistent with the Fe-alkylperoxo and all subsequent intermediates that precede lactone cleavage and semialdehyde formation. The UV-absorption returns between 10 and 20 s with the formation of the ring-cleaved semialdehyde product (EP) that is released from the enzyme (P). Because it was independently determined (Figure S52), the extinction coefficient of the 6-cyanoDHHCA cleavage product at 380 nm (i.e., P) was fixed during fitting, which permitted an estimate of the extinction coefficient of the 6-cyanoDHHCA semiquinone species (i.e., ESQ) of 38 950 ± 9250 M^–1^cm^–1^. The product semialdehyde of 6-cyanoDHHCA ring cleavage cannot cyclize as the product of l-DOPA cleavage can,^74^ but in the steady state, we observed slow loss of the UV–visible signal that was modeled in Figure 5C as an equilibrium of P with the spectroscopically silent P_X. It is possible that the hydroxymuconic semialdehyde chain is in equilibrium with cyclic hemiacetal species. Indeed, at pH 7.25, the equilibrium of P to P_X shifts to favor P (Figure S58). Lastly, a close examination of the first ∼0.8 s of the 6-cyanoDHHCA (**5**) data trace revealed several low-intensity spectroscopic features that were excluded from the fit.

**Figure 5:**
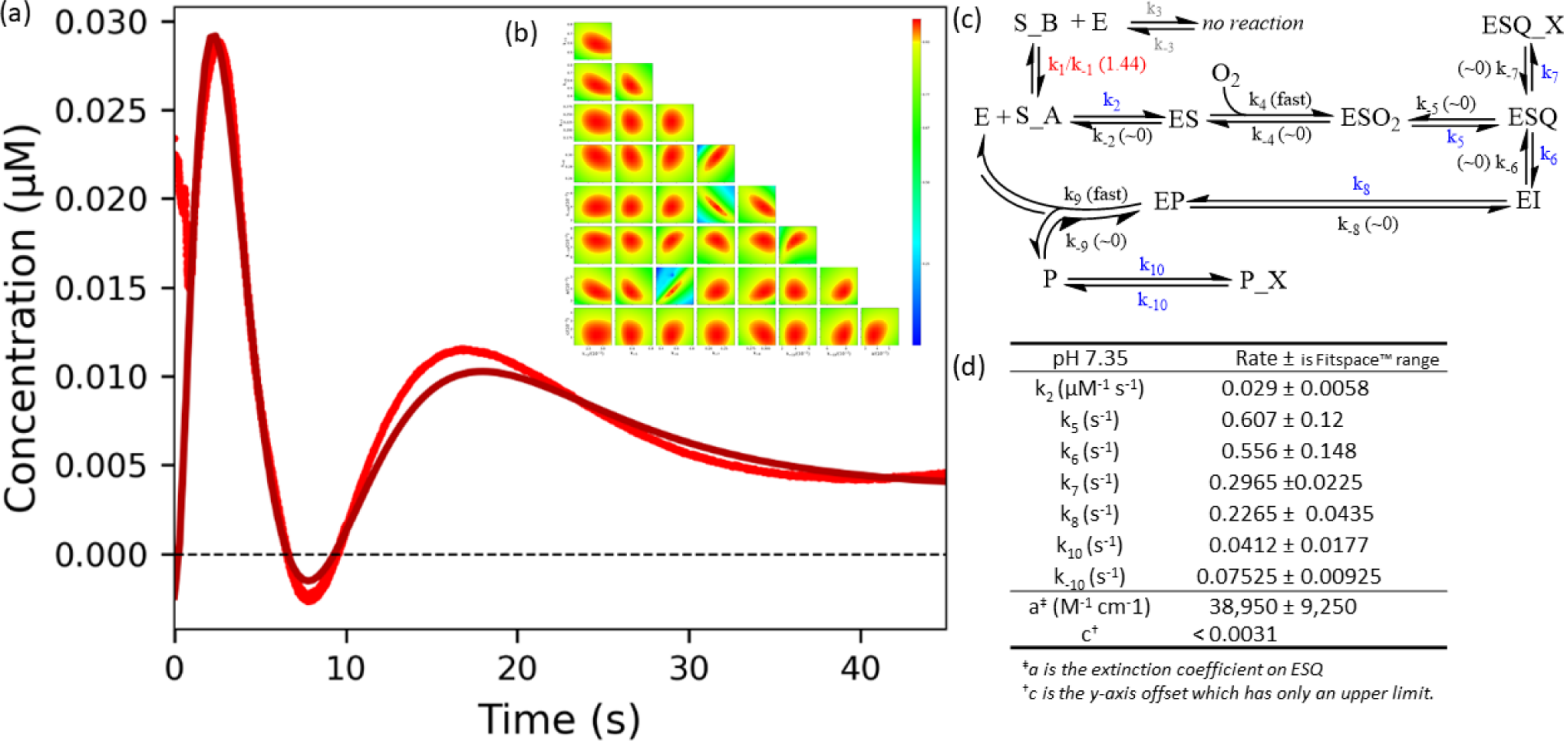
Presteady state analysis of the 6-cyanoDHHCA (**5**) reaction with *S. hygroscopicus* *jingganensis* l-DOPA 2,3-dioxygenase (*Shj*DDO). (**a**) 6-CyanoDHHCA (5.6 μM) *Shj*DDO (56 μM) at pH 7.35, 14 °C, ∼1000 μM O_2_, and 380 nm produces two dominant spectroscopic features between 2 and 3 s and a slower feature that accumulates between 10 and 20 s consistent with transient accumulation of a semiquinone species followed by the ring cleavage product. The first 800 ms contains additional low-intensity spectroscopic features that were excluded from the fit. From 0.8 to 45 s, the data (light red) are fit to the model (dark red) shown in (**c**) and the fitted parameters are shown in (d) with the calculated FitSpace range. (**c**) A reaction pathway consistent with (a) and the data presented herein, which is described in the methods. (d) Fitted parameters for (a) according to the model in (c), the value shown is the center of the FitSpace range, with the magnitude of the range expressed as ±values from the center. (**b**) FitSpace confidence contour analysis^54^ for (a), based on the model in (c). Larger versions of FitSpace contour plots and Chi^2^ plots are available in Figure S58.

The second readily apparent conclusion was that the presteady state data were most persuasively modeled only when an inactivation step was included, in which ESQ forms the nonproductive and spectroscopically silent ESQ_X. We modeled this inactivation step as irreversible, which is consistent with what we have observed in the steady state for similar substrates^39^ and consistent with the unproductive quenching of the semiquinone and loss of activated oxygen from the catalytic complex. When 6-cyanoDHHCA reacts with *Shj*DDO, the productive formation of EI (*k*_6_ ∼ 0.55 s^–1^) is only 1.85 times faster than the rate of inactivation (*k*_7_ ∼ 0.3 s^–1^), which produces a significant loss of the ESQ feature observed in Figure 5A. This interpretation is supported by the model of DHHCA reacting in the presteady state with *Shj*DDO (Figure S59), in which the productive formation of EI (*k*_6_) is ∼40 times faster than the rate of inactivation (*k*_7_). Taken together, the presteady state data support the interpretation offered in the previous section. Difficult-to-oxidize substrates such as 6-cyanoDHHCA (**5**) result in significant enzyme inactivation, while smaller, readily oxidizable substrates are less prone to inactivation.

## Conclusion

Extradiol dioxygenase chemistry is well-known in the degradation of natural and man-made sources of aromatic carbon; however, biosynthetic dioxygenases such as l-DOPA 2,3-dioxygenase fit less readily into this well-characterized role. In order to understand if l-DOPA 2,3-dioxgenases also demonstrated a capacity or even a proclivity to cleave lignin-derived aromatic carbon, we chose to study the l-DOPA 2,3-dioxygenase reaction using the scaffold of 3,4-dihydroxyhydrocinnamic acid (DHHCA), also known as hydrocaffeic acid, a catecholic example of lignin-derived carbon^40^ that is not only similar in structure to the preferred substrate of l-DOPA 2,3-dioxygenase, l-3,4-dihydroxyphenylalanine (l-DOPA), but also amenable to synthetic functionalization of the aromatic ring.

The synthesis of DHHCA analogues (Figure 2) 3-(2-bromo-4,5-dihydroxyphenyl) propanoic acid (6-bromoDHHCA, **4**), 3-(2-cyano-4,5-dihydroxyphenyl) propanoic acid (6-cyanoDHHCA, **5**), and 3-(4,5-dihydroxy-2-nitrophenyl) propanoic acid (6-nitroDHHCA, **6**) reported herein are the first of their kind, and evaluation of their physical properties and activity as enzymatic substrates on two l-DOPA 2,3-dioxygenases from *S. lincolnensis* and *S. hygroscopicus* *jingganensis* has yielded promising mechanistic insights. DHHCA scaffolds are indeed substrates of l-DOPA 2,3-dioxygenase. 6-Substituted derivatives such as 6-bromoDHHCA (**4**) reacted with oxygen inside the l-DOPA 2,3-dioxygenase from *S. hygroscopicus* *jingganensis* (*Shj*DDO) with a second-order rate constant that was ∼11% of the enzymatic activity on l-DOPA. The more spacious active site of *Shj*DDO likely allowed this level of activity, while the smaller volume of the LmbB1 active site limited activity. Termination of the reaction pathway due to unproductive reaction with oxygen defines the presteady state turnover of substrates, such as 6-cyanoDHHCA (**5**), which are defined by their acidic p*K*_a_ values and high redox potential. Taken together, these results argue for an integrated understanding of substrate size, redox potential, and active-site capacity to understand substrate–enzyme compatibility and provide actionable insights to fuel the pursuit of dioxygenase catalysts that robustly cleave 6-X-DHHCA and other synthetically modified lignin-derived catecholic monomers as starting points for functionalized semialdehydes.

## Supplementary Material



## Notes

Detailed information include supplemental data (Figure S1), a Hammett plot (Figure S2) of synthetic compounds, NMR spectra of synthetic compounds, p*K*_a_ spectra, HPLC chromatograms, cyclic voltammagrams for synthetic compounds (Figures S3–S51), extinction coefficients for steady-state cleavage products (Figure S52), Michaelis–Menten plots of DHHCA/6-X-DHHCA substrates (Figures S53–S56), presteady state analysis of *Shj*DDO with DHHCA and 6-cyanoDHHCA (Figures S57–S59), small molecule docking of DHHCA/6-X-DHHCAs in homology models of *Shj*DDO and LmbB1 (Figures S60–S68), and a plot of redox potential vs p*K*_a_for catecholic substrates (Figure S69) (PDF)

## References

[1] Kamimura N., Takahashi K., Mori K., Araki T., Fujita M., Higuchi Y., Masai E. (2017). Bacterial Catabolism of Lignin-Derived
Aromatics: New Findings in a Recent Decade: Update on Bacterial Lignin
Catabolism. Environ. Microbiol. Rep..

[2] Labeeuw L., Martone P. T., Boucher Y., Case R. J. (2015). Ancient Origin of
the Biosynthesis of Lignin Precursors. Biol.
Direct..

[3] Wu W., Dutta T., Varman A. M., Eudes A., Manalansan B., Loqué D., Singh S. (2017). Lignin Valorization: Two Hybrid Biochemical
Routes for the Conversion of Polymeric Lignin into Value-Added Chemicals. Sci. Rep..

[4] Vaillancourt F. H., Bolin J. T., Eltis L. D. (2006). The Ins and Outs
of Ring-Cleaving
Dioxygenases. Crit. Rev. Biochem. Mol. Biol..

[5] Zhang R., Li C., Wang J., Yan Y. (2019). Microbial Ligninolysis: Toward a
Bottom-Up Approach for Lignin Upgrading. Biochemistry.

[6] Sun J., Ren Y., Raza M., Sun X., Yuan Q. (2018). Microbial Production
of Glutaconic Acid via Extradiol Ring Cleavage of Catechol. J. Chem. Technol. Biotechnol..

[7] Johnson C. W., Beckham G. T. (2015). Aromatic Catabolic
Pathway Selection for Optimal Production
of Pyruvate and Lactate from Lignin. Metab.
Eng..

[8] Bai X., Nie M., Diwu Z., Wang L., Nie H., Wang Y., Zhang B., Yin Q. (2021). Preparation of 2-Hydroxymuconic Semialdehyde
from Catechol by Combining Enzymatic Catalysis with Bisulfite Nucleophilic
Addition. J. Environ. Chem. Eng..

[9] Sun X., Lin Y., Huang Q., Yuan Q., Yan Y. (2013). A Novel Muconic Acid
Biosynthesis Approach by Shunting Tryptophan Biosynthesis via Anthranilate. Appl. Environ. Microbiol..

[10] Xie N.-Z., Liang H., Huang R.-B., Xu P. (2014). Biotechnological Production
of Muconic Acid: Current Status and Future Prospects. Biotechnol. Adv..

[11] Colabroy K. L., Begley T. P. (2005). Tryptophan Catabolism:
Identification and Characterization
of a New Degradative Pathway. J. Bacteriol..

[12] Kurnasov O., Goral V., Colabroy K., Gerdes S., Anantha S., Osterman A., Begley T. P. (2003). NAD Biosynthesis: Identification
of the Tryptophan to Quinolinate Pathway in Bacteria. Chem. Biol..

[13] Christinet L., Burdet F. X., Zaiko M., Hinz U., Zryd J.-P. (2004). Characterization
and Functional Identification of a Novel Plant 4,5-Extradiol Dioxygenase
Involved in Betalain Pigment Biosynthesis in Portulaca Grandiflora. Plant Physiol..

[14] Girod P. A., Zryd J. P. (1991). Biogenesis of Betalains:
Purification and Partial Characterization
of DOPA 4,5-Dioxygenase from Amanita Muscaria. Phytochemistry.

[15] Hinz U. G., Fivaz J., Girod P. A., Zyrd J. P. (1997). The Gene
Coding
for the DOPA Dioxygenase Involved in Betalain Biosynthesis in Amanita
Muscaria and Its Regulation. Mol. Gen. Genet..

[16] Gómez-Álvarez H., Iturbe P., Rivero-Buceta V., Mines P., Bugg T. D. H., Nogales J., Díaz E. (2022). Bioconversion of Lignin-Derived Aromatics
into the Building Block Pyridine 2,4-Dicarboxylic Acid by Engineering
Recombinant *Pseudomonas Putida* Strains. Bioresour. Technol..

[17] Guan A.-Y., Liu C.-L., Sun X.-F., Xie Y., Wang M.-A. (2016). Discovery
of Pyridine-Based Agrochemicals by Using Intermediate Derivatization
Methods. Bioorg. Med. Chem..

[18] Ling Y., Hao Z.-Y., Liang D., Zhang C.-L., Liu Y.-F., Wang Y. (2021). The Expanding Role
of Pyridine and Dihydropyridine Scaffolds in Drug
Design. Drug Des. Devel. Ther..

[19] Fortin P. D., Lo A. T.-F., Haro M.-A., Kaschabek S. R., Reineke W., Eltis L. D. (2005). Evolutionarily Divergent
Extradiol
Dioxygenases Possess Higher Specificities for Polychlorinated Biphenyl
Metabolites. J. Bacteriol..

[20] Sainsbury P. D., Hardiman E. M., Ahmad M., Otani H., Seghezzi N., Eltis L. D., Bugg T. D. H. (2013). Breaking
down Lignin to High-Value
Chemicals: The Conversion of Lignocellulose to Vanillin in a Gene
Deletion Mutant of Rhodococcus Jostii RHA1. ACS Chem. Biol..

[21] Mycroft Z., Gomis M., Mines P., Law P., Bugg T. D. H. (2015). Biocatalytic
Conversion of Lignin to Aromatic Dicarboxylic Acids in Rhodococcus
Jostii RHA1 by Re-Routing Aromatic Degradation Pathways. Green Chem..

[22] Salvachúa D., Werner A. Z., Pardo I., Michalska M., Black B. A., Donohoe B. S., Haugen S. J., Katahira R., Notonier S., Ramirez K. J., Amore A., Purvine S. O., Zink E. M., Abraham P. E., Giannone R. J., Poudel S., Laible P. D., Hettich R. L., Beckham G. T. (2020). Outer Membrane
Vesicles
Catabolize Lignin-Derived Aromatic Compounds in Pseudomonas Putida
KT2440. Proc. Natl. Acad. Sci. U. S. A..

[23] Táncsics A., Szabó I., Baka E., Szoboszlay S., Kukolya J., Kriszt B., Márialigeti K. (2010). Investigation
of Catechol 2,3-Dioxygenase and 16S rRNA Gene Diversity in Hypoxic,
Petroleum Hydrocarbon Contaminated Groundwater. Syst. Appl. Microbiol..

[24] Wu H., Du X., Wu W., Zheng J., Song J., Xie J. (2023). Metagenomic
Analysis Reveals Specific BTEX Degrading Microorganisms of a Bacterial
Consortium. AMB Express.

[25] Bugg T. D. H., Ahmad M., Hardiman E. M., Singh R. (2011). The Emerging Role for
Bacteria in Lignin Degradation and Bio-Product Formation. Curr. Opin. Biotechnol..

[26] Leigh M. B., Prouzová P., Macková M., Macek T., Nagle D. P., Fletcher J. S. (2006). Polychlorinated
Biphenyl (PCB)-Degrading Bacteria Associated
with Trees in a PCB-Contaminated Site. Appl.
Environ. Microbiol..

[27] Morya R., Kumar M., Singh S. S., Thakur I. S. (2019). Genomic Analysis
of Burkholderia Sp. ISTR5 for Biofunneling of Lignin-Derived Compounds. Biotechnol. Biofuels.

[28] Kovaleva E. G., Lipscomb J. D. (2007). Crystal Structures
of Fe2+ Dioxygenase Superoxo, Alkylperoxo,
and Bound Product Intermediates. Science.

[29] Spence E. L., Kawamukai M., Sanvoisin J., Braven H., Bugg T. D. (1996). Catechol
Dioxygenases from Escherichia Coli (MhpB) and Alcaligenes Eutrophus
(MpcI): Sequence Analysis and Biochemical Properties of a Third Family
of Extradiol Dioxygenases. J. Bacteriol..

[30] Han S., Eltis L. D., Timmis K. N., Muchmore S. W., Bolin J. T. (1995). Crystal
Structure of the Biphenyl-Cleaving Extradiol Dioxygenase from a PCB-Degrading
Pseudomonad. Science.

[31] Peschke U., Schmidt H., Zhang H.-Z., Piepersberg W. (1995). Molecular
Characterization of the Lincomycin-production Gene Cluster of Streptomyces
Lincolnensis 78–11. Mol. Microbiol..

[32] Neusser D., Schmidt H., Spizèk J., Novotnà J., Peschke U., Kaschabeck S., Tichy P., Piepersberg W. (1998). The Genes
lmbB1 and lmbB2 of Streptomyces Lincolnensis Encode Enzymes Involved
in the Conversion of L-Tyrosine to Propylproline during the Biosynthesis
of the Antibiotic Lincomycin A. Arch. Microbiol..

[33] Najmanova L., Ulanova D., Jelinkova M., Kamenik Z., Kettnerova E., Koberska M., Gazak R., Radojevic B., Janata J. (2014). Sequence Analysis of Porothramycin Biosynthetic Gene
Cluster. Folia Microbiol..

[34] Hu Y., Phelan V. V., Farnet C. M., Zazopoulos E., Bachmann B. O. (2008). Reassembly of Anthramycin Biosynthetic
Gene Cluster
by Using Recombinogenic Cassettes. ChemBiochem.

[35] Li W., Khullar A., Chou S., Sacramo A., Gerratana B. (2009). Biosynthesis
of Sibiromycin, a Potent Antitumor Antibiotic. Appl. Environ. Microbiol..

[36] Li W., Chou S., Khullar A., Gerratana B. (2009). Cloning and
Characterization of the Biosynthetic Gene Cluster for Tomaymycin,
an SJG-136 Monomeric Analog. Appl. Environ.
Microbiol..

[37] Höfer I., Crüsemann M., Radzom M., Geers B., Flachshaar D., Cai X., Zeeck A., Piel J. (2011). Insights into the Biosynthesis of
Hormaomycin, An Exceptionally Complex Bacterial Signaling Metabolite. Chem. Biol..

[38] Wang Y., Shin I., Fu Y., Colabroy K. L., Liu A. (2019). Crystal Structures
of L-DOPA Dioxygenase from Streptomyces Sclerotialus. Biochemistry.

[39] Ringenbach S., Yoza R., Jones P. A., Du M., Klugh K. L., Peterson L. W., Colabroy K. (2024). Discovery and Characterization
of
L-Dopa 2,3-Dioxygenase from Streptomyces Hygroscopicus Jingganensis. Arch. Biochem. Biophys..

[40] Phelps C. D., Young L. Y. (1997). Microbial Metabolism
of the Plant Phenolic Compounds
Ferulic and Syringic Acids under Three Anaerobic Conditions. Microb. Ecol..

[41] Malarczyk E., Rogalski J., Leonowicz A. (1994). Transformation
of Ferulic Acid by
Soil Bacteria Nocardia Provides Various Valuable Phenolic Compounds. Acta Biotechnol..

[42] Goldberg A. M., Robinson M. K., Starr E. S., Marasco R. N., Alana A. C., Cochrane C. S., Klugh K. L., Strzeminski D. J., Du M., Colabroy K. L., Peterson L. W. (2021). L-DOPA
Dioxygenase Activity on 6-Substituted
Dopamine Analogues. Biochemistry.

[43] Sambrook, J. ; Fritsch, E. F. ; Maniatis, T. Molecular Cloning, A Laboratory Manual; Cold Spring Harbor Laboratory Press, 1989; Vol. 3.

[44] Gill S. C., von Hippel P. H. (1989). Calculation
of Protein Extinction Coefficients from
Amino Acid Sequence Data. Anal. Biochem..

[45] Colabroy K. L., Horwitz A. D., Basciano V. R., Fu Y., Travitz K. M., Robinson M. K., Shimanski B. A., Hoffmann T. W. (2019). A New Way of Belonging:
Active-Site Investigation of L-DOPA Dioxygenase, a VOC Family Enzyme
from Lincomycin Biosynthesis. Biochemistry.

[46] Johnson K. A. (2019). New Standards
for Collecting and Fitting Steady State Kinetic Data. Beilstein J. Org. Chem..

[47] Gibson Q. H., Swoboda B. E., Massey V. (1964). Kinetics and
Mechanism of Action
of Glucose Oxidase. J. Biol. Chem..

[48] Eswar, N. ; Webb, B. ; Marti-Renom, M. A. ; Madhusudhan, M. S. ; Eramian, D. ; Shen, M.-Y. ; Pieper, U. ; Sali, A. Comparative Protein Structure Modeling Using MODELLER. In Current Protocols in Bioinformatics; John Wiley & Sons, Inc.: 2006. 10.1002/0471250953.bi0506s15 PMC4186674 18428767

[49] Wang J., Wang W., Kollman P. A., Case D. A. (2006). Automatic Atom Type
and Bond Type Perception in Molecular Mechanical Calculations. J. Mol. Graphics Modell..

[50] Huang, C. C. ; Couch, G. S. ; Pettersen, E. F. ; Ferrin, T. E. Chimera: An Extensible Molecular Modeling Application Constructed Using Standard Components; ScienceOpen, Inc., 1996.

[51] Trott O., Olson A. J. (2010). AutoDock Vina: Improving
the Speed and Accuracy of
Docking with a New Scoring Function, Efficient Optimization, and Multithreading. J. Comput. Chem..

[52] Laskowski R. A. (1995). SURFNET:
A Program for Visualizing Molecular Surfaces, Cavities, and Intermolecular
Interactions. J. Mol. Graphics.

[53] Johnson K. A. (2009). Fitting
Enzyme Kinetic Data with KinTek Global Kinetic Explorer. Methods Enzymol..

[54] Johnson K. A., Simpson Z. B., Blom T. (2009). FitSpace Explorer:
An Algorithm to
Evaluate Multidimensional Parameter Space in Fitting Kinetic Data. Anal. Biochem..

[55] Rote J. C., Malkowski S. N., Cochrane C. S., Bailey G. E., Brown N. S., Cafiero M., Peterson L. W. (2017). Catechol Reactivity: Synthesis of
Dopamine Derivatives Substituted at the 6-Position. Synth. Commun..

[56] Davidson S. J., Pilkington L. I., Dempsey-Hibbert N. C., El-Mohtadi M., Tang S., Wainwright T., Whitehead K. A., Barker D. (2018). Modular Synthesis and Biological
Investigation of 5-Hydroxymethyl
Dibenzyl Butyrolactones and Related Lignans. Molecules.

[57] Miller M. A., Lipscomb J. D. (1996). Homoprotocatechuate 2,3-Dioxygenase from Brevibacterium
Fuscum. A Dioxygenase with Catalase Activity. J. Biol. Chem..

[58] Lipscomb J. D. (2008). Mechanism
of Extradiol Aromatic Ring-Cleaving Dioxygenases. Curr. Opin. Struct. Biol..

[59] Wang Y., Liu K. F., Yang Y., Davis I., Liu A. (2020). Observing
3-Hydroxyanthranilate-3,4-Dioxygenase in Action through a Crystalline
Lens. Proc. Natl. Acad. Sci. U. S. A..

[60] Jodko-Piórecka K., Sikora B., Kluzek M., Przybylski P., Litwinienko G. (2022). Antiradical Activity of Dopamine, L-DOPA, Adrenaline,
and Noradrenaline in Water/Methanol and in Liposomal Systems. J. Org. Chem..

[61] Capitain C., Wagner S., Hummel J., Tippkötter N. (2021). Investigation
of C–N Formation Between Catechols and Chitosan for the Formation
of a Strong, Novel Adhesive Mimicking Mussel Adhesion. Waste Biomass Valorization.

[62] Hansch C., Leo A., Taft R. W. (1991). A Survey
of Hammett Substituent Constants and Resonance
and Field Parameters. Chem. Rev..

[63] Jo D.-H., Chiou Y.-M., Que L. (2001). Models for
Extradiol Cleaving Catechol
Dioxygenases: Syntheses, Structures, and Reactivities of Iron (II)-Monoanionic
Catecholate Complexes. Inorg. Chem..

[64] Shu L., Chiou Y.-M., Orville A. M., Miller M. A., Lipscomb J. D., Que L. (1995). X-Ray Absorption Spectroscopic Studies
of the Fe (II) Active Site of Catechol 2, 3-Dioxygenase. Implications
for the Extradiol Cleavage Mechanism. Biochemistry.

[65] Vaillancourt F. H., Barbosa C. J., Spiro T. G., Bolin J. T., Blades M. W., Turner R. F. B., Eltis L. D. (2002). Definitive Evidence for Monoanionic
Binding of 2,3-Dihydroxybiphenyl to 2,3-Dihydroxybiphenyl 1,2-Dioxygenase
from UV Resonance Raman Spectroscopy, UV/Vis Absorption Spectroscopy,
and Crystallography. J. Am. Chem. Soc..

[66] Kovaleva E.
G., Neibergall M. B., Chakrabarty S., Lipscomb J. D. (2007). Finding Intermediates
in the O2 Activation Pathways of Non-Heme Iron Oxygenases. Acc. Chem. Res..

[67] Mbughuni M. M., Meier K. K., Münck E., Lipscomb J. D. (2012). Substrate-Mediated
Oxygen Activation by Homoprotocatechuate 2,3-Dioxygenase: Intermediates
Formed by a Tyrosine 257 Variant. Biochemistry.

[68] Kovaleva E.
G., Lipscomb J. D. (2012). Structural
Basis for the Role of Tyrosine 257 of Homoprotocatechuate
2,3-Dioxygenase in Substrate and Oxygen Activation. Biochemistry.

[69] Matysek A., Tomaszczyk J. (2022). In Quest of
Goldilocks Ranges in Searching for Information
on the Web. J. Doc..

[70] Sali A., Blundell T. L. (1993). Comparative Protein
Modelling by Satisfaction of Spatial
Restraints. J. Mol. Biol..

[71] Ishida T., Senda T., Tanaka H., Yamamoto A., Horiike K. (2005). Single-Turnover
Kinetics of 2,3-Dihydroxybiphenyl 1,2-Dioxygenase Reacting with 3-Formylcatechol. Biochem. Biophys. Res. Commun..

[72] Groce S. L., Miller-Rodeberg M. A., Lipscomb J. D. (2004). Single-Turnover Kinetics of Homoprotocatechuate
2,3-Dioxygenase†. Biochemistry.

[73] Groce S. L., Lipscomb J. D. (2005). Aromatic Ring Cleavage
by Homoprotocatechuate 2,3-Dioxygenase:
Role of His200 in the Kinetics of Interconversion of Reaction Cycle
Intermediates†. Biochemistry.

[74] Colabroy K.
L., Hackett W. T., Markham A. J., Rosenberg J., Cohen D. E., Jacobson A. (2008). Biochemical
Characterization of L-DOPA
2,3-Dioxygenase, a Single-Domain Type I Extradiol Dioxygenase from
Lincomycin Biosynthesis. Arch. Biochem. Biophys..

