# Supplementary material for: Probing the Mechanism of l‑DOPA 2,3-Dioxygenase Using Synthetic Derivatives of 3,4-Dihydroxyhydrocinnamic Acid
Source: ACS Omega. 2025 Jul 16;10(29):32053–69. doi: 10.1021/acsomega.5c03691 (PMC12311729; doi:10.1021/acsomega.5c03691)
Supplement: Supplementary file 1 [file ao5c03691_si_001.pdf]

SUPPLEMENTAL INFORMATION
Probing L-DOPA 2,3-dioxygenase mechanism with synthetic derivatives of 3,4-dihydroxyhydrocinnamic acid

Amen Taye Demisew[a], Jon R. Cohen[a], Emma G. Gruss[b], Jennifer D. Bui[b], Jessica L. Steiner [b], Gisela Xhafkollari[b], Ryan N. Marasco[b], Mark Betonio[b], David Strzeminski[a], Sebastian Leyes Porello[a], Keri L. Colabroy*[a], Larryn W. Peterson*[b]

[a]Department of Chemistry Muhlenberg College, Allentown, PA 18104, [b]Department of Chemistry, Rhodes College, Memphis, TN 38112,

Author information

*Corresponding authors

Muhlenberg College, 2400 Chew St, Allentown, PA 18104
Email: kericolabroy@muhlenberg.edu
Rhodes College, 2000 North Parkway, Memphis, TN 38112
Email: petersonl@rhodes.edu

Table of Contents

Supplemental figures, S1 and S2	2
NMR spectra	3
HPLC chromatograms	12
pK_a spectra	14
Cyclic voltammograms	22
Extinction coefficients for steady-state cleavage products	28
Michaelis-Menten Plots of DHHCA/6-x-DHHCA substrates	29
Pre-steady state analysis of ShjDDO with DHHCA and 6-cyanoDHHCA	32
Small molecule docking	38
Redox potential and pK_a values for catecholic substrates	49
References	49

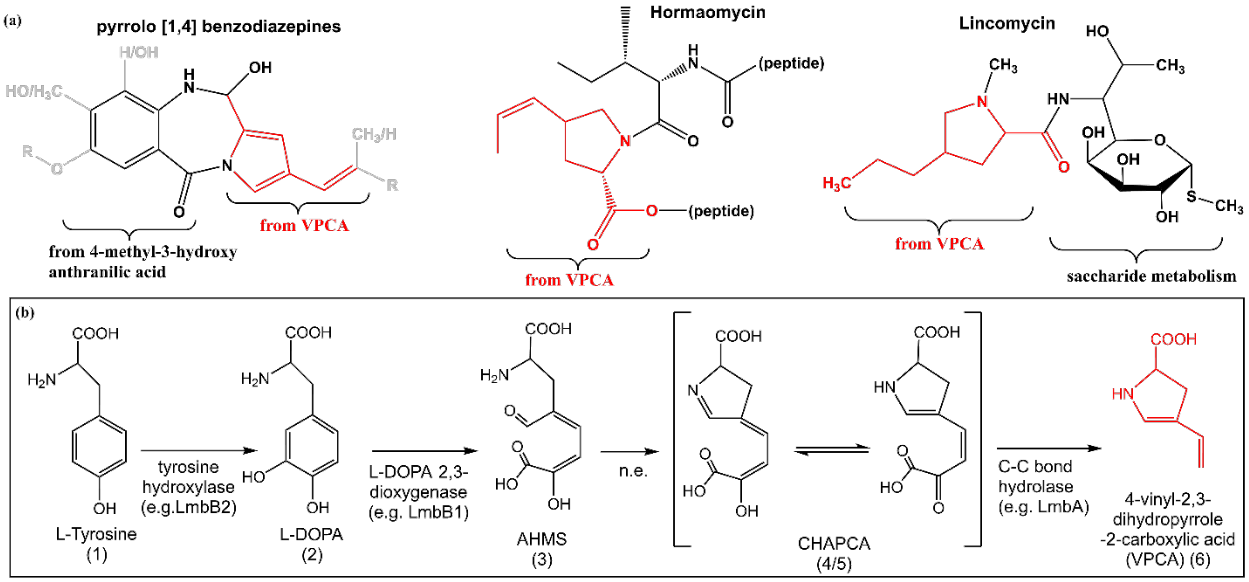

Figure S1. (a) The biosynthetic mini-pathway to VPCA is comprised of a minimum of three steps. (b) the VPCA synthon is elaborated and embedded within different natural product structures.

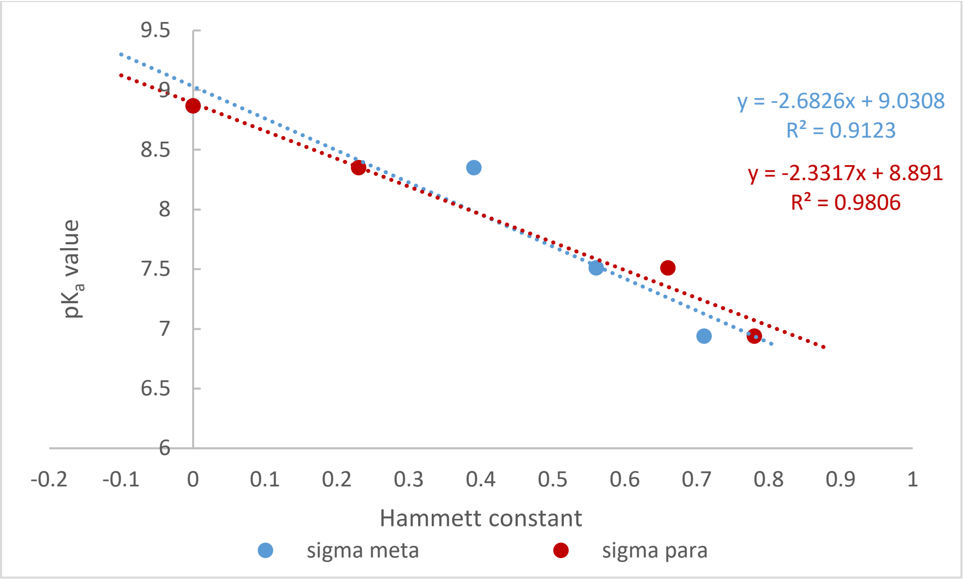

Figure S2. Hammett Plot of experimentally determined pK_a values of DHHCA and synthetic derivatives.

NMR Spectra

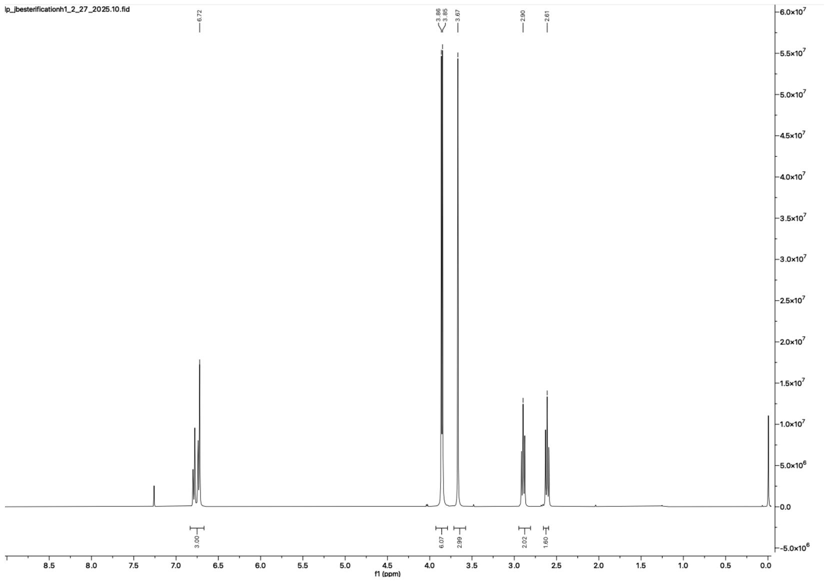

Figure S3. ^{1}H NMR spectrum of methyl 3-(3,4-dimethoxyphenyl)propanoate (**7**). ^{1}H NMR (400 MHz, $CDCl_3$) δ 6.83 – 6.67 (m, 3H), 3.86 (2x s, 6H), 3.67 (s, 3H), 2.90 (t, J = 7.8 Hz, 2H), 2.61 (t, J = 7.8 Hz, 2H).

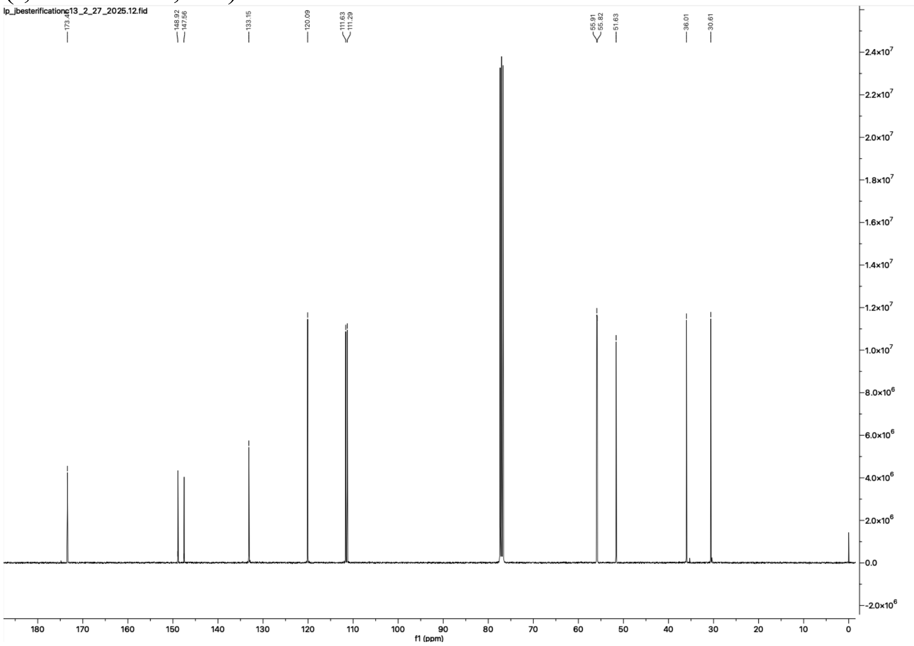

Figure S4. ^{13}C NMR spectrum of methyl 3-(3,4-dimethoxyphenyl)propanoate (**7**). ^{13}C NMR (101 MHz, $CDCl_3$) δ 173.41, 148.92, 147.56, 133.15, 120.09, 111.63, 111.29, 55.91, 55.82, 51.63, 36.01, 30.61.

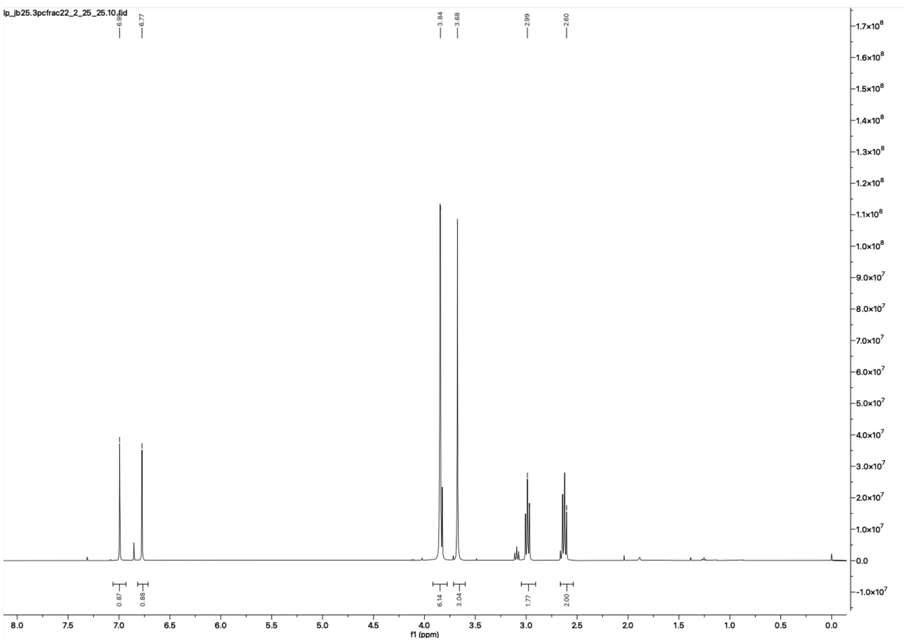

Figure S5. ^{1}H NMR spectrum for methyl 3-(2-bromo-4,5-dimethoxyphenyl) propanoate (**8**). ^{1}H NMR (400 MHz, $CDCl_3$) δ 6.99 (s, 1H), 6.77 (s, 1H), 3.85 (2x s, 6H), 3.68 (s, 3H), 2.99 (t, *J* = 7.8 Hz, 2H), 2.62 (t, *J* = 7.8 Hz, 2H).

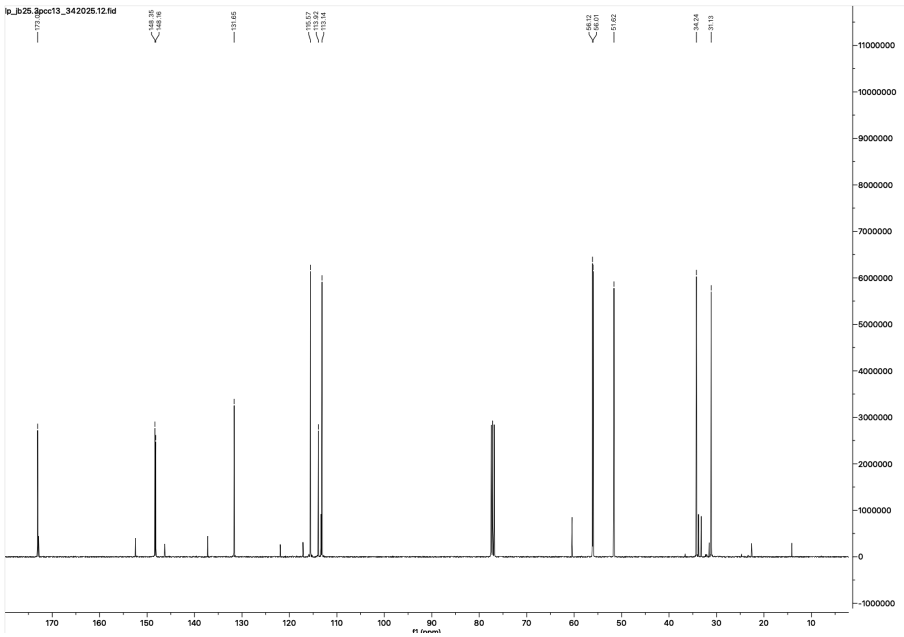

Figure S6. ^{13}C NMR spectrum for methyl 3-(2-bromo-4,5-dimethoxyphenyl) propanoate **(8).** ^{13}C NMR (101 MHz, $CDCl_3$) δ 173.07, 148.35, 148.16, 131.65, 115.57, 113.92, 113.14, 56.12, 56.01, 51.62, 34.24, 31.13.

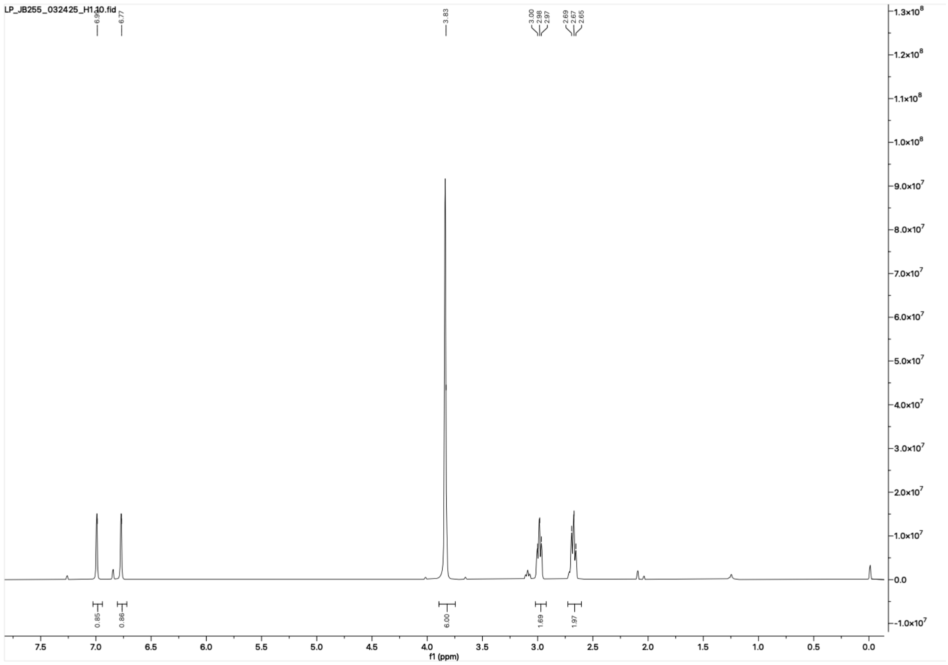

Figure S7. ^{1}H NMR spectrum for 3-(2-bromo-4,5-dimethoxyphenyl) propanoic acid **(9)**. ^{1}H NMR (400 MHz, $CDCl_3$) δ 6.99 (s, 1H), 6.77 (s, 1H), 3.83 (s, 6H), 2.98 (t, *J* = 6.8 Hz, 2H), 2.67 (t, *J* = 7.7 Hz, 2H).

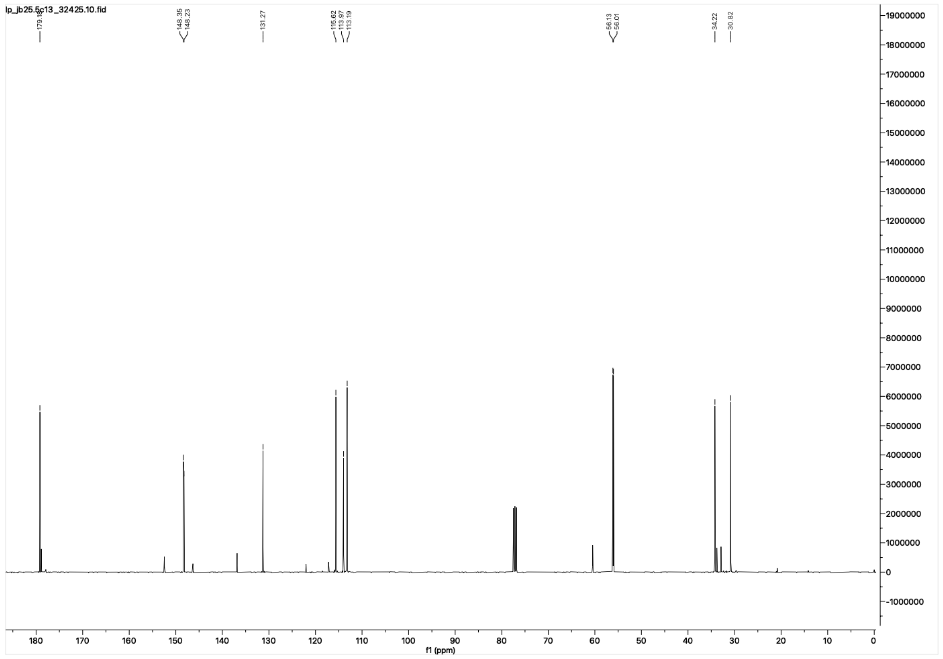

Figure S8. ^{13}C NMR spectrum for 3-(2-bromo-4,5-dimethoxyphenyl) propanoic acid **(9)**. ^{13}C NMR (101 MHz, $CDCl_3$) δ 179.18, 148.35, 148.23, 131.27, 115.62, 113.97, 113.19, 56.13, 56.01, 34.22, 30.82.

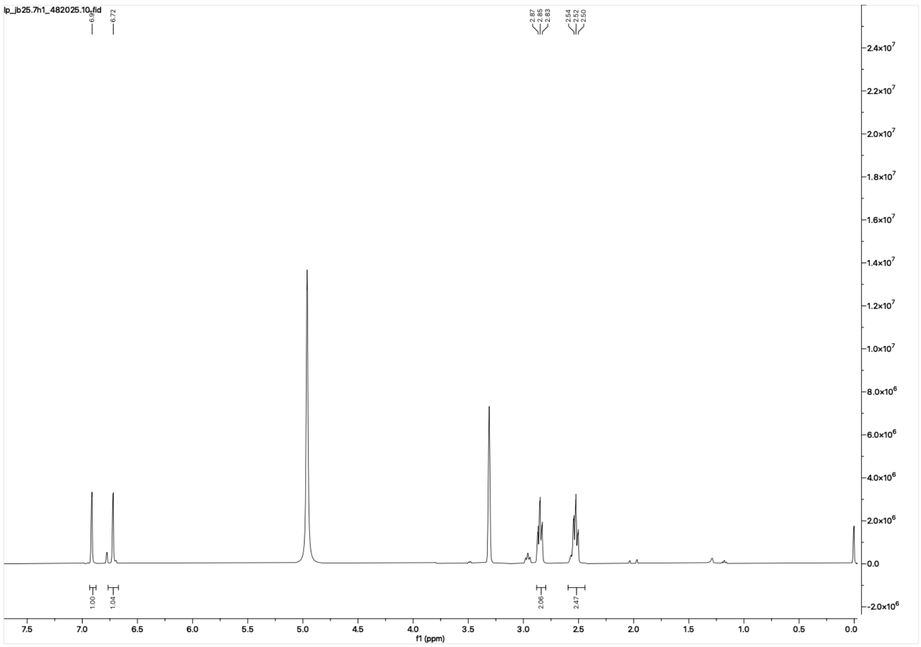

Figure S9. ^{1}H NMR spectrum for 3-(2-bromo-4,5-dihydroxyphenyl) propanoic acid **(4)**. ^{1}H NMR (400 MHz, CD_3OD) δ 6.91 (s, 1H), 6.72 (s, 1H), 2.85 (t, *J* = 7.8 Hz, 2H), 2.52 (t, *J* = 7.8 Hz, 2H).

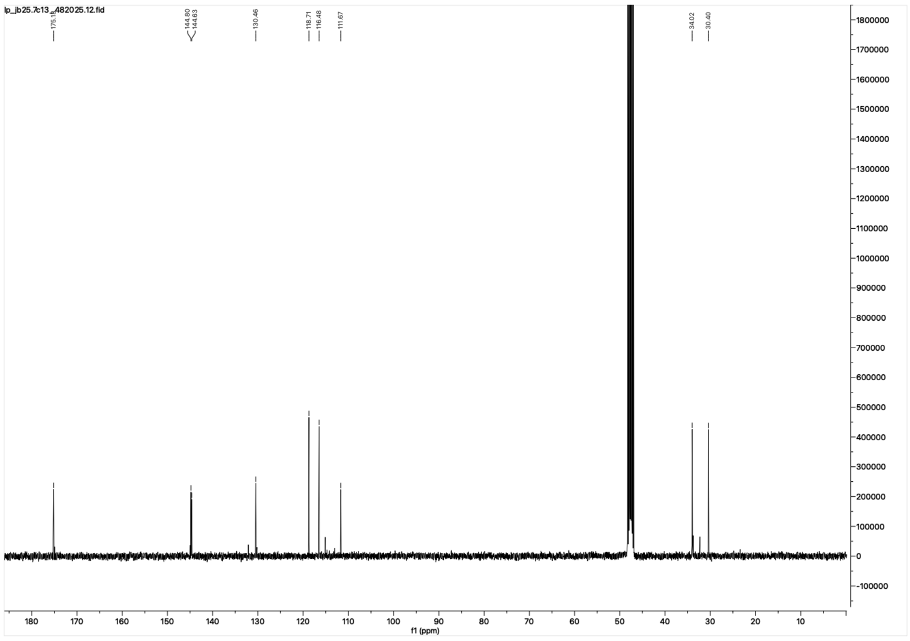

Figure S10. ^{13}C NMR spectrum for 3-(2-bromo-4,5-dihydroxyphenyl) propanoic acid **(4)**. ^{13}C NMR (101 MHz, CD_3OD) δ 175.13, 144.80, 144.63, 130.46, 118.71, 116.48, 111.67, 34.02, 30.40.

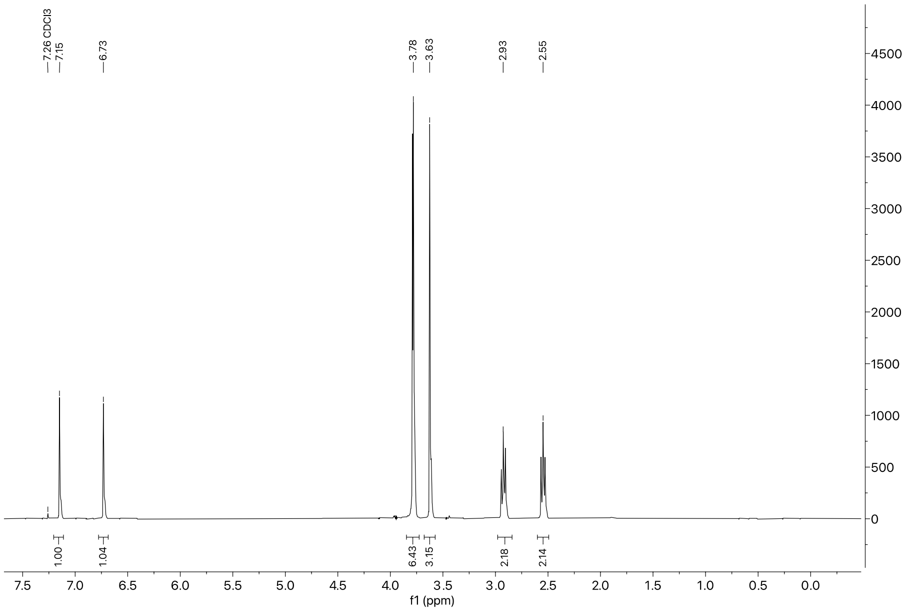

Figure S11. ^{1}H NMR spectrum for methyl 3-(2-iodo-4,5-dimethoxyphenyl) propanoate (**10**). ^{1}H NMR (400 MHz, $CDCl_3$) δ 7.15 (s, 1H), 6.73 (s, 1H), 3.78 (2x s, 6H), 3.63 (s, 3H), 2.93 (t, *J* = 7.2 Hz, 2H), 2.55 (t, *J* = 7.2 Hz, 2H).

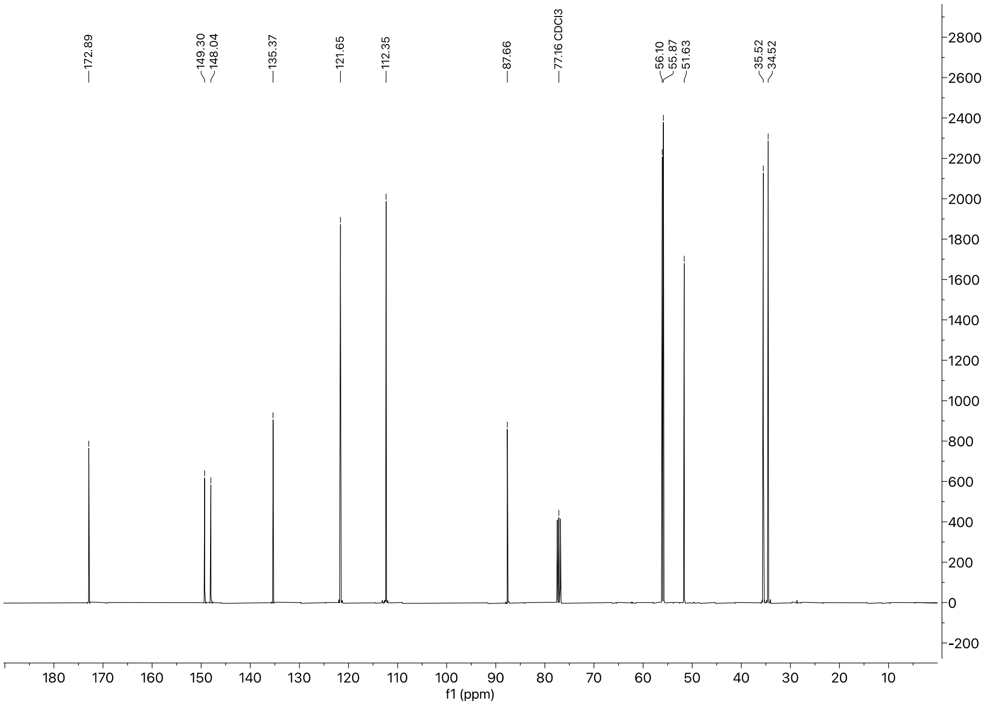

Figure S12. ^{13}C NMR spectrum for methyl 3-(2-iodo-4,5-dimethoxyphenyl) propanoate (**10**). ^{13}C NMR (101 MHz, $CDCl_3$) δ 172.89, 149.30, 148.04, 135.37, 121.65, 112.35, 87.66, 56.10, 55.87, 51.63, 35.52, 34.52.

Figure S13. ^{1}H NMR spectrum for methyl 3-(2-iodo-4,5-dimethoxyphenyl)propanoate (**11**). ^{1}H NMR (400 MHz, $CDCl_3$) δ 7.01 (s, 1H), 6.81 (s, 1H), 3.89 (d, *J* = 16.2 Hz, 6H), 3.67 (s, 3H), 3.10 (t, *J* = 7.5 Hz, 2H), 2.69 (t, *J* = 7.5 Hz, 2H).

Figure S14. ^{13}C NMR spectrum for methyl 3-(2-iodo-4,5-dimethoxyphenyl)propanoate (**11**). ^{13}C NMR (101 MHz, $CDCl_3$) δ 172.70, 152.75, 147.74, 138.92, 118.30, 114.26, 112.27, 103.35, 77.36, 56.26, 56.18, 51.90, 35.01, 29.78, 29.41.

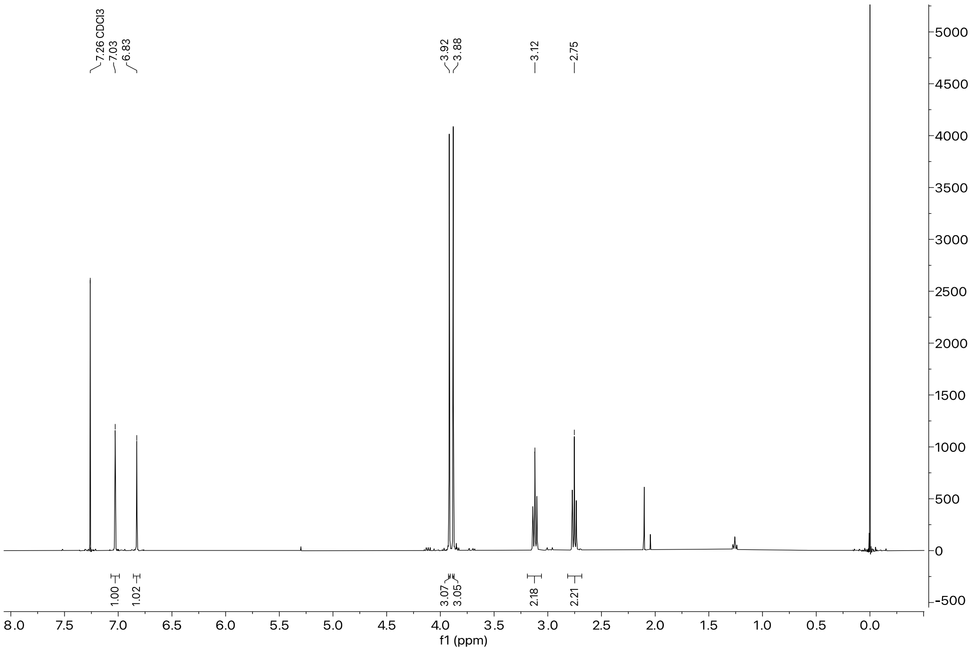

Figure S15. ^{1}H NMR spectrum for 3-(2-cyano-4,5-dimethoxyphenyl) propanoic acid (**12**). ^{1}H NMR (400 MHz, $CDCl_3$) δ 7.03 (s, 1H), 6.83 (s, 1H), 3.92 (s, 3H), 3.88 (s, 3H), 3.12 (t, J = 7.5 Hz, 2H), 2.75 (t, J = 7.5 Hz, 2H).

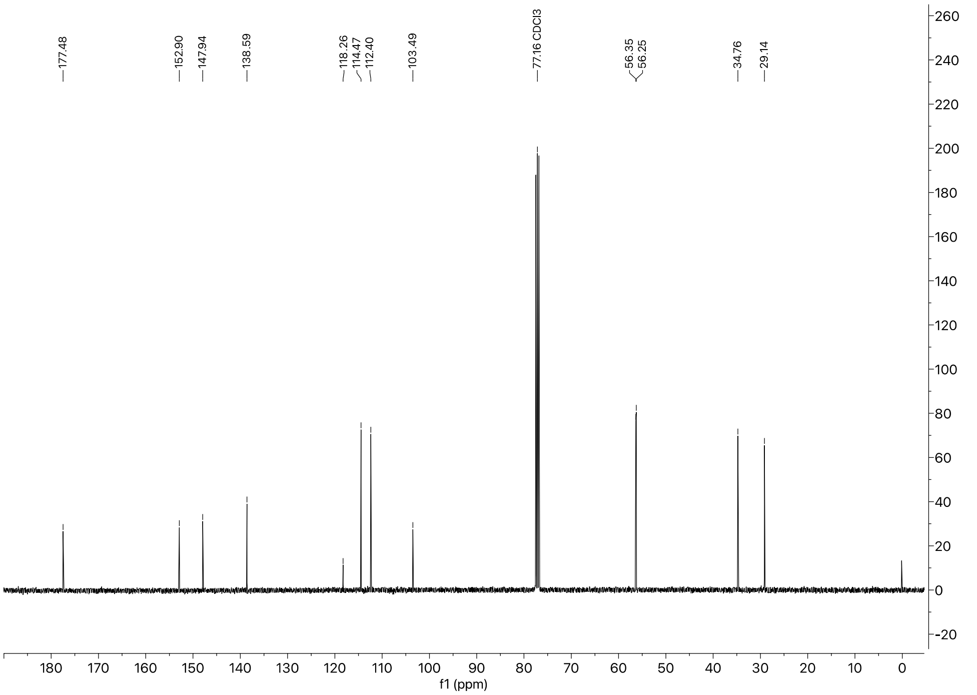

Figure S16. ^{13}C NMR spectrum for 3-(2-cyano-4,5-dimethoxyphenyl) propanoic acid (**12**). ^{13}C NMR (101 MHz, $CDCl_3$) δ 177.48, 152.90, 147.94, 138.59, 118.26, 114.47, 112.40, 103.49, 56.35, 56.25, 34.76, 29.14.

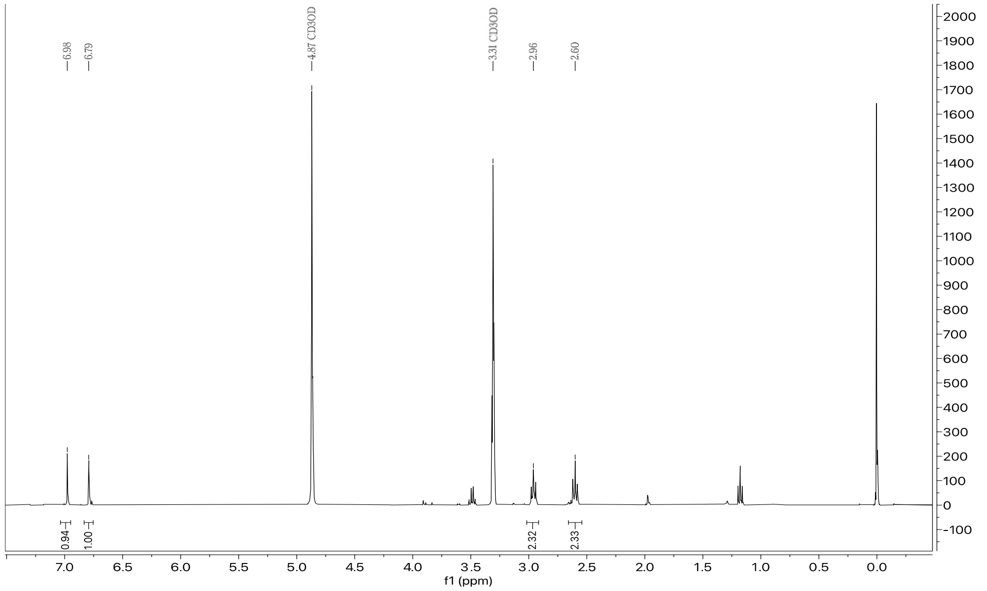

Figure S17. ^{1}H NMR spectrum for 3-(2-cyano-4,5-dihydroxyphenyl) propanoic acid (**5**). ^{1}H NMR (400 MHz, CD_3OD) δ 6.98 (s, 1H), 6.79 (s, 1H), 2.96 (t, J = 7.3 Hz, 2H), 2.60 (t, J = 7.3 Hz, 2H).

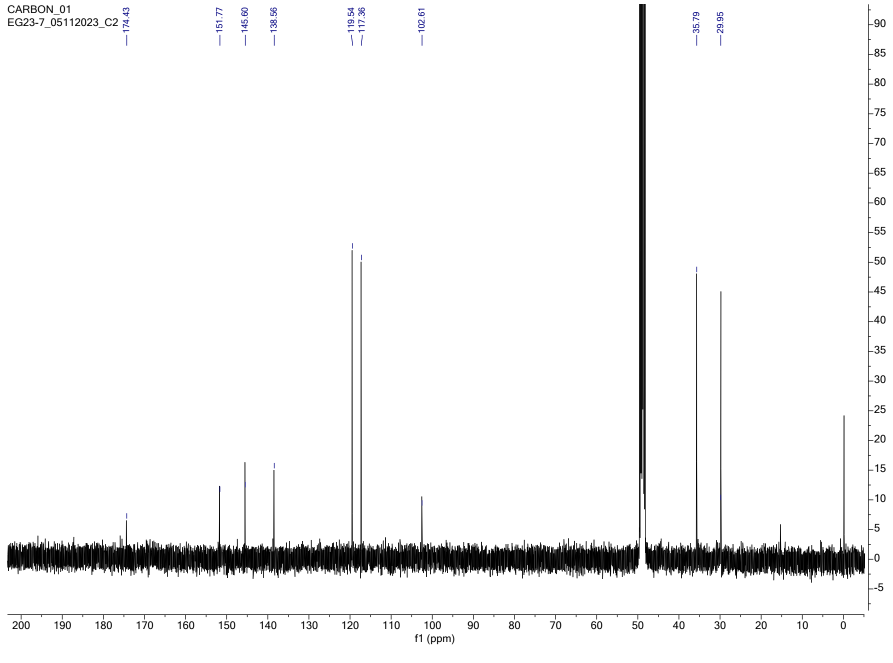

Figure S18. ^{13}C NMR spectrum for 3-(2-cyano-4,5-dihydroxyphenyl) propanoic acid (**5**). ^{13}C NMR (101 MHz, CD_3OD) δ 174.43, 151.77, 145.60, 138.56, 119.54, 117.36, 102.61, 35.79, 29.95.

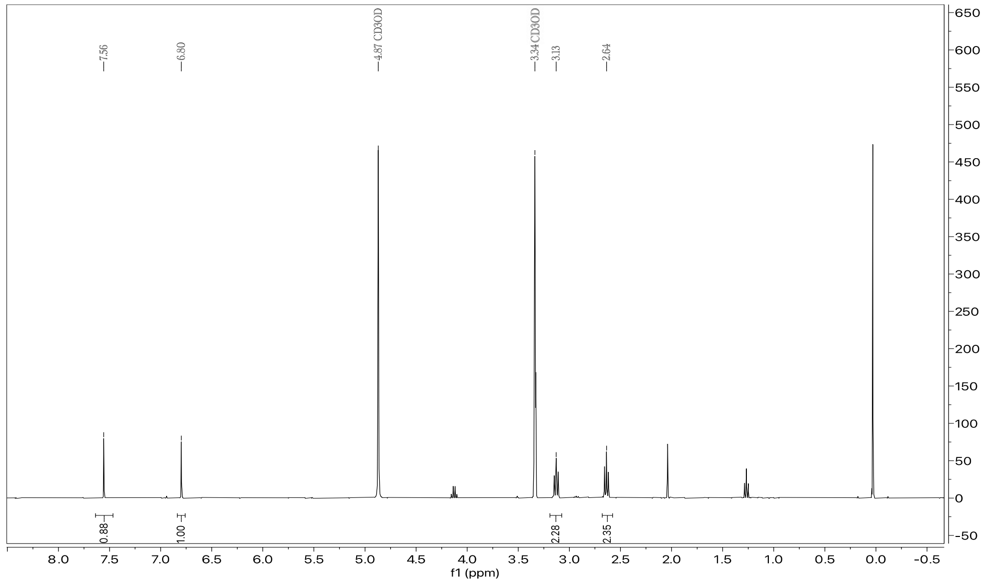

Figure S19. ^{1}H NMR spectrum for 3-(4,5-dihydroxy-2-nitrophenyl) propanoic acid (**6**). ^{1}H NMR (400 MHz, CD_3OD) δ 7.56 (s, 1H), 6.80 (s, 1H), 3.13 (t, J = 7.2 Hz, 2H), 2.64 (t, J = 7.1 Hz, 2H).

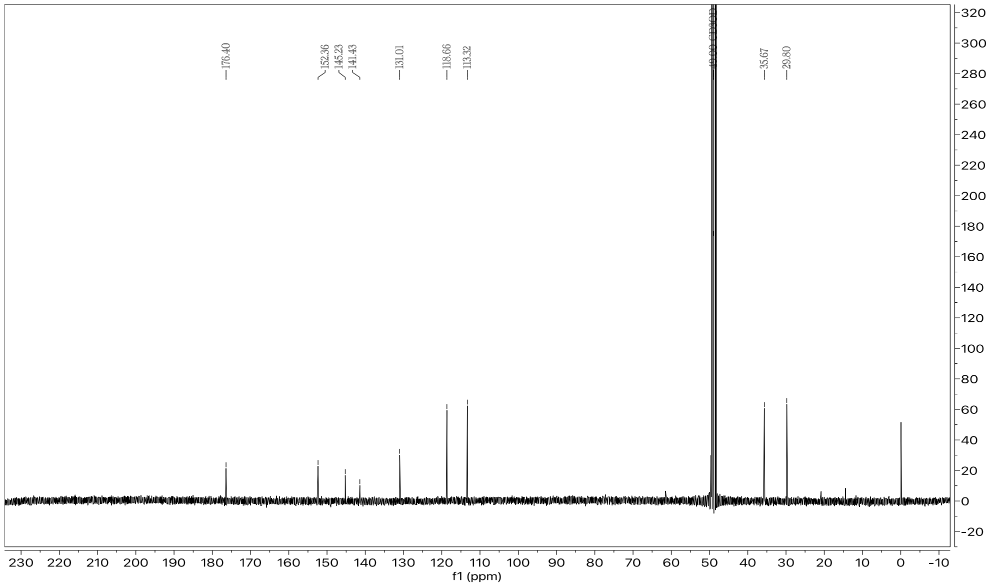

Figure S20. ^{13}C NMR spectrum for 3-(4,5-dihydroxy-2-nitrophenyl) propanoic acid (**6**). ^{13}C NMR (101 MHz, CD_3OD) δ 176.40, 152.36, 145.23, 141.43, 131.01, 118.66, 113.32, 35.67, 29.80.

HPLC Chromatograms

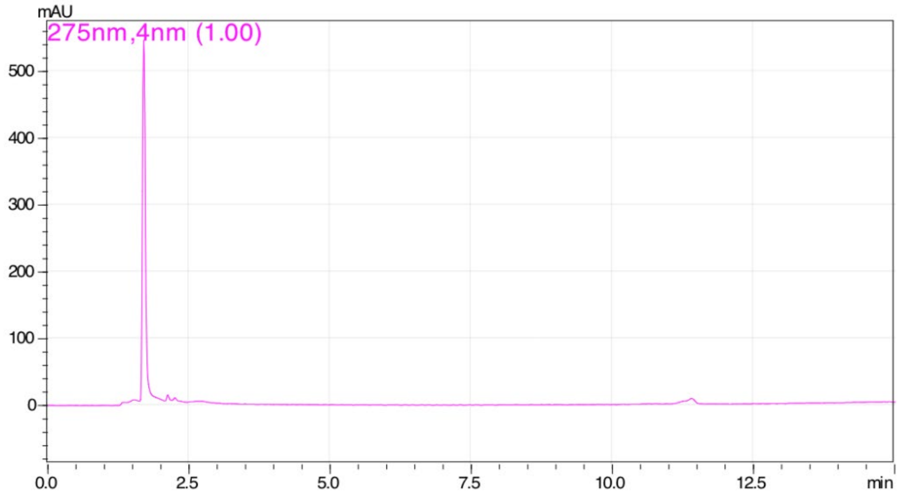

Figure S21. HPLC chromatogram for 3-(2-bromo-4,5-dihydroxyphenyl)propanoic acid (6-BromoDHHCA, **4**) at 275 nm. Kinetex Phenyl-hexyl; 0.8 mL/min flow rate; Buffer A = 5% acetonitrile, Buffer B = 20% acetonitrile; at 0 min = 80% Buffer A, 2 min = 80% Buffer A, 5 min = 70% Buffer A, 14 min = 50% Buffer A.

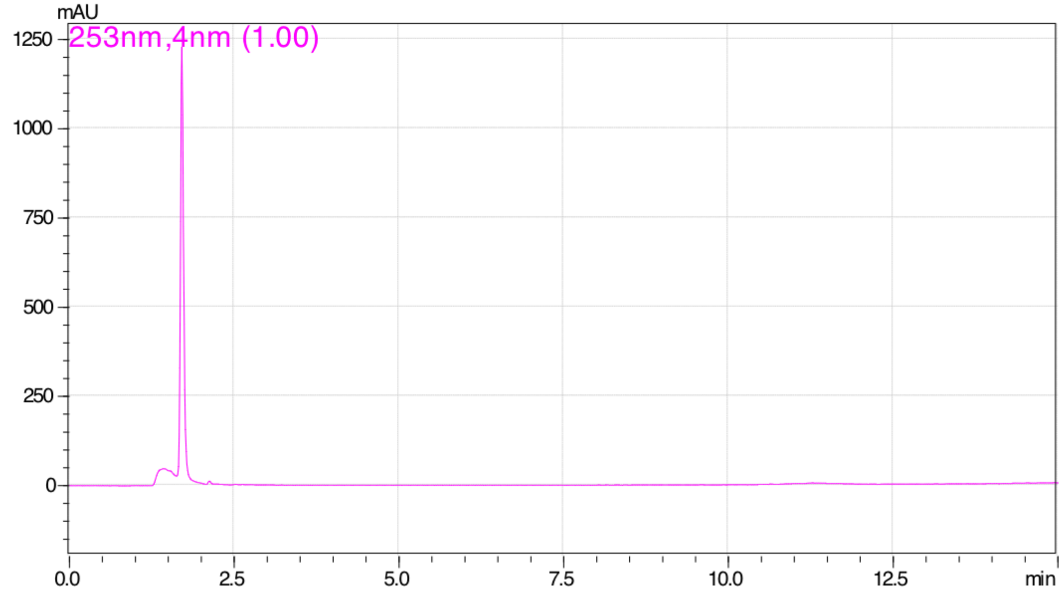

Figure S22. HPLC chromatogram for 3-(2-cyano-4,5-dihydroxyphenyl)propanoic acid (CyanoDHHCA, **5**) at 253 nm. Kinetex Phenyl-hexyl column; 0.8 mL/min flow rate; Buffer A = 5% acetonitrile, Buffer B = 20% acetonitrile; at 0 min = 80% Buffer A, 2 min = 80% Buffer A, 5 min = 70% Buffer A, 14 min = 50% Buffer A.

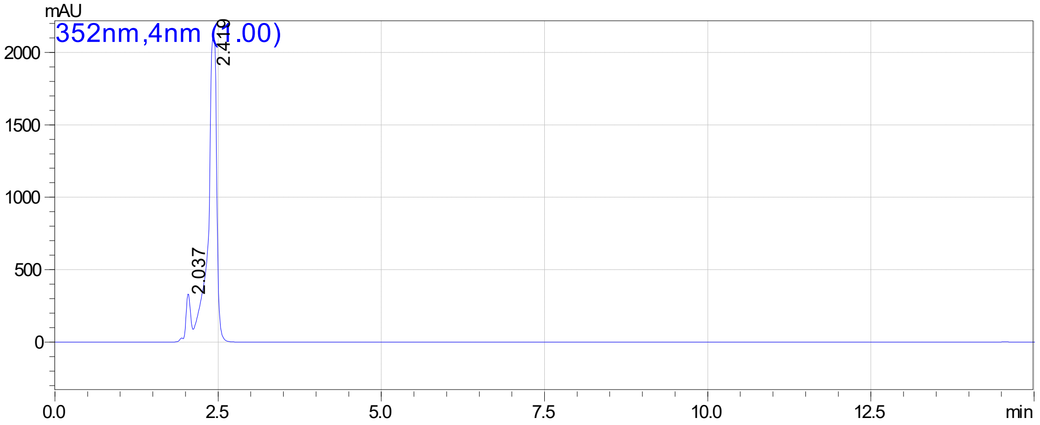

Figure S23. HPLC chromatogram for 3-(4,5-dihydroxy-2-nitrophenol)propanoic acid (6-nitroDHHCA, **6**) at 352 nm. Kinetex Phenyl-hexyl; 0.5 mL/min flow rate; Buffer A = 2.0% trifluoroacetic acid (TFA), Buffer B = 20% acetonitrile; at 0 min = 80% Buffer A, 2 min = 70% Buffer A, 10 min = 50% Buffer A, 15 min = 40% Buffer A.

pK$_a$ Spectra

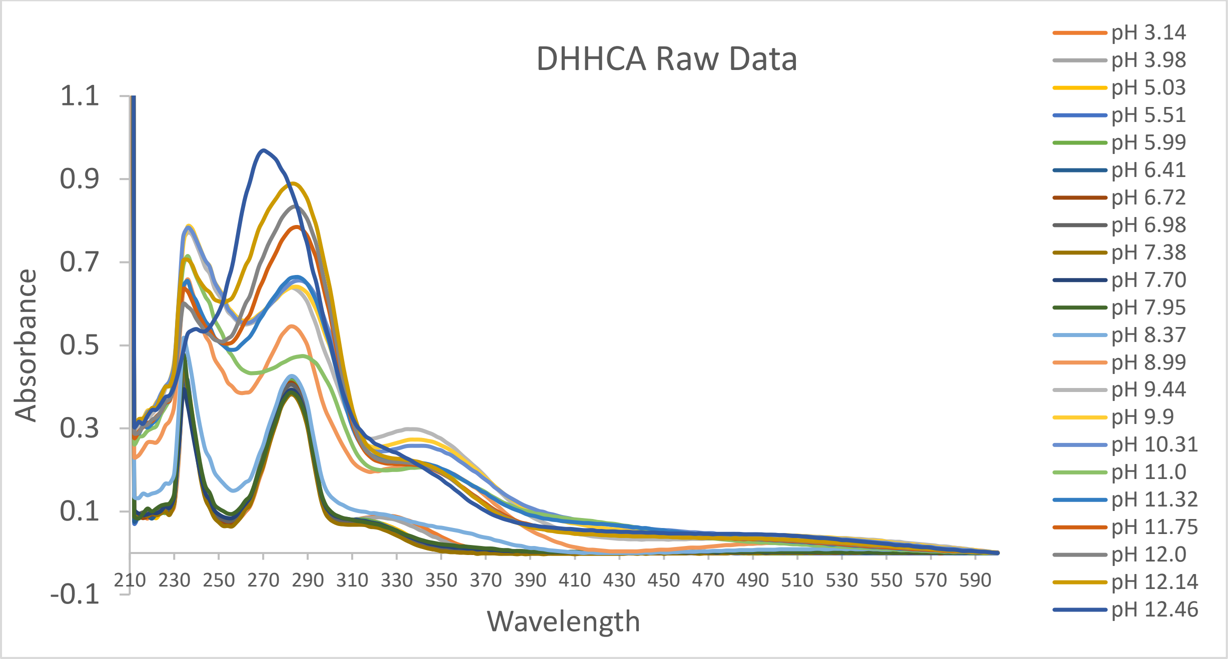

Figure S24. Wavelength (nm) vs absorbance for DHHCA (**3**) using the values obtained from the microplate reader Synergy HTX 15032517. No normalizations were made.

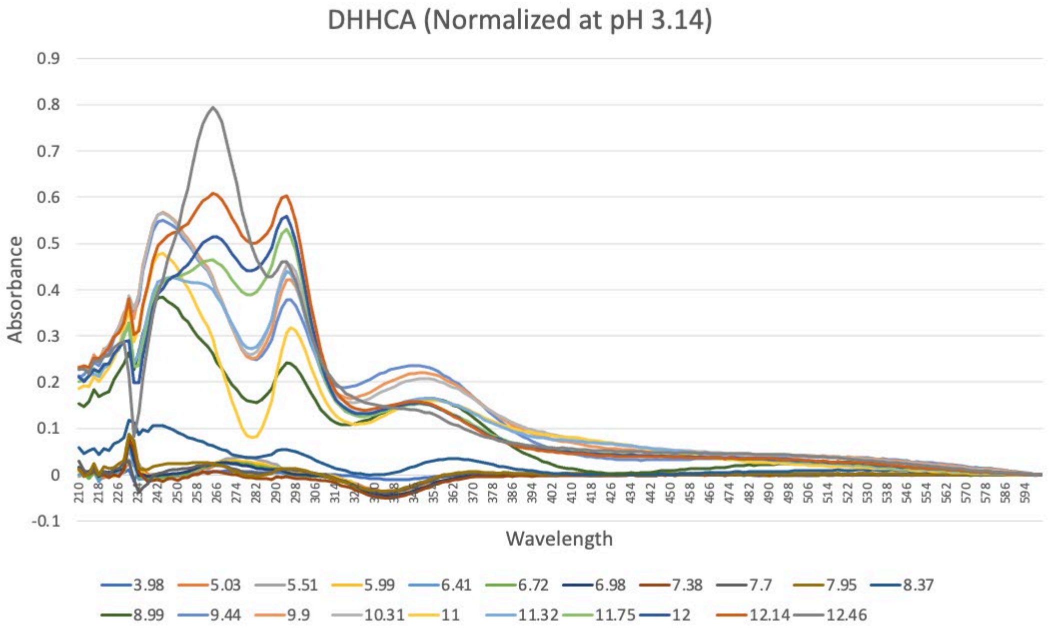

Figure S25. pH vs absorbance for DHHCA (**3**) using the values obtained from the microplate reader Synergy HTX 15032517. The data was normalized to pH 3.14 (abs = 0).

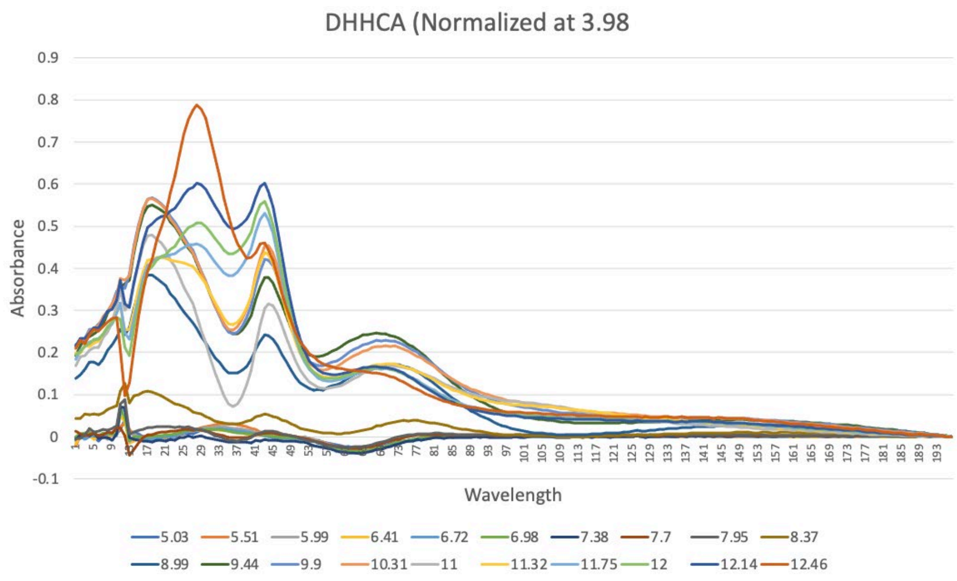

Figure S26. pH vs absorbance for DHHCA (**3**) using the values obtained from the microplate reader Synergy HTX 15032517. The data was normalized to pH 3.98 (abs = 0).

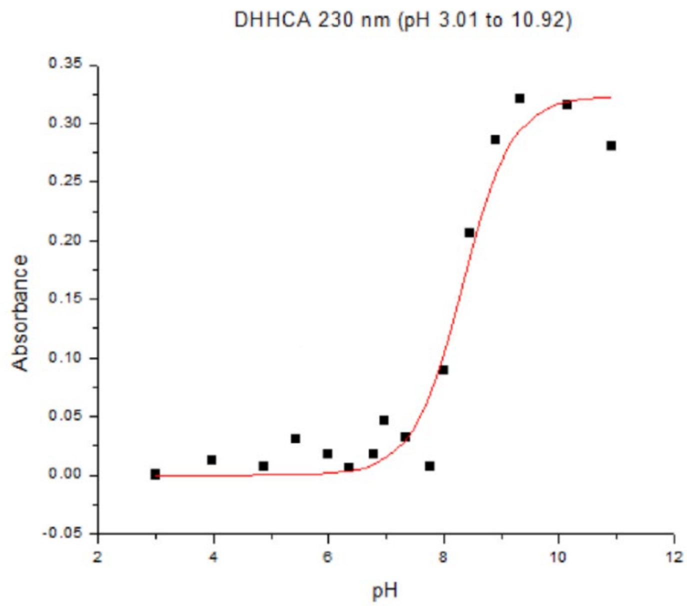

Figure S27. pH vs absorbance for DHHCA (**3**) using the data from Figure S24 (above) with pH range from 3.01 to 10.92. $R^2 = 0.99$; $\lambda_{max} = 230$ nm; $pK_{a1} = 8.87 \pm 0.12$.

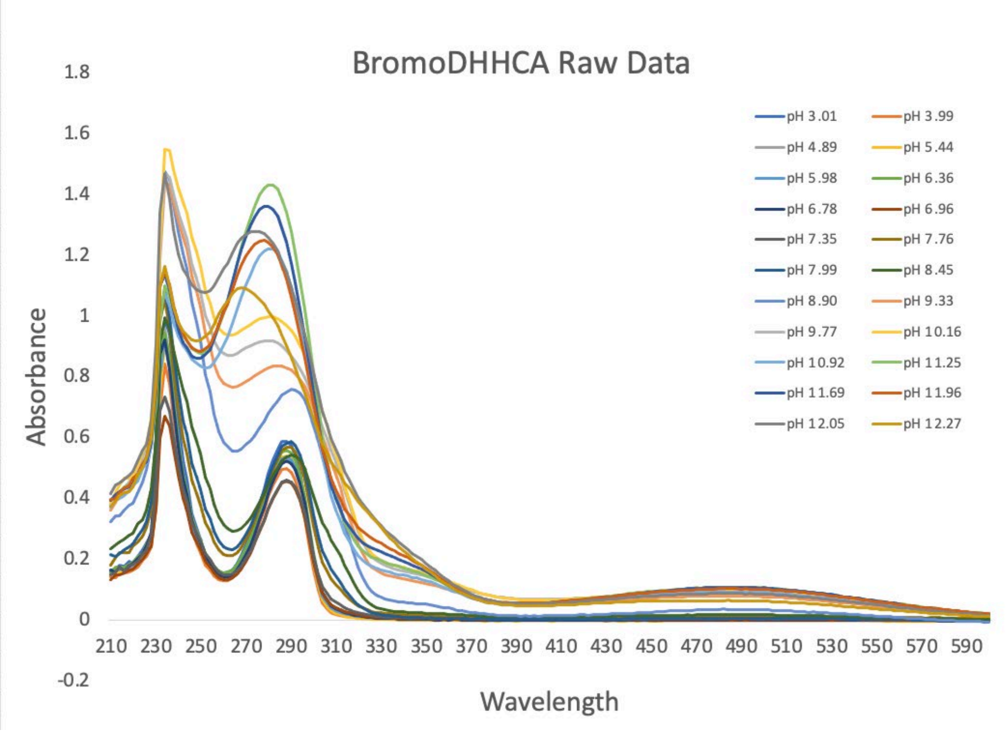

Figure S28. pH vs absorbance for 6-BromoDHHCA (**4**) using the values obtained from the microplate reader Synergy HTX 15032517. No normalizations were made.

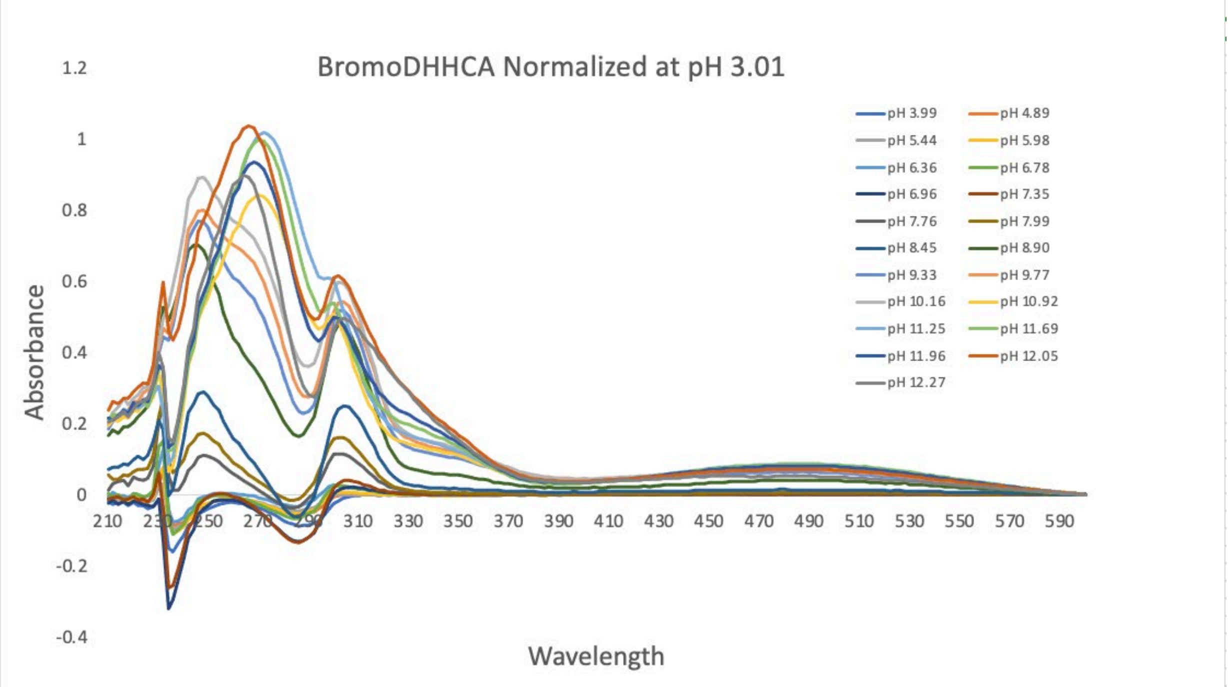

Figure S29. pH vs absorbance for 6-BromoDHHCA (**4**) using the values obtained from the microplate reader Synergy HTX 15032517. The data was normalized to pH 3.01 (abs = 0).

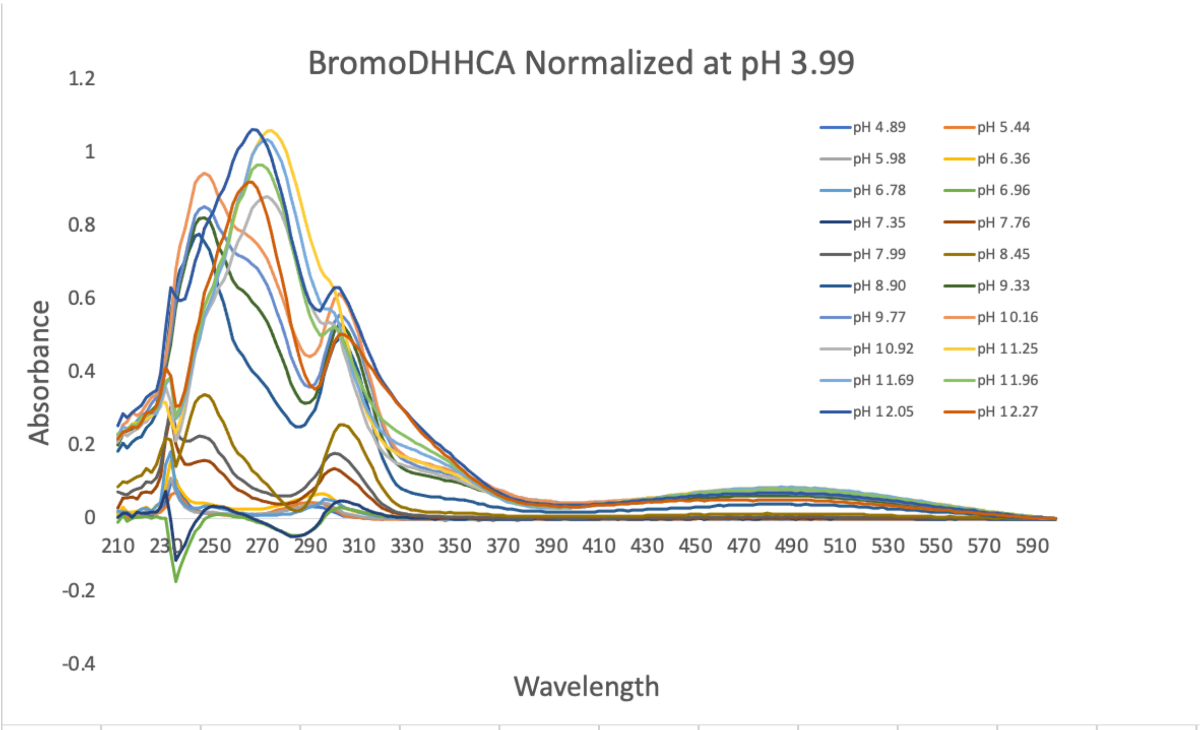

Figure S30. pH vs absorbance for 6-BromoDHHCA (**4**) using the values obtained from the microplate reader Synergy HTX 15032517. The data was normalized to pH 3.99 (abs = 0).

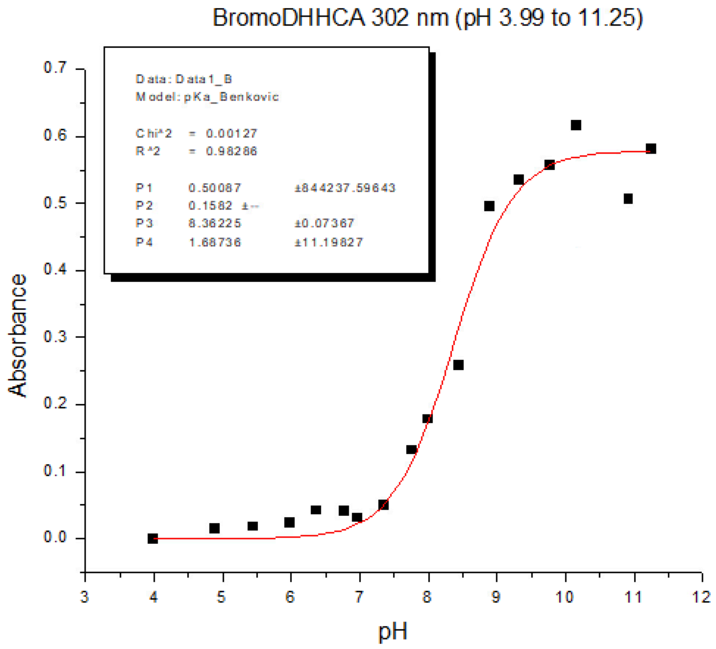

Figure S31. pH vs absorbance for 6-BromoDHHCA (**4**) using the data from Figure S30. R^2 = 0.98; λ_{max} = 302 nm; pK_{a1} = 8.36 ± 0.07.

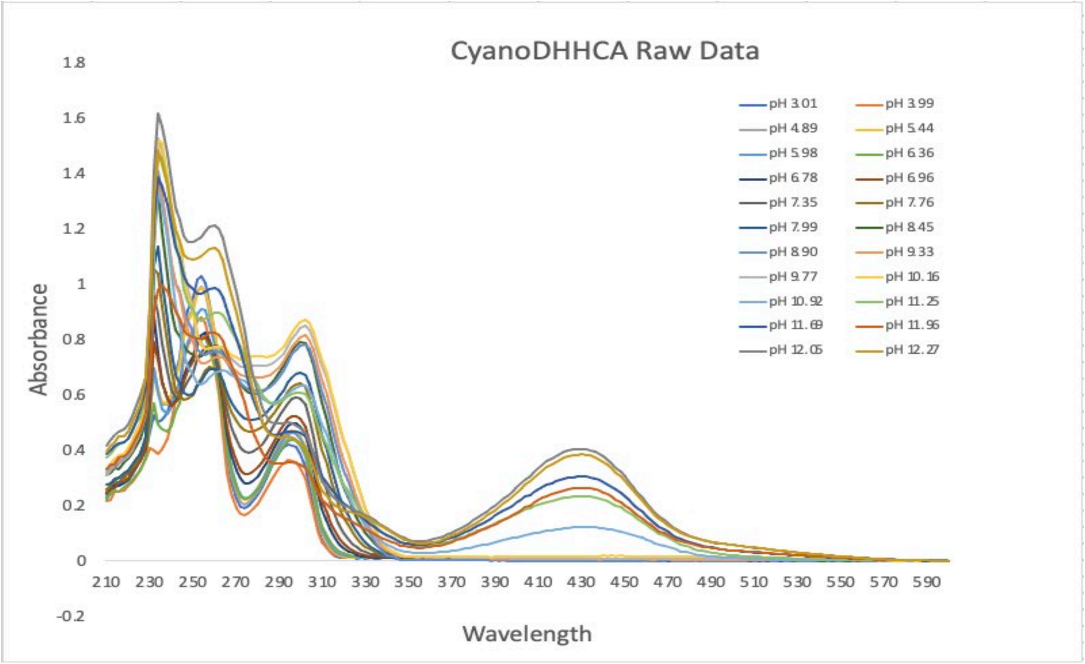

Figure S32. pH vs absorbance for 6-CyanoDHHCA (**5**) using the values obtained from the microplate reader Synergy HTX 15032517. The data was not normalized.

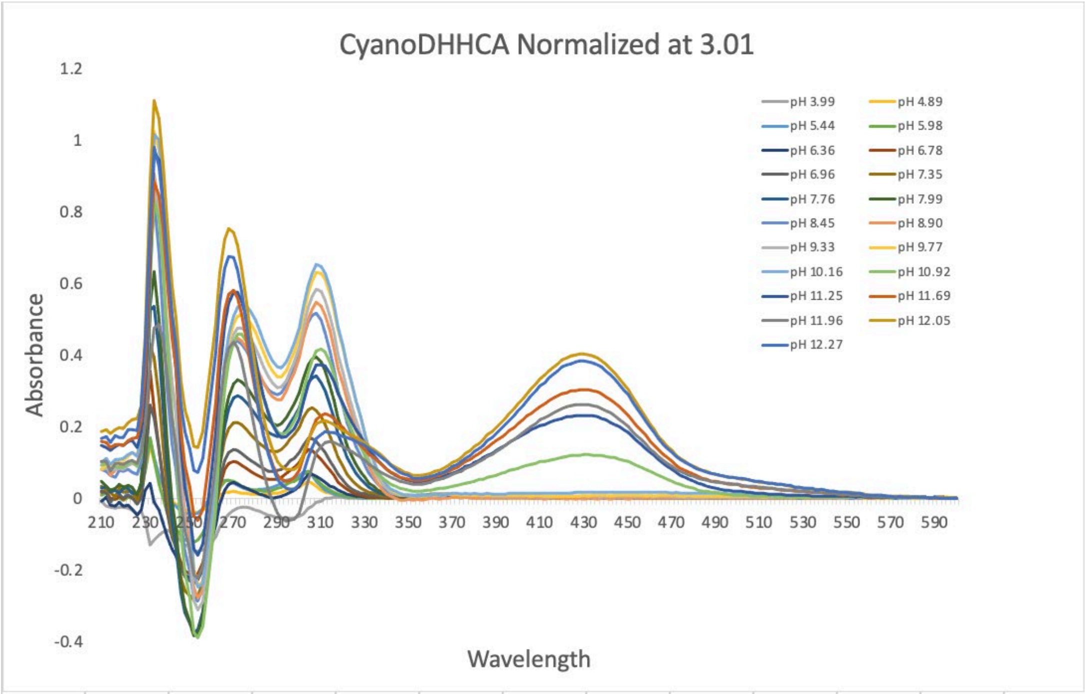

Figure S33. pH vs absorbance for 6-CyanoDHHCA (**5**) using the values obtained from the microplate reader Synergy HTX 15032517. The data was normalized to pH 3.01 (abs = 0).

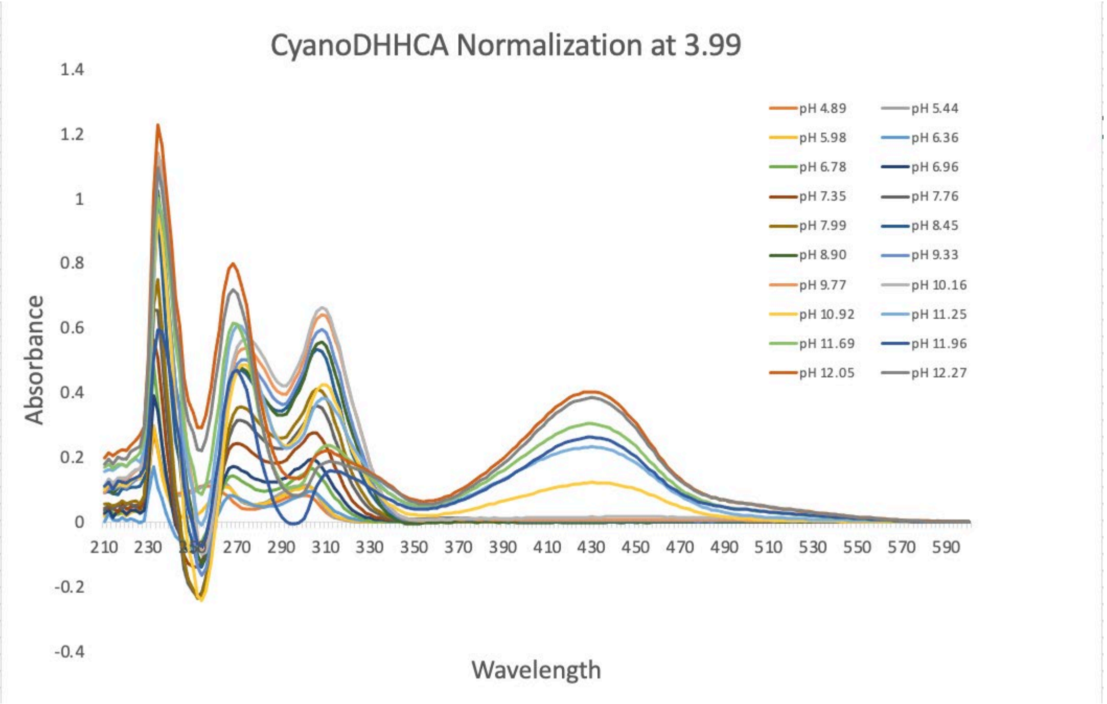

Figure S34. pH vs absorbance for 6-CyanoDHHCA (**5**) using the values obtained from the microplate reader Synergy HTX 15032417. The data was normalized to pH 3.99 (abs = 0).

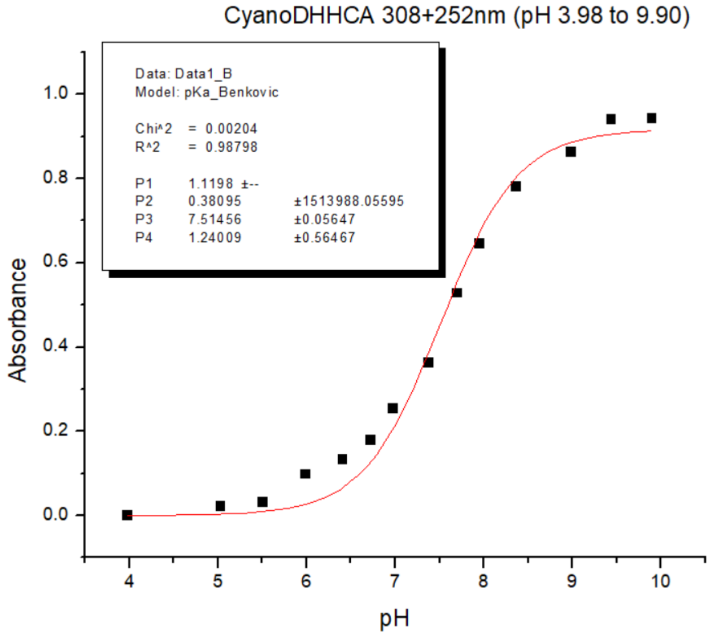

Figure S35. pH vs absorbance for 6-CyanoDHHCA (**5**) using the data from Figure S34. R^2 = 0.99; λ_{max} = 308 + 252 nm; pK_{a1} = 7.51 ± 0.06.

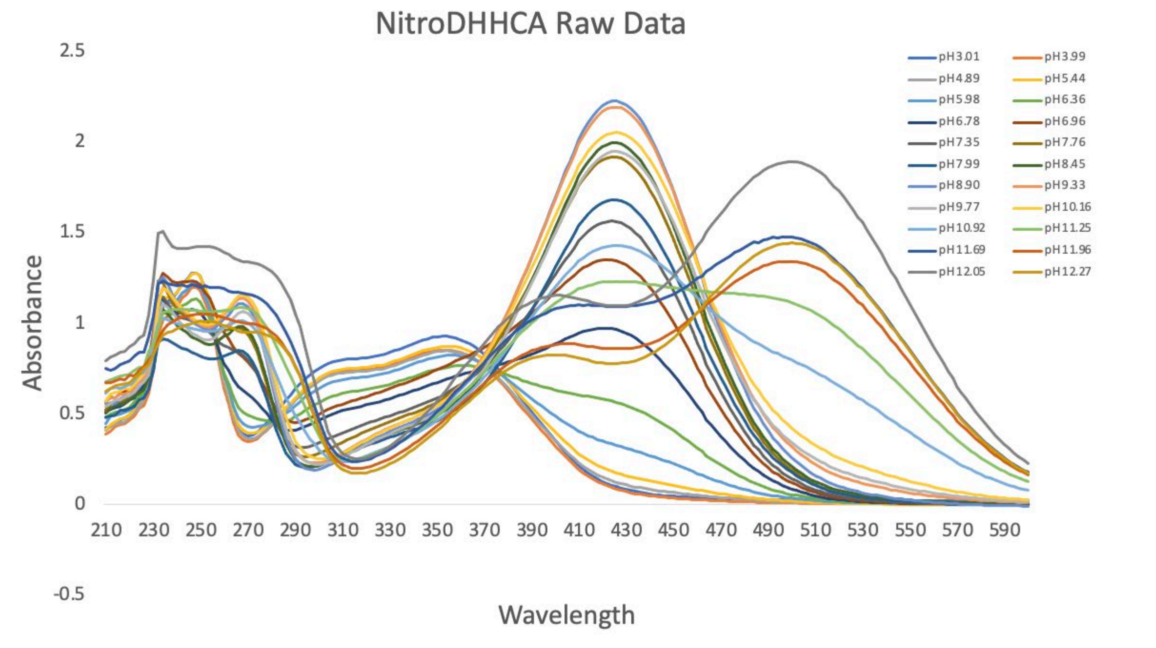

Figure S36. pH vs absorbance for 6-NitroDHHCA (**6**) using the values obtained from the microplate reader Synergy HTX 15032417. The data was not normalized.

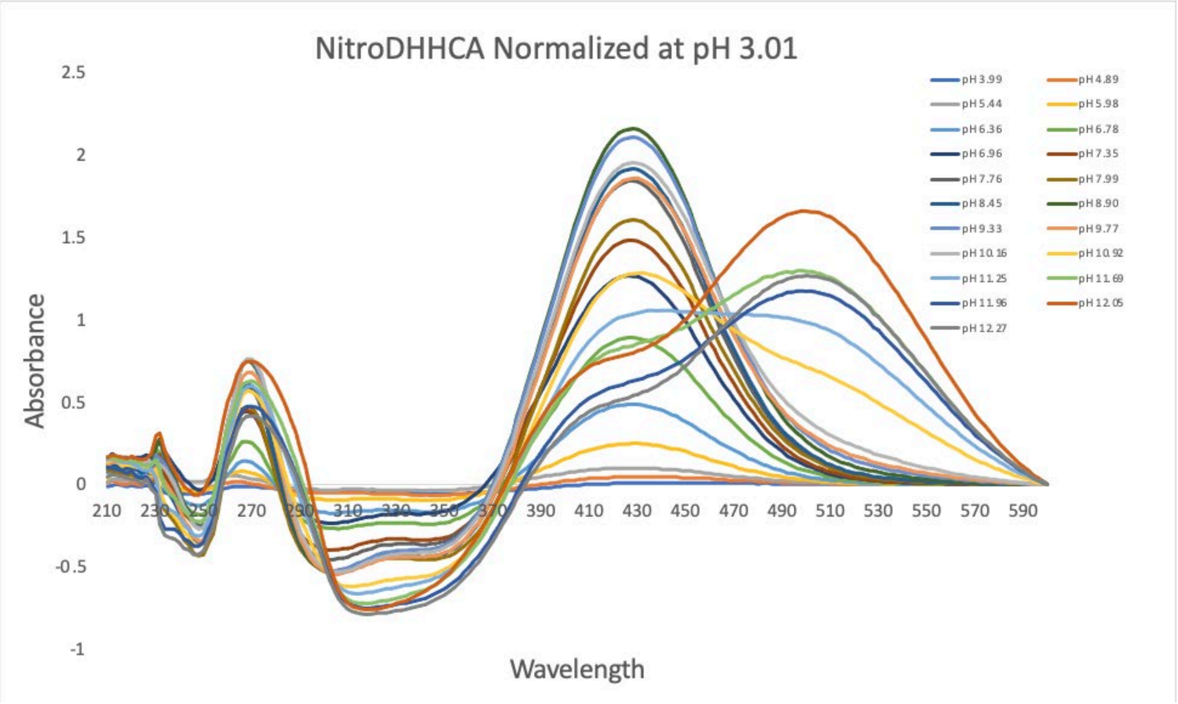

Figure S37. pH vs absorbance for 6-NitroDHHCA (**6**) using the values obtained from the microplate reader Synergy HTX 15032417. The data was normalized at pH 3.01 (abs = 0).

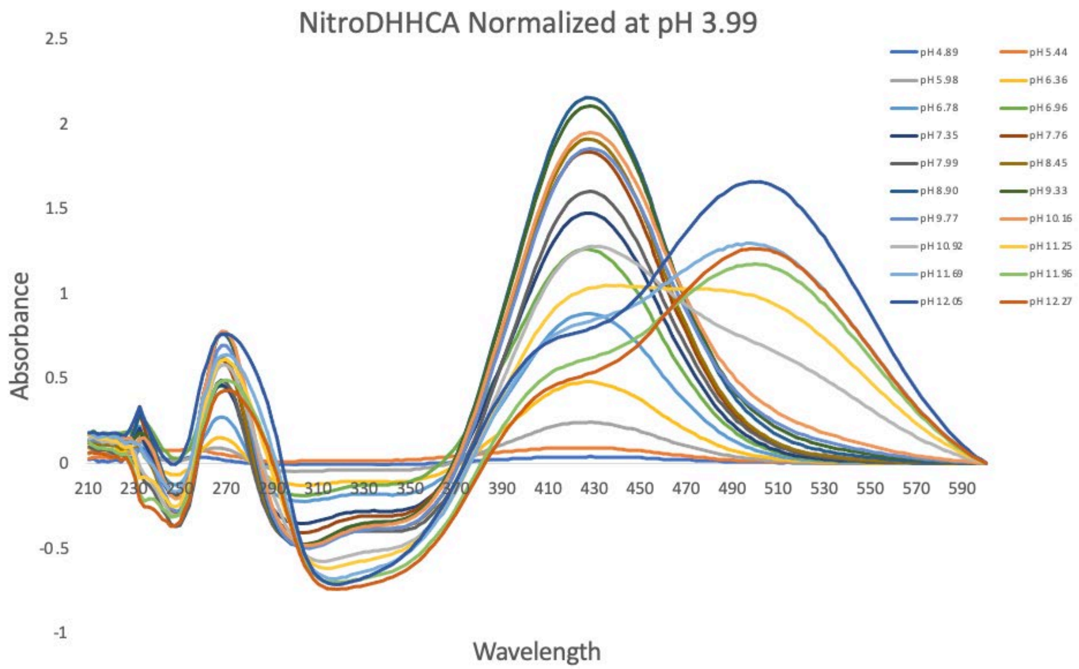

Figure S38. pH vs absorbance for 6-NitroDHHCA (**6**) using the values obtained from the microplate reader Synergy HTX 15032417. The data was normalized at pH 3.99 (abs = 0).

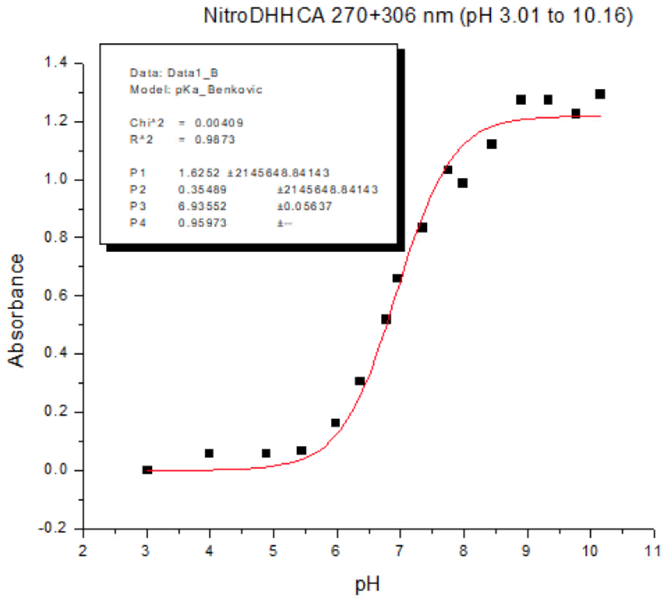

Figure S39. pH vs absorbance for 6-NitroDHHCA (**6**) using the data from Figure S38. R^2 = 0.96; λ_{max} = 270 + 306 nm; pK_{a1} = 6.94 ± 0.06.

Cyclic Voltammetry

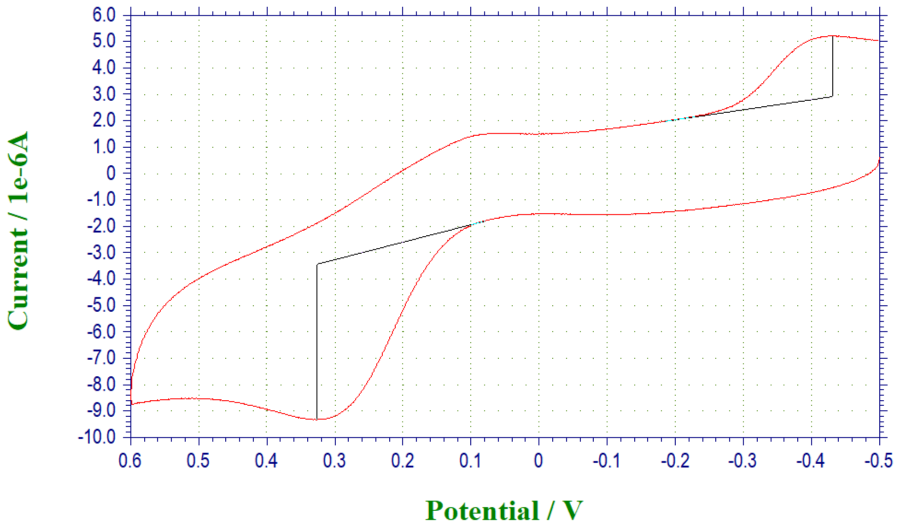

Figure S40. Cyclic voltammogram of L-DOPA (**1**) in phosphate buffer at pH 7.4 on a glassy carbon electrode (*d* = 2mm).

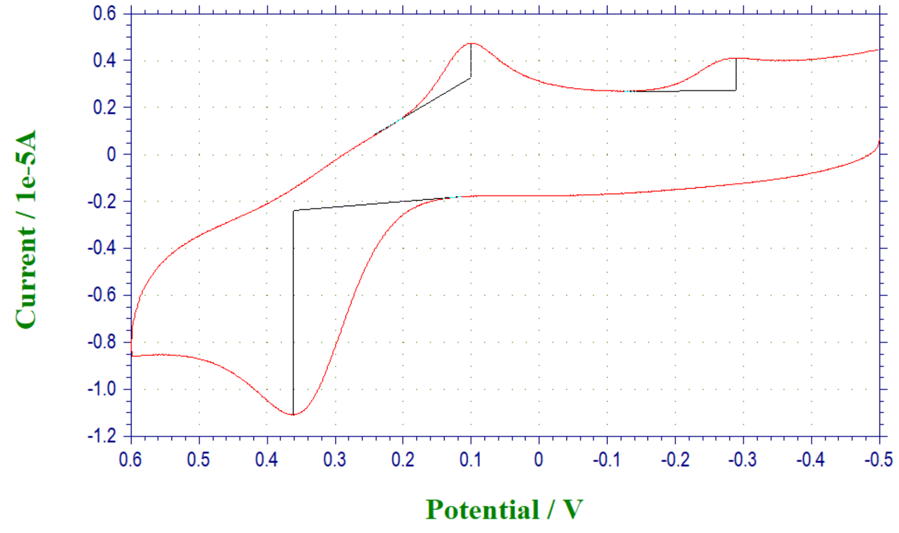

Figure S41. Cyclic voltammogram of L-DOPA (**1**) in phosphate buffer pH 6.0 on a glassy carbon electrode (*d* = 2mm).

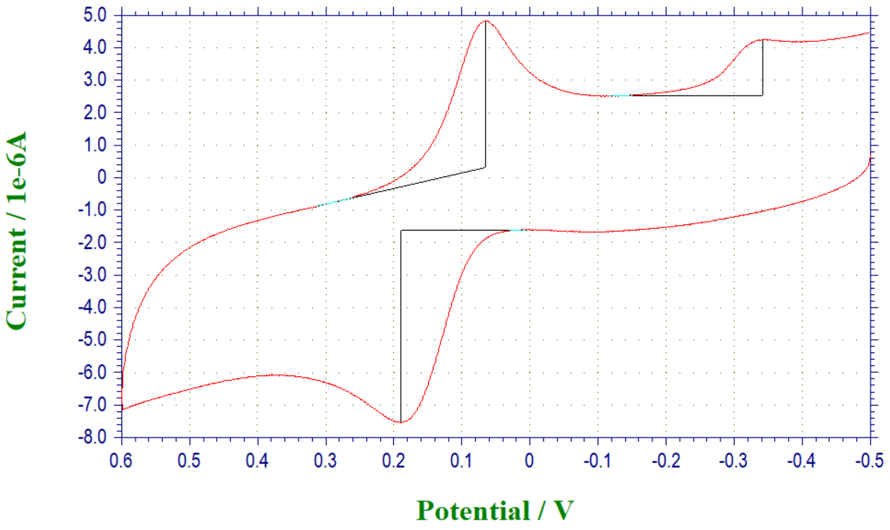

Figure S42. Cyclic voltammogram of DA (**2**) in phosphate buffer pH 7.4 on a glassy carbon electrode (*d* = 2mm).

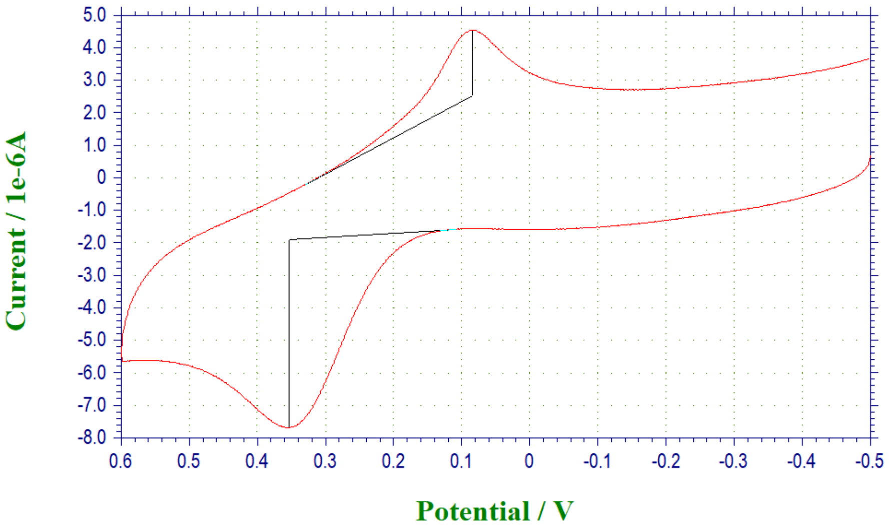

Figure S43. Cyclic voltammogram of DA (**2**) in phosphate buffer pH 6.0 on a glassy carbon electrode (*d* = 2mm).

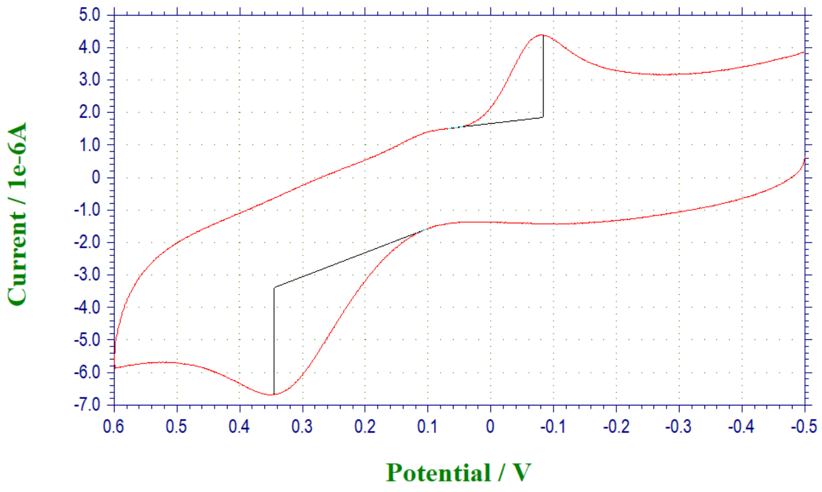

Figure S44. Cyclic voltammogram of DHHCA (**3**) in phosphate buffer at pH 7.4 on a glassy carbon electrode (*d* = 2mm).

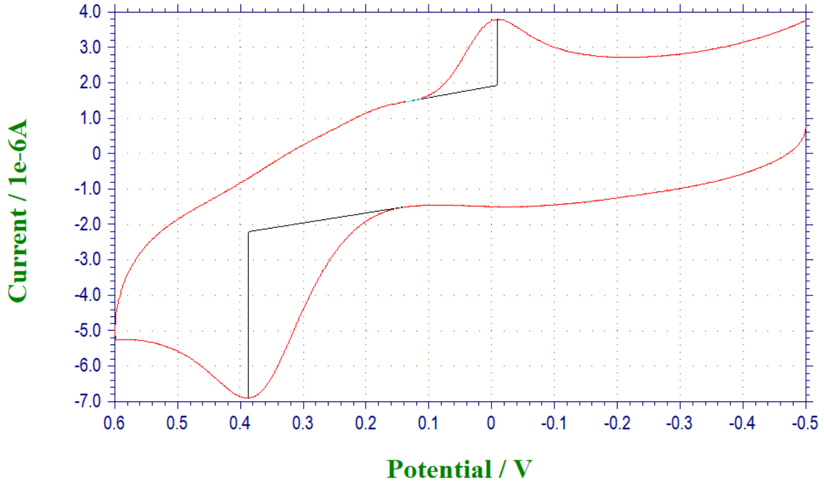

Figure S45. Cyclic voltammogram of DHHCA (**3**) in phosphate buffer at pH 6.0 on a glassy carbon electrode (*d* = 2mm).

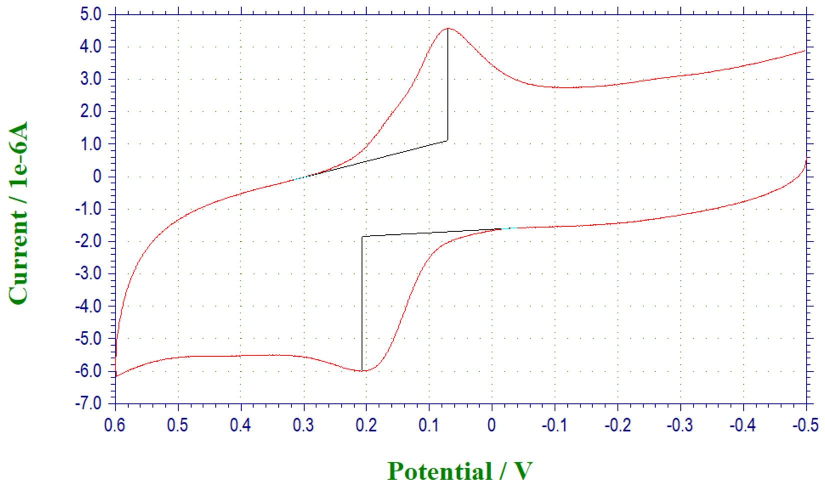

Figure S46. Cyclic voltammogram of 6-bromoDHHCA (**4**) in phosphate buffer at pH 7.4 on a glassy carbon electrode (*d* = 2mm).

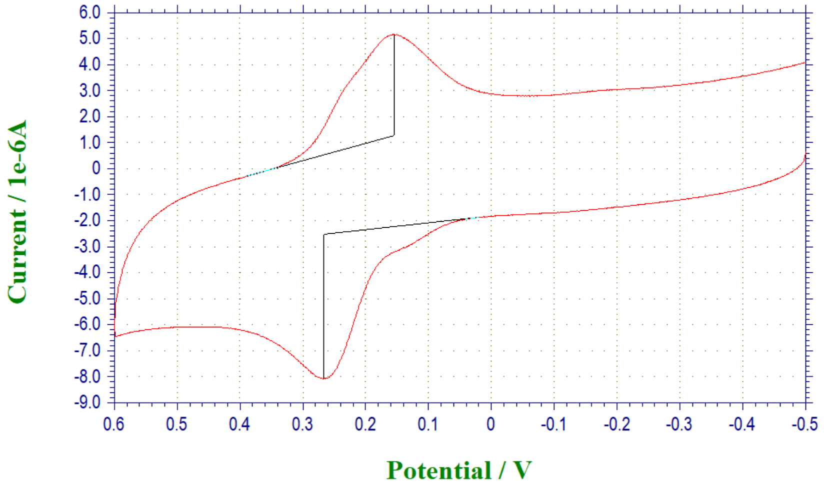

Figure S47. Cyclic voltammogram of 6-bromoDHHCA (**4**) in phosphate buffer at pH 6.0 on a glassy carbon electrode (*d* = 2mm).

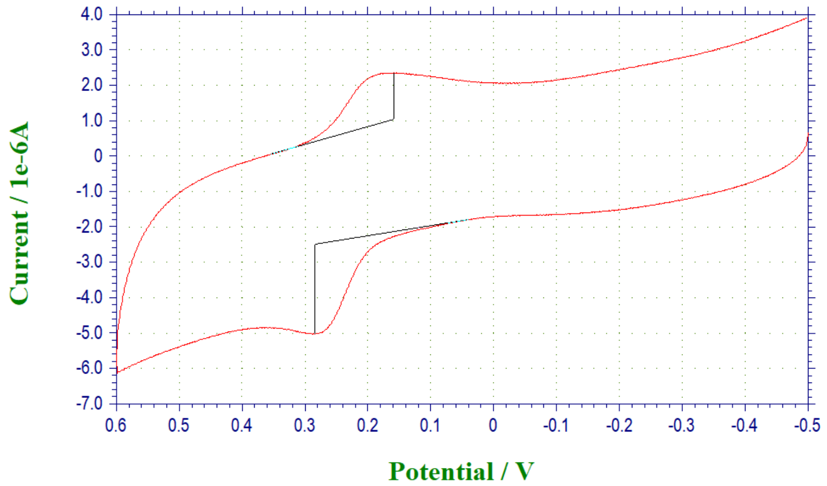

Figure S48. Cyclic voltammogram of 6-cyanoDHHCA (**5**) in phosphate buffer at pH 7.4 on a glassy carbon electrode (*d* = 2mm).

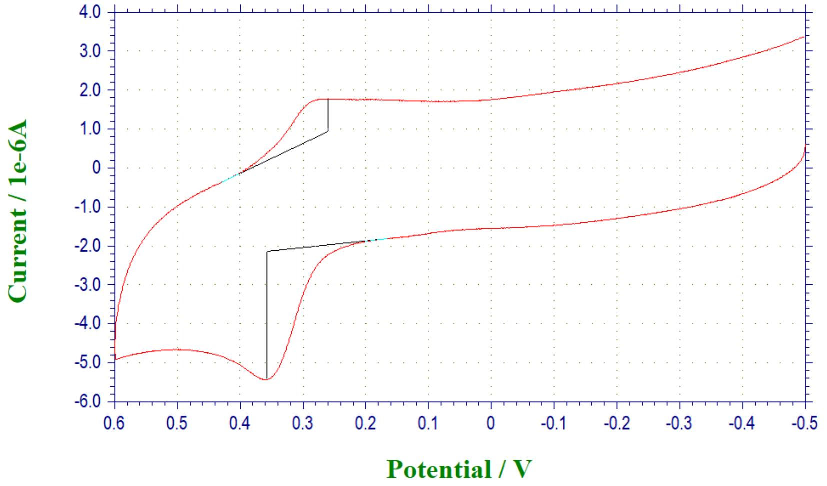

Figure S49. Cyclic voltammogram of 6-cyanoDHHCA (**5**) in phosphate buffer at pH 6.0 on a glassy carbon electrode (*d* = 2mm).

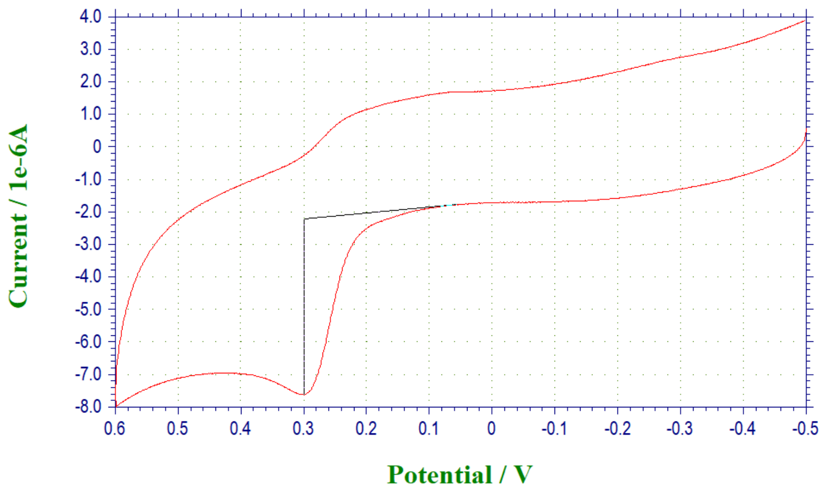

Figure S50. Cyclic voltammogram of 6-nitroDHHCA (**6**) in phosphate buffer at pH 7.4 on a glassy carbon electrode (d = 2mm).

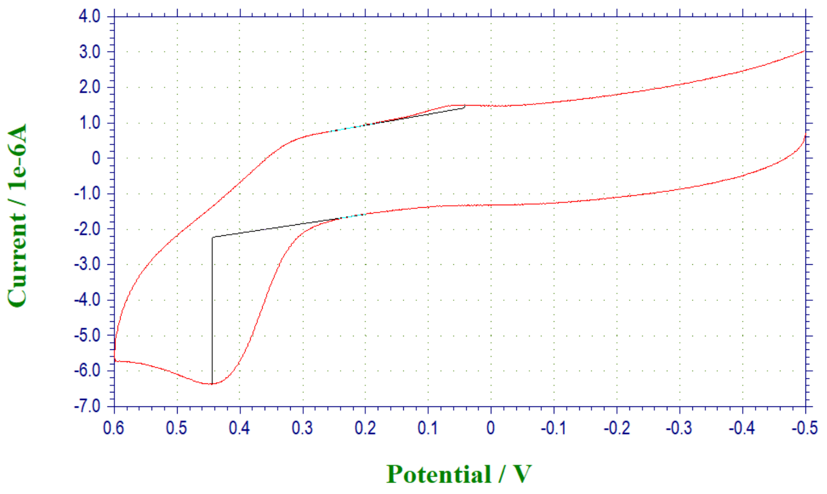

Figure S51. Cyclic voltammogram of 6-nitroDHHCA (**6**) in phosphate buffer at pH 6.0 on a glassy carbon electrode (d = 2mm).

Extinction Coefficients for steady-state cleavage products

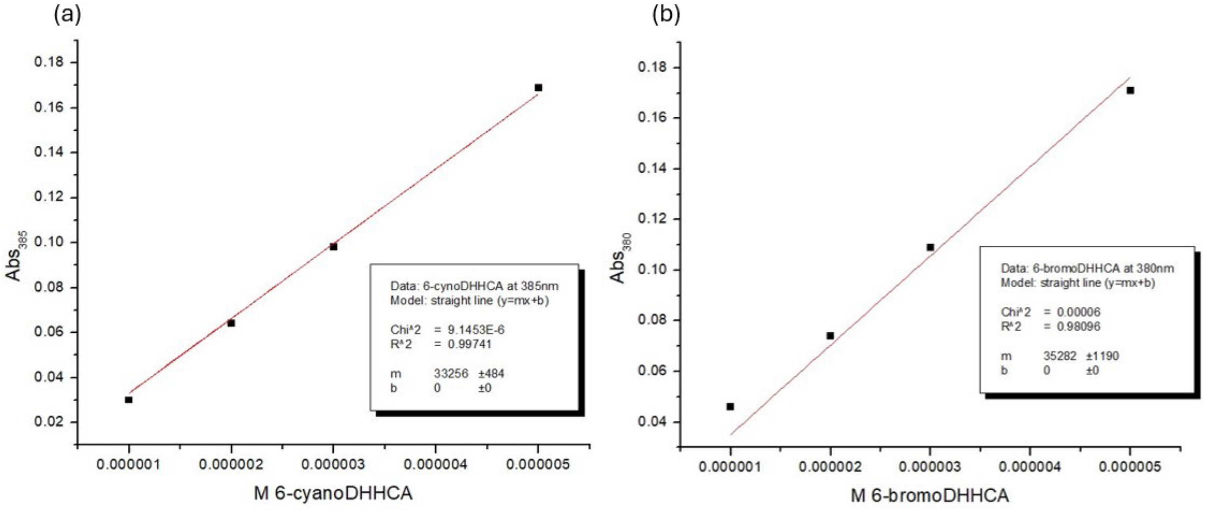

Figure S52. Beer's Law Plots for steady-state products of each DHHCA derivative (i.e. 6-bromoDHHCA (**4**), 6-cyanoDHHCA (**5**)) with LmbB1 L-DOPA 2,3-dioxygenase to determine the extinction coefficient. Various concentrations of the steady state product were achieved by reacting substrate with 10x stoichiometric excess of LmbB1 L-DOPA 2,3-dioxygenase, excess oxygen (~ 890 μM) at 22 °C in 50 mM HEPES buffer with 154 mM NaCl at pH 7.5, and allowing the reaction to reach completion before measuring the reaction product at λ_{max}. (a) Steady state cleavage product of 6-cyanoDHHCA at λ_{max} 385nm (b) Steady state cleavage product of 6-bromoDHHCA at λ_{max} 380 nm.

Michaelis-Menten Plots of DHHCA/6-x-DHHCA substrates with L-DOPA 2,3-dioxygenases

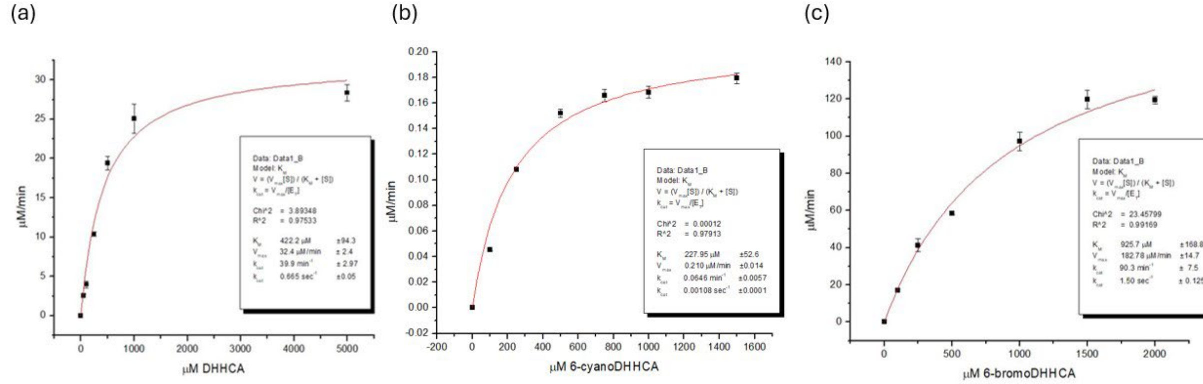

Figure S53. Plots of ShjDDO L-DOPA 2,3-dioxygenase reaction with DHHCA and synthetic DHHCA derivatives (i.e. 6- bromoDHHCA (**4**), 6-cyanoDHHCA (**5**)) as a function of substrate concentration in reaction buffer (50 mM HEPES, 10% glycerol, pH 7.50). ShjDDO L-DOPA 2,3-dioxygenase (0.5-3.25 µM) was reacted with excess oxygen and various DHHCA/derivative concentrations (0-5000 µM at 22 °C). The reaction of L-DOPA 2,3-dioxygenase with DHHCA/derivative was followed by the appearance of the steady state cleavage product using UV-Visible spectroscopy. Initial rates were obtained from the slopes of the linear regions of the progress curves and plotted versus substrate concentration to yield a hyperbola, which was fit to the Michaelis-Menten expression: $\upsilon = (V_{max}[S])/(K_M + [S])$ using standard nonlinear regression (Origin 6.0, Microcal) to obtain $k_{cat} = V_{max}/[E_T]$ and the error propagated; (a) vary DHHCA with 0.8 µM ShjDDO at 100% O_2, (b) vary 6-cyanoDHHCA with 3.5 µM ShjDDO at 100% O_2, (c) vary 6-bromoDHHCA with 2.0 µM ShjDDO at 100% O_2.

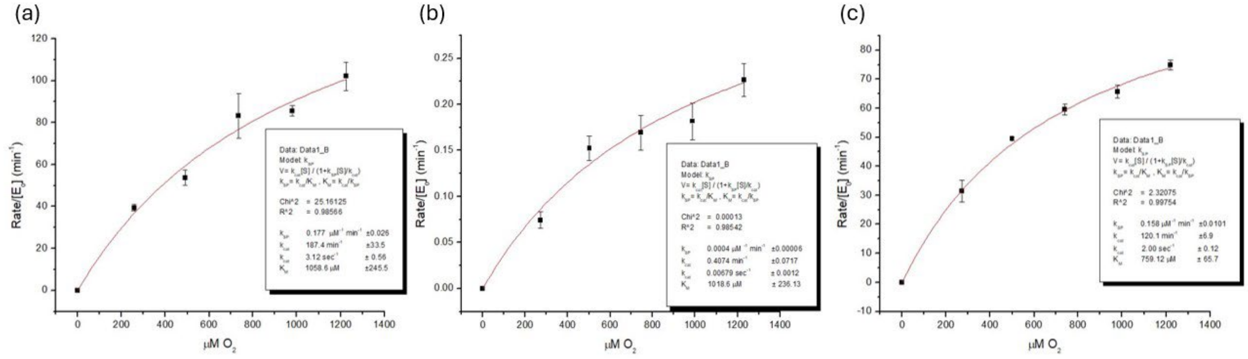

Figure S54. Plots of ShjDDO L-DOPA 2,3-dioxygenase reaction with DHHCA (**3**) and synthetic DHHCA derivatives (i.e. 6- bromoDHHCA (**4**), 6-cyanoDHHCA (**5**)) as a function of oxygen concentration in reaction buffer (50 mM HEPES, 10% glycerol, pH 7.50). ShjDDO L-DOPA 2,3-dioxygenase (1.0-2.5 μM) was reacted with excess DHHCA/derivative and various oxygen concentrations (200-1200 μM at 22°C). The reaction of L-DOPA 2,3-dioxygenase with DHHCA/derivative as a function of oxygen concentration was followed by the appearance of the steady state cleavage product using UV-Visible spectroscopy. Initial rates were obtained from the slopes of the linear regions of the progress curves and plotted versus substrate concentration to yield a hyperbola, which was fit to the following alternative derivation of the Michaelis-Menten expression: $\upsilon = (k_{SP}[S])/(1+k_{SP}\,[S]/k_{cat})$[1] using standard nonlinear regression (Origin 6.0, Microcal) to obtain k_{SP} (or k_{cat}/K_M) directly; K_M was calculated from the fitted k_{SP} and k_{cat} parameters according to the following expression: $K_M = k_{cat}/k_{SP}$ and the error propagated. (a) 2000 μM DHHCA (5x K_M, DHHCA) with 1.0 μM ShjDDO, (b) 2000 μM 6-cyanoDHHCA (10x K_M, 6-cyanoDHHCA) with 2.5 μM ShjDDO, (c) 2000 μM 6-bromoDHHCA (2x K_M,6-bromoDHHCA) with 1.0 μM ShjDDO.

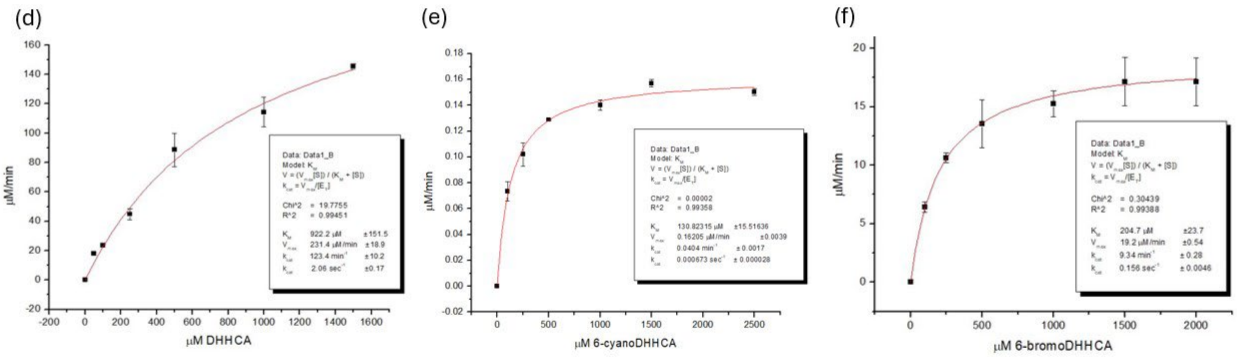

Figure S55. Plots of LmbB1 L-DOPA 2,3-dioxygenase reaction with DHHCA (**3**) and synthetic DHHCA derivatives (i.e. 6- bromoDHHCA (**4**), 6-cyanoDHHCA (**5**)) as a function of substrate concentration in reaction buffer (50 mM HEPES, 10% glycerol, pH 7.50). LmbB1 L-DOPA 2,3-dioxygenase (2.0-4.0 μM) was reacted with excess oxygen and various DHHCA/derivative concentrations (0-5000 μM at 22 °C). The reaction of L-DOPA 2,3-dioxygenase with DHHCA/derivative was followed by the appearance of the steady state cleavage product using UV-Visible spectroscopy. Initial rates were obtained from the slopes of the linear regions of the progress curves and plotted versus substrate concentration to yield a hyperbola, which was fit to the Michaelis-Menten expression: $\upsilon = (V_{max}[S])/(K_M + [S])$ using standard nonlinear regression (Origin 6.0, Microcal) to obtain $k_{cat} = V_{max}/[E_T]$; and the error propagated. (d) vary DHHCA with 2.0 μM LmbB1 at 100% O_2, (e) vary 6-cyanoDHHCA with 4.0 μM LmbB1 at 100% O_2, (f) vary 6-bromoDHHCA with 2.0 μM LmbB1 at 100% O_2.

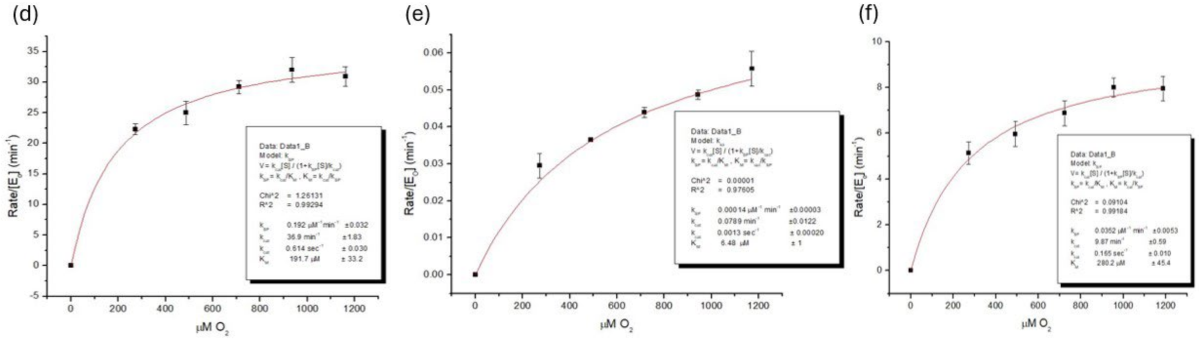

Figure S56. Plots of LmbB1 L-DOPA 2,3-dioxygenase reaction with DHHCA (**3**) and synthetic DHHCA derivatives (i.e. 6- bromoDHHCA (**4**), 6-cyanoDHHCA (**5**)) as a function of oxygen concentration in reaction buffer (50 mM HEPES, 10% glycerol, pH 7.50). LmbB1 L-DOPA 2,3-dioxygenase (2.0-2.5 µM) was reacted with excess DHHCA/derivative and various oxygen concentrations (200-1200 µM at 22°C). The reaction of L-DOPA 2,3-dioxygenase with DHHCA/derivative as a function of oxygen concentration was followed by the appearance of the steady state cleavage product using UV-Visible spectroscopy. Initial rates were obtained from the slopes of the linear regions of the progress curves and plotted versus substrate concentration to yield a hyperbola, which was fit to the following alternative derivation of the Michaelis-Menten expression: $\upsilon = (k_{SP}[S])/(1+k_{SP}[S]/k_{cat})$[1] using standard nonlinear regression (Origin 6.0, Microcal) to obtain k_{SP} (or k_{cat}/K_M) directly; K_M was calculated from the fitted k_{SP} and kcat parameters according to the following expression: $K_M = k_{cat}/k_{SP}$ and the error propagated. (d) 4500 µM DHHCA (5x K_M, DHHCA) with 2.5 µM LmbB1, (e) 2000 µM 6-cyanoDHHCA (10x K_M, 6-cyanoDHHCA) with 2.5 µM LmbB1, (f) 1000 µM 6-bromoDHHCA (5x K_M, 6-bromoDHHCA) with 2.0 µM LmbB1.

Pre-steady State Analysis of ShjDDO with DHHCA and 6-cyanoDHHCA

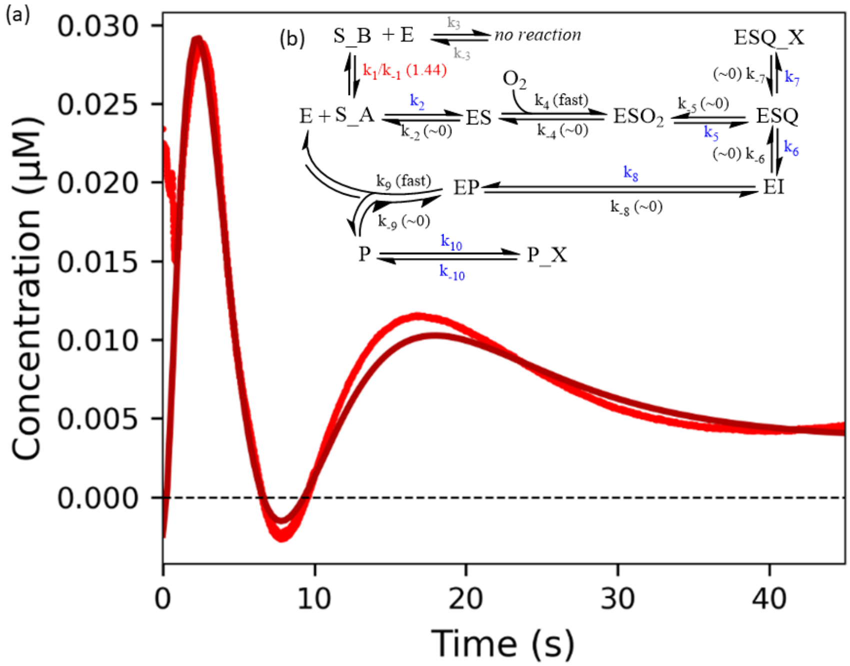

(c) CyanoDHHCA (5.6uM) and *Shj*DDO (56uM) at pH 7.35.

pH 7.35	Rate ± is Fitspace™ range
k_2 (μM^{-1} s^{-1})	0.029 ± 0.0058
k_5 (s^{-1})	0.607 ± 0.12
k_6 (s^{-1})	0.556 ± 0.148
k_7 (s^{-1})	0.2965 ±0.0225
k_8 (s^{-1})	0.2265 ± 0.0435
k_{10} (s^{-1})	0.0412 ± 0.0177
k_{-10} (s^{-1})	0.07525 ± 0.00925
a‡ (M^{-1} $cm^{-}1$)	38,950 ± 9,250
c†	< 0.0031

‡*a is the extinction coefficient on ESQ*
†*c is the y-axis offset which has only an upper limit.*

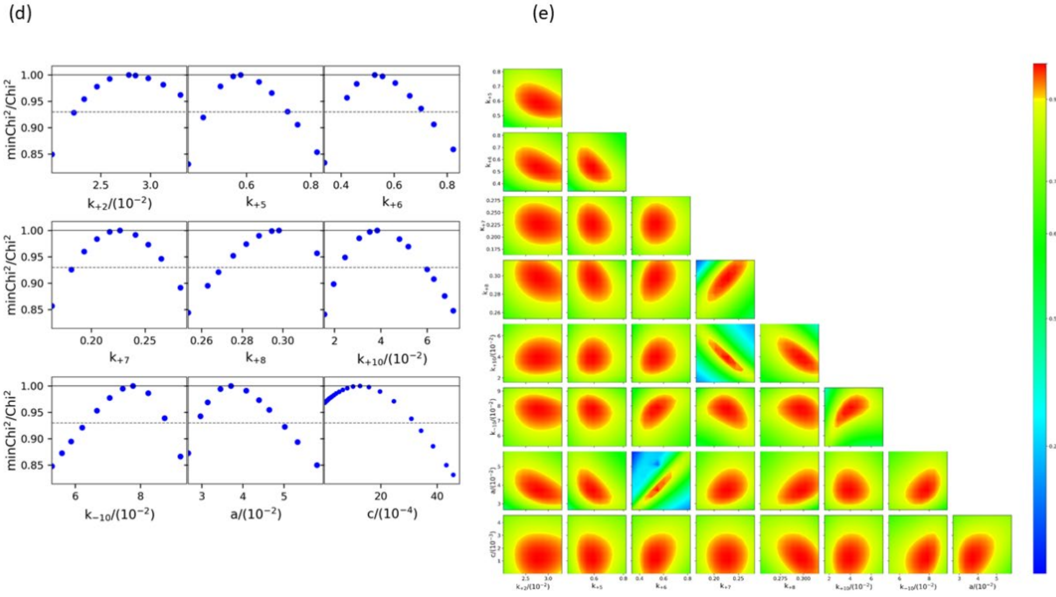

Figure S57. Pre-steady state analysis of the 6-cyanoDHHCA (**5**) reaction with *S. hygroscopicus jingganensis* L-DOPA 2,3-dioxygenase (ShjDDO). **(a)** 6-CyanoDHHCA (5.6µM) ShjDDO (56 µM) at pH 7.35, 14°C, ~1000 µM O_2 and 380 nm produces two dominant spectroscopic features between 2-3 seconds and a slower feature than accumulates between 10-20 seconds consistent with transient accumulation of a semiquinone species followed by the ring cleavage product. The first 800 ms contain additional low intensity spectroscopic features that were excluded from the fit. From 0.8 to 45 seconds, the data (light red) are fit to the model (dark red) shown in (b) and

fitted parameters are shown in (c) with the calculated Fitspace range. **(b)** A reaction pathway consistent with (a) and the data presented herein. Some rates were fixed as either "fast" (i.e. $1x10^9$) or very slow ($1x10^{-12}$, i.e. ~0) as described in the text. The equilibrium represented by k_1 and k_{-1} was fixed at the ratio of 6-cyanoDHHCA charge states as predicted by the experimentally determined pK_a value (Table 1) and the Henderson-Hasselbach equation (i.e. 1.44 at pH 7.35). Extinction coefficients were fitted and/or fixed during fitting as described in the text, and interpreted using the observable expression: a*(ESQ-ESQ_X) + b*(EP+P) – c, where a is the extinction coefficient applied to ESQ, b is the independently experimentally determined extinction coefficient on P, and c is a y-axis offset. E and S_A and S_B are the enzyme and two charge states of the substrate, in this case – 6-cyanoDHHCA – binding prior to O_2; however, the software consistently drove one of the pathways to the ES complex to zero, where it was fixed during FitSpace calculations. ESO_2 is the ternary complex of enzyme, substrate and oxygen. ESQ represents the putative semiquinone intermediate a UV-absorbing species, and ESQ_X is the species formed from the non-productive quenching of the semiquinone. EI represents subsequent intermediates that are UV-inactive. The Fe-alkylperoxo intermediate is predicted to follow the semiquinone, but it and additional intermediates are UV-inactive and therefore rates surrounding their appearance and disappearance are not constrained by these data; therefore, the rate for EI = EP represents all the rates for the appearance and disappearance of UV-inactive species between the semiquinone intermediate and the semialdehyde product (P). EP is the enzyme product complex, and P is the free semialdehyde product. P can then degrade non-enzymatically to the spectroscopically silent P_X. **(c)** Fitted parameters for (a) according to the model in (b), the value shown is the center of the FitSpace range, with the magnitude of the range expressed as ± values from the center. **(d)** Chi^2 plots of the fitted parameters from the model in (b) applied to the data in (a). **(e)** FitSpace confidence contour analysis[2] for (a), based on the model in (b).

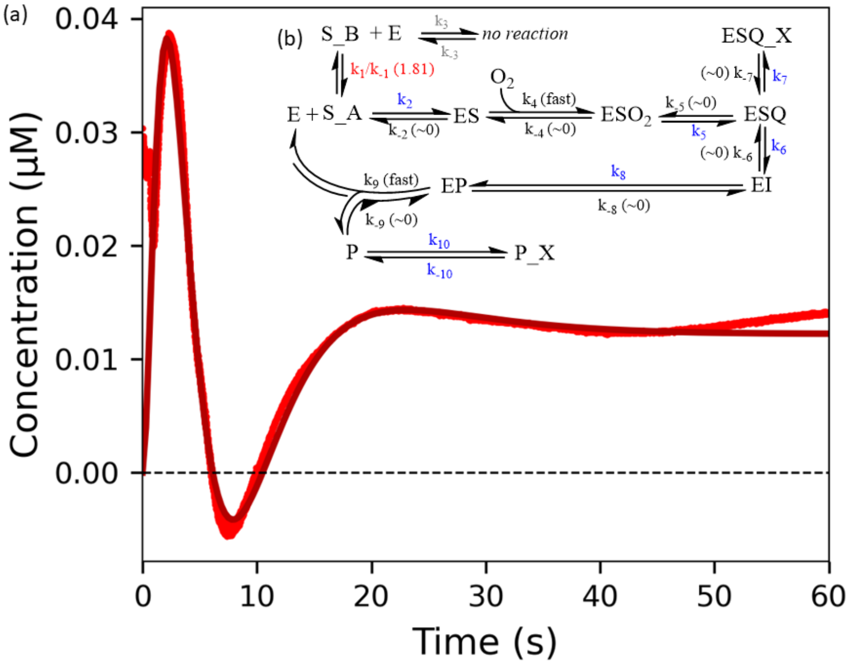

(c) CyanoDHHCA (5.9uM) and *Shj*DDO (59uM) at pH 7.25.

pH 7.25	Rate ± is Fitspace™ range
k_2 (μM^{-1} s^{-1})	0.0336 ± 0.0056
k_5 (s^{-1})	0.6365 ± 0.1225
k_6 (s^{-1})	0.465 ± 0.106
k_7 (s^{-1})	0.29775 ± 0.02575
k_8 (s^{-1})	0.25 ± 0.058
k_{10} (s^{-1})	0.02359 ± 0.01931
k_{-10} (s^{-1})	0.0822 ± 0.0328
a‡ (M^{-1} cm^{-1})	40,300 ± 810
c†	*0*

‡*a is the extinction coefficient on ESQ*

†*c is the y-axis offset which was fixed at zero. If c is not fixed, the parameters still constrain, but the ranges are wider – see panels (f) and (g)*

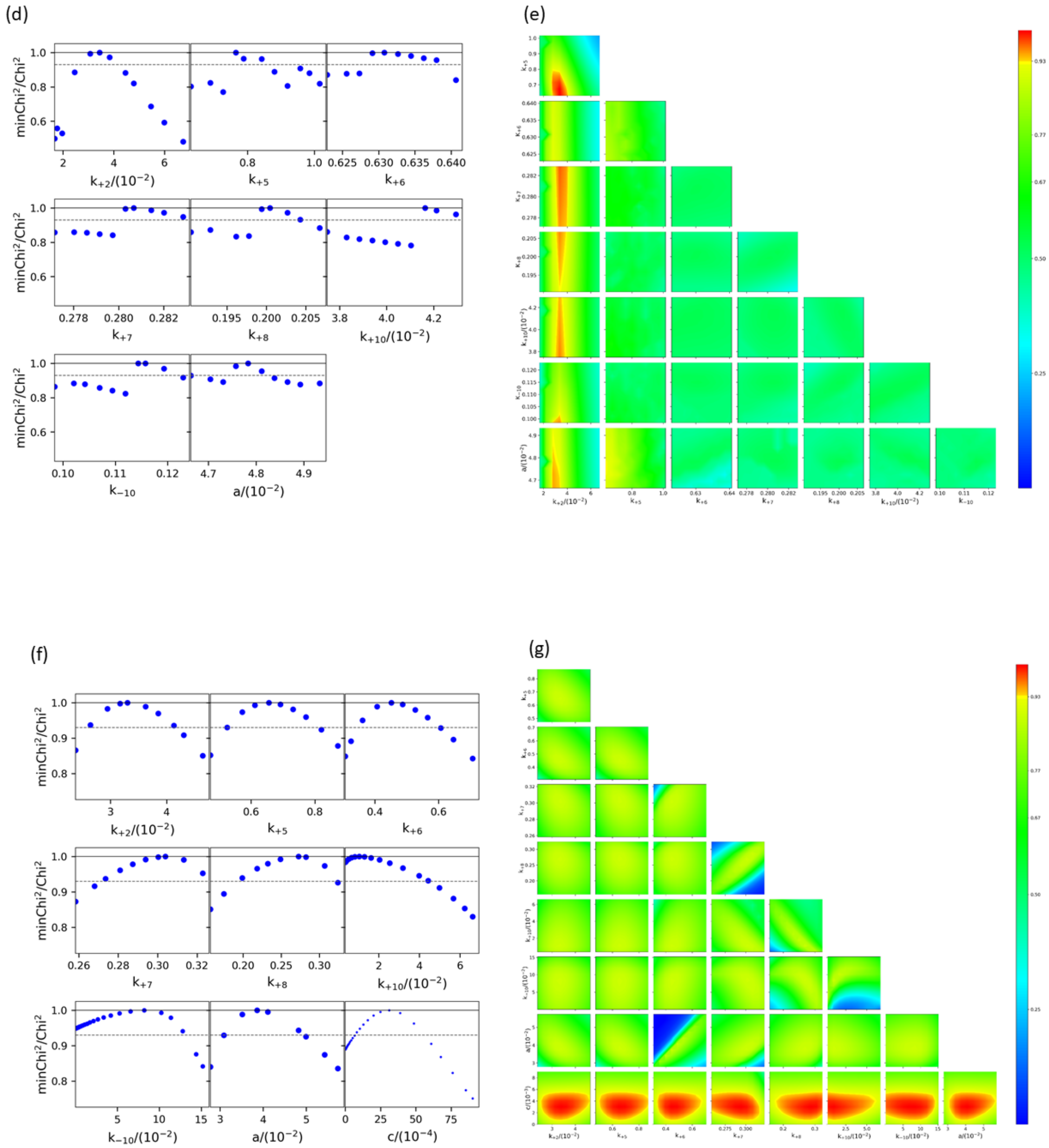

Figure S58. Pre-steady state analysis of the 6-cyanoDHHCA (**5**) reaction with *S. hygroscopicus jingganensis* L-DOPA 2,3-dioxygenase (ShjDDO). (a) 6-CyanoDHHCA (5.6 µM) ShjDDO (56 µM) at pH 7.25, 14°C, ~1000 µM O_2 and 380 nm produces two dominant spectroscopic features between 2-3 seconds and a slower feature than accumulates between 10-20 seconds consistent with transient accumulation of a semiquinone species followed by the ring cleavage product. The first 800 ms contain additional low intensity spectroscopic features that were excluded from the fit. From 0.8 to 45 seconds, the data (light red) are fit to the model (dark red) shown in (b) and

fitted parameters are shown in (c) with the calculated Fitspace range. **(b)** A reaction pathway consistent with (a) and the data presented herein. Some rates were fixed as either "fast" (i.e. $1x10^9$) or very slow ($1x10^{-12}$, i.e. ~0) as described in the text. The equilibrium represented by k_1 and k_{-1} was fixed at the ratio of 6-cyanoDHHCA charge states as predicted by the experimentally determined pK_a value (Table 1) and the Henderson-Hasselbach equation. Extinction coefficients were fitted and/or fixed during fitting as described in the text, and the data interpreted using the observable expression: a*(ESQ-ESQ_X) + b*(EP+P) – c, where a is the extinction coefficient applied to ESQ, b is the independently experimentally determined extinction coefficient for P, and c is a y-axis offset, which was fixed at zero. If c is not fixed, the parameters still constrain, but the ranges are wider – see panels (f) and (g). E and S_A and S_B are the enzyme and two charge states of the substrate, in this case – 6-cyanoDHHCA – binding prior to O_2; however, the software consistently drove one of the pathways to the ES complex to zero, where it was fixed during FitSpace calculations. ESO_2 is the ternary complex of enzyme, substrate and oxygen. ESQ represents the putative semiquinone intermediate a UV-absorbing species, and ESQ_X is the species formed from the non-productive quenching of the semiquinone. EI represents subsequent intermediates that are UV-inactive. The Fe-alkylperoxo intermediate is predicted to follow the semiquinone, but it and additional intermediates are UV-inactive and therefore rates surrounding their appearance and disappearance are unaccounted not constrained by these data; therefore, the rate for EI = EP represents all the rates for the appearance and disappearance of UV-inactive species between the semiquinone intermediate and the semialdehyde product (P). EP is the enzyme product complex, and P is the free semialdehyde product. P can then degrade non-enzymatically to the spectroscopically silent P_X. **(c)** Fitted parameters for (a) according to the model in (b), the value shown is the center of the FitSpace range, with the magnitude of the range expressed as ± values from the center. **(d)** Chi^2 plots of the fitted parameters from the model in (b) applied to the data in (a). **(e)** FitSpace confidence contour analysis[2] for (a), based on the model in (b).

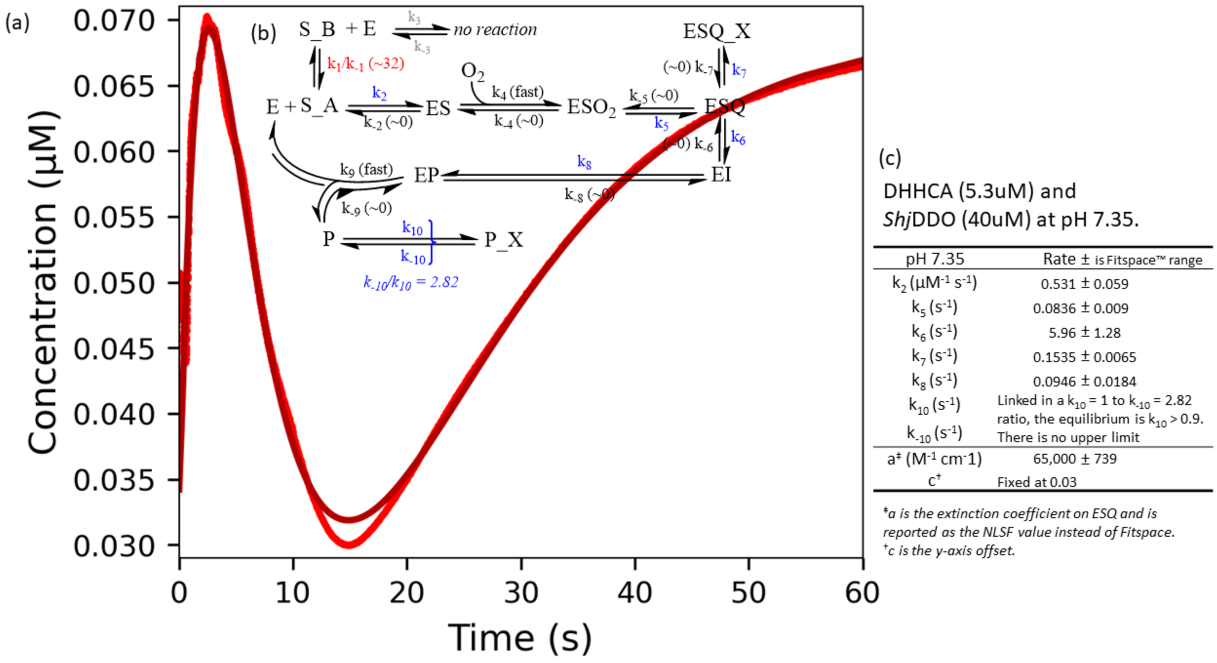

(c)

DHHCA (5.3uM) and *Shj*DDO (40uM) at pH 7.35.

pH 7.35	Rate ± is Fitspace™ range
k_2 (μM^{-1} s^{-1})	0.531 ± 0.059
k_5 (s^{-1})	0.0836 ± 0.009
k_6 (s^{-1})	5.96 ± 1.28
k_7 (s^{-1})	0.1535 ± 0.0065
k_8 (s^{-1})	0.0946 ± 0.0184
k_{10} (s^{-1})	Linked in a k_{10} = 1 to k_{-10} = 2.82 ratio, the equilibrium is k_{10} > 0.9.
k_{-10} (s^{-1})	There is no upper limit
$a^{‡}$ (M^{-1} cm-1)	65,000 ± 739
$c^{†}$	Fixed at 0.03

‡a is the extinction coefficient on ESQ and is reported as the NLSF value instead of Fitspace.
†c is the y-axis offset.

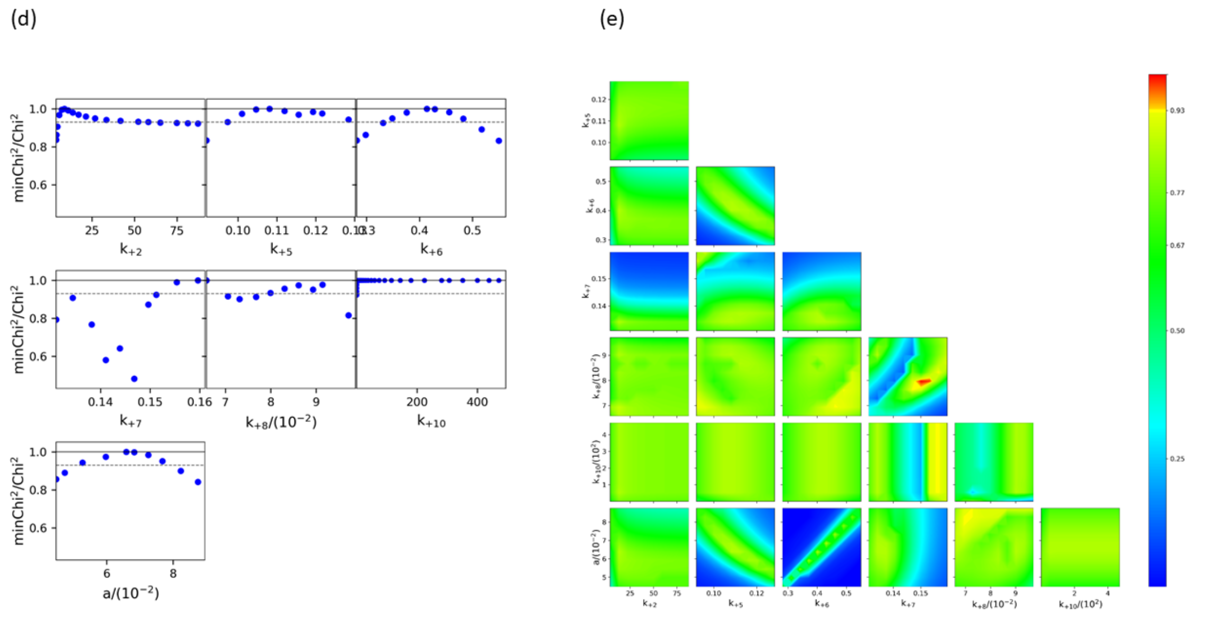

Figure S59. Pre-steady state analysis of the DHHCA (**3**) reaction with *S. hygroscopicus jingganensis* L-DOPA 2,3-dioxygenase (ShjDDO). (a) DHHCA (5.3µM) ShjDDO (40 µM) at pH 7.35, 14°C, ~1000 µM O_2 and 380 nm produces two dominant spectroscopic features between 2-3 seconds and a slower feature than accumulates between 10-20 seconds consistent with transient accumulation of a semiquinone species followed by the ring cleavage product. The first 550 ms contain additional low intensity spectroscopic features that were excluded from the fit. From 0.55 to 60 seconds, the data (light red) are fit to the model (dark red) shown in (b) and

fitted parameters are shown in (c) with the calculated Fitspace range. **(b)** A reaction pathway consistent with (a) and the data presented herein. Some rates were fixed as either “fast” (i.e. $1x10^9$) or very slow ($1x10^{-12}$, i.e. ~0) as described in the text. The equilibrium represented by k_1 and k_{-1} was fixed at the ratio of DHHCA charge states as predicted by the experimentally determined pK_a value (Table 1) and the Henderson-Hasselbach equation. Extinction coefficients were fitted and/or fixed during fitting as described in the text, and the data interpreted using the observable expression: a*(ESQ-ESQ_X) + b*(EP+P) – c, where a is the extinction coefficient applied to ESQ, b is the independently experimentally determined extinction coefficient for P, and c is a y-axis offset, which was fixed at a value consistent with the data. E and S_A and S_B are the enzyme and two charge states of the substrate, in this case DHHCA, binding prior to O_2; however, the software consistently drove one of the pathways to the ES complex to zero, where it was fixed during FitSpace calculations. ESO_2 is the ternary complex of enzyme, substrate and oxygen. ESQ represents the putative semiquinone intermediate a UV-absorbing species, and ESQ_X is the species formed from the non-productive quenching of the semiquinone. EI represents subsequent intermediates that are UV-inactive. The Fe-alkylperoxo intermediate is predicted to follow the semiquinone, but it and additional intermediates are UV-inactive and therefore rates surrounding their appearance and disappearance are unaccounted not constrained by these data; therefore, the rate for EI = EP represents all the rates for the appearance and disappearance of UV-inactive species between the semiquinone intermediate and the semialdehyde product (P). EP is the enzyme product complex, and P is the free semialdehyde product. P can then degrade non-enzymatically to the spectroscopically silent P_X. **(c)** Fitted parameters for (a) according to the model in (b), the value shown is the center of the FitSpace range, with the magnitude of the range expressed as ± values from the center. **(d)** Chi^2 plots of the fitted parameters from the model in (b) applied to the data in (a). (e) FitSpace confidence contour analysis[2] for (a), based on the model in (b).

Small molecule docking

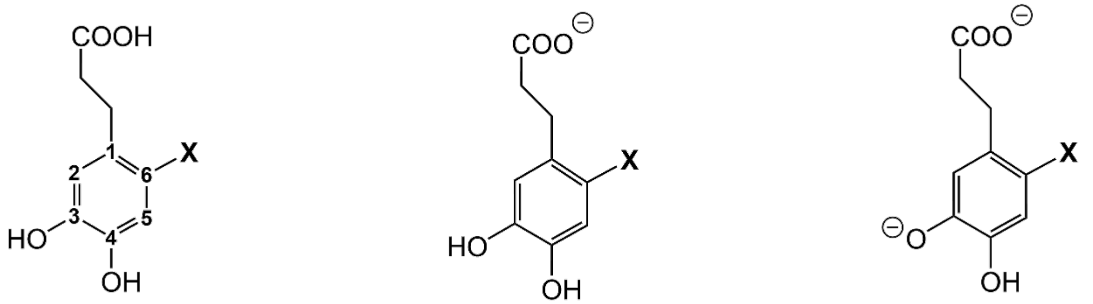

6-X-3,4-dihydroxy hydrocinnamic acid (DHHCA) *charge state (0)*

6-X-3,4-dihydroxy hydrocinnamic acid (DHHCA) *charge state (-1)*

6-X-3,4-dihydroxy hydrocinnamic acid (DHHCA) *charge state (-2)*

Figure S60. Possible 6-X/DHHCA charge states using in docking.

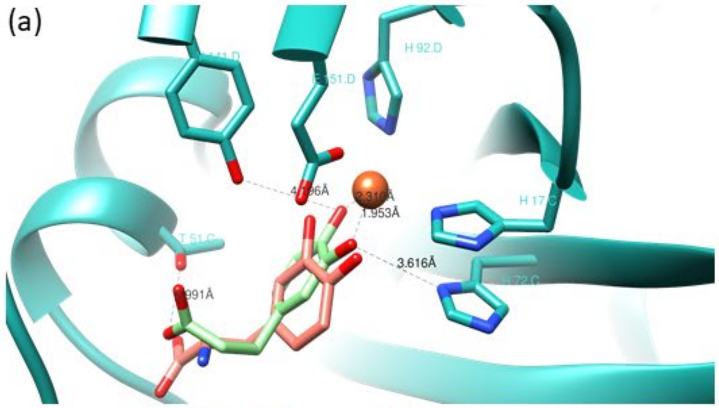

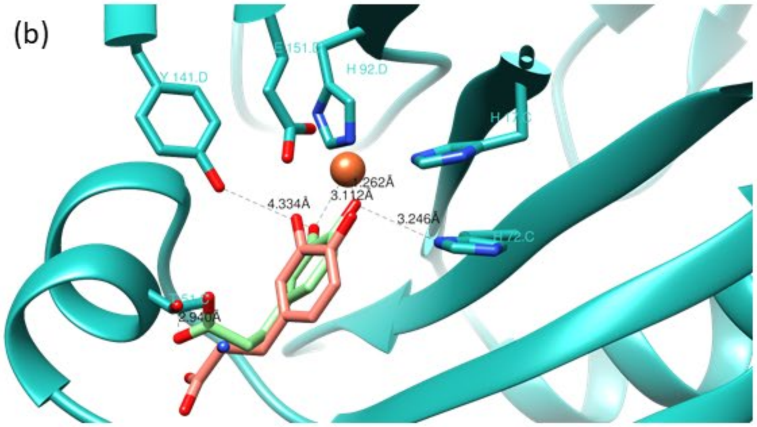

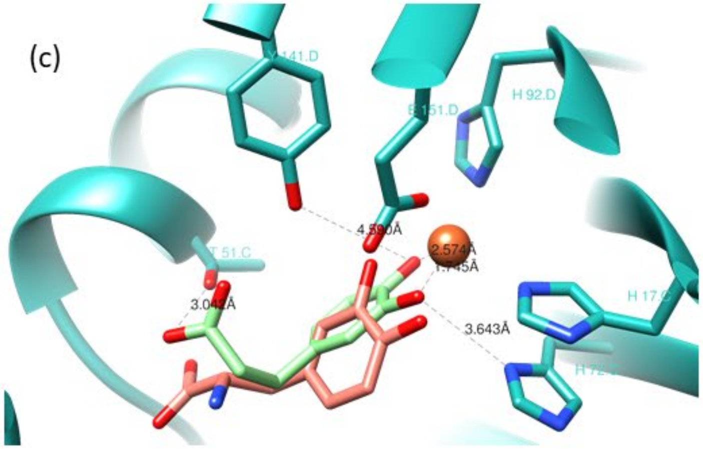

	Charge	#0 Fe2 201.A FE - 1 O (Å)	#0 Fe2 201.A FE - 1 O (Å)	1 O - #0 THR 51.C OG1 (Å)	1 O - #0 HIS 72.C ND1 (Å)	#3.6 1 O - #0 TYR 141.D OH (Å)
(a)	0	2.310	1.953	2.991	3.616	4.196
(b)	-1	3.112	1.262	2.940	3.246	4.334
(c)	-2	2.574	1.745	3.042	3.643	4.590

Figure S61. Three DHHCA (**3**) charge states (green) docked into a model of ShjDDO (aqua). Measurements to key active site positions (i.e. active site Fe^{II}, the side chain oxygen of Thr51, the ring nitrogen of His72, and the phenolic oxygen of Tyr141 are in angstroms. L-DOPA from 6ON3/SsDDO is overlayed in pink. (a) DHCHA charge state 0, (b) DHHCA charge state -1, (c) DHHCA charge state -2.

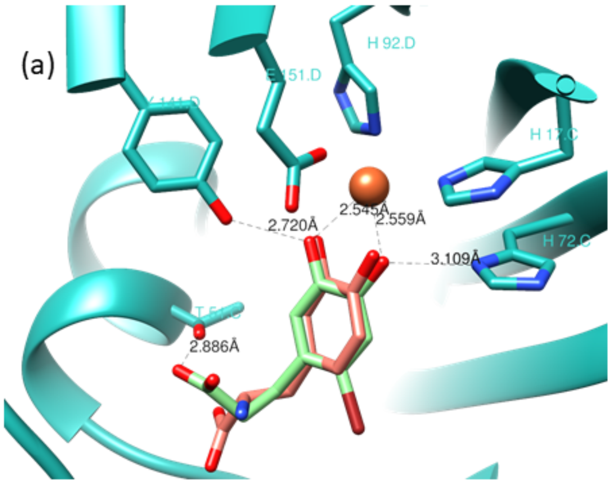

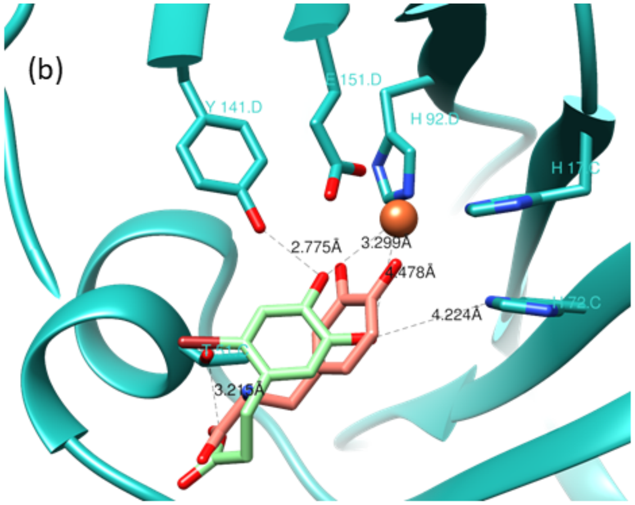

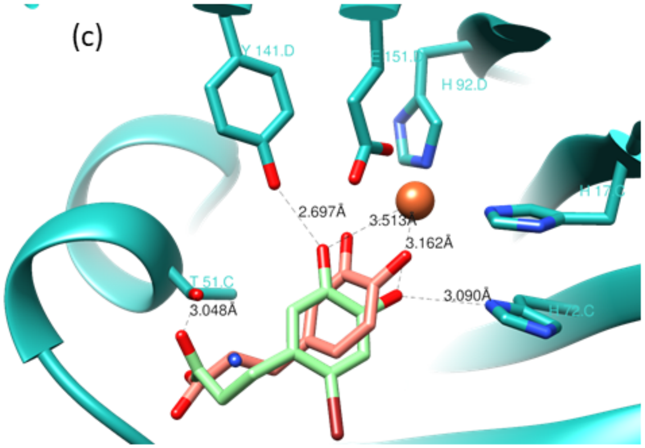

	Charge	#0 Fe2 201.A FE - O (Å)	#0 Fe2 1 201.A FE - 1 O (Å)	1 O - #0 THR 51.C OG1 (Å)	1 O - #0 HIS 72.C ND1 (Å)	#3.6 1 O - #0 TYR 141.D OH (Å)
(a)	0	2.559	2.545	2.886	3.109	2.720
(b)	-1	4.478	3.299	3.215	4.224	2.775
(c)	-2	3.513	3.162	3.048	3.090	2.697

Figure S62. Three 6-bromoDHHCA (**4**) charge states (green) docked into a model of ShjDDO (aqua). Measurements to key active site positions (i.e. active site Fe^{II}, the side chain oxygen of Thr51, the ring nitrogen of His72, and the phenolic oxygen of Tyr141 are in angstroms. L-DOPA from 6ON3/SsDDO is overlayed in pink. (a) 6-bromoDHCHA Charge state 0, (b) 6-bromoDHCHA charge state -1, (c) 6-bromoDHCHA charge state -2.

(a)

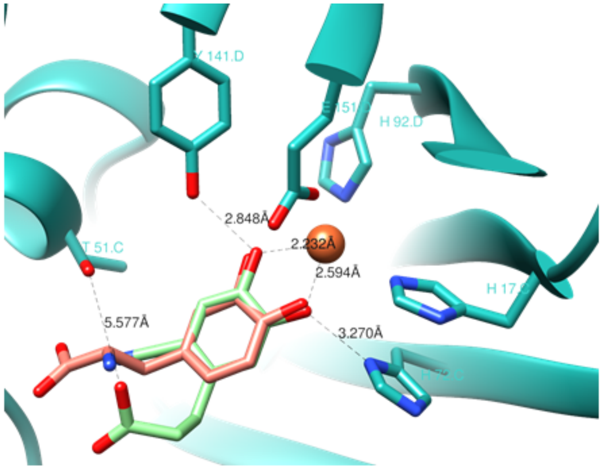

(b)

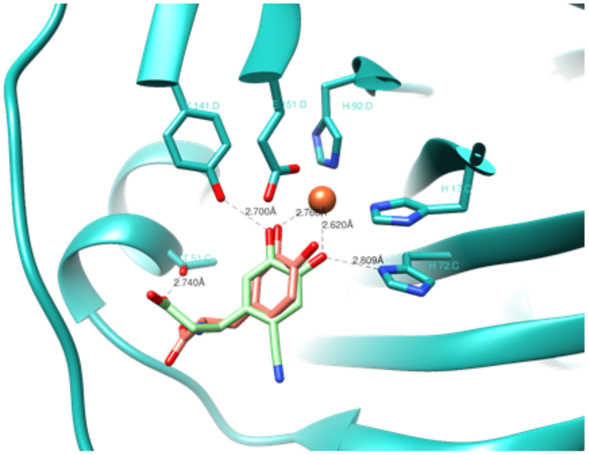

(c)

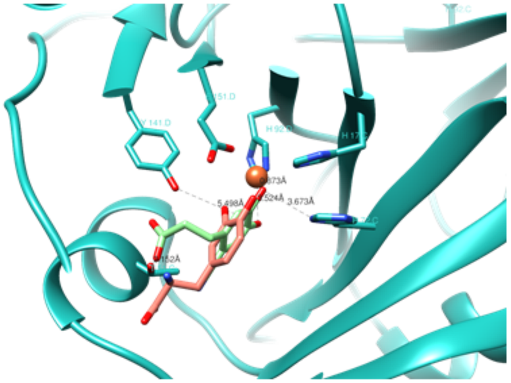

	Charge	#0 Fe2 201.A FE - 1 O (Å)	#0 Fe2 201.A FE - 1 O (Å)	1 O - #0 THR 51.C OG1 (Å)	1 O - #0 HIS 72.C ND1 (Å)	#3.6 1 O - #0 TYR 141.D OH (Å)
(a)	0	2.232	2.594	5.577	3.270	2.848
(b)	-1	2.760	2.620	2.740	2.809	2.700
(c)	-2	3.524	0.873	3.152	3.673	5.498

Figure S63. Three 6-cyanoDHHCA (**5**) charge states (green) docked into a model of ShjDDO (aqua). Measurements to key active site positions (i.e. active site Fe2+, Thr51, His72, Tyr141 are in angstroms. L-DOPA from 6ON3/SsDDO is overlayed in pink. (a) 6-cyanoDHCHA charge state 0, (b) 6-cyanoDHHCA charge state -1, (c) 6-cyanoDHHCA charge state -2.

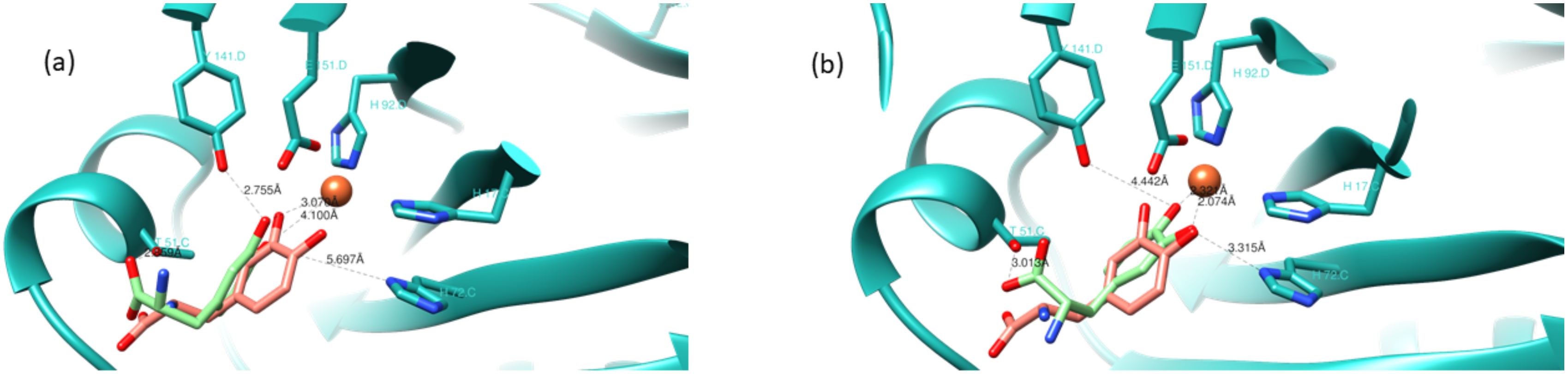

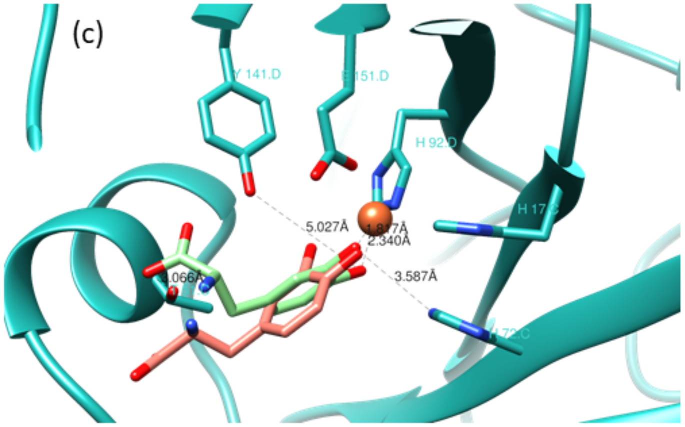

	Charge	#0 Fe2 201.A FE - O (Å)	#0 Fe2 201.A FE - 1 O (Å)	1 O - #0 THR 51.C OG1 (Å)	1 O - #0 HIS 72.C ND1 (Å)	#3.6 1 O - #0 TYR 141.D OH (Å)
(a)	0	4.100	3.070	2.859	5.697	2.755
(b)	-1	2.321	2.074	3.013	3.315	4.442
(c)	-2	2.340	1.817	3.066	3.587	5.027

Figure S64. Three L-DOPA (**1**) charge states (green) docked into a model of ShjDDO (aqua). Measurements to key active site positions (i.e. active site Fe2+, Thr51, His72, Tyr141 are in angstroms. L-DOPA from 6ON3/SsDDO is overlayed in pink. (a) L-DOPA charge state 0, (b) L-DOPA charge state -1, (c) L-DOPA charge state -2.

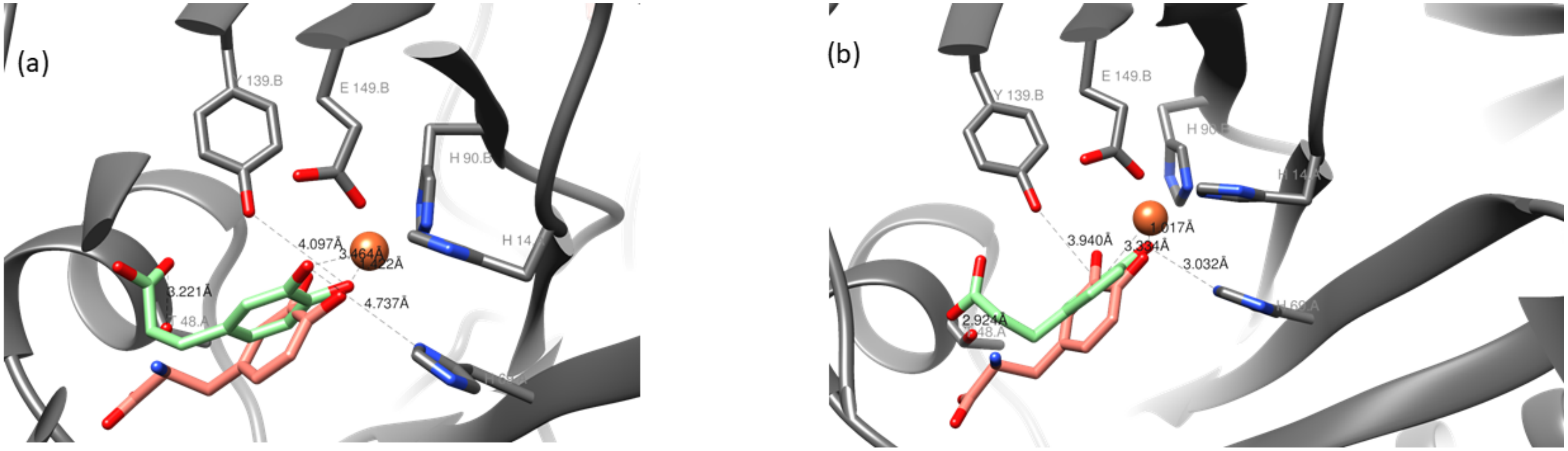

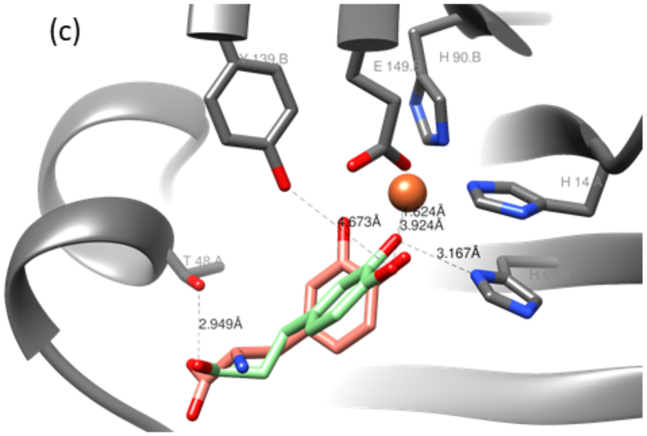

	Charge	#0 Fe2 201.A FE - O (Å)	#0 Fe2 1 201.A FE - 1 O (Å)	1 O - #0 THR 51.C OG1 (Å)	1 O - #0 HIS 72.C ND1 (Å)	#3.6 1 O - #0 TYR 141.D OH (Å)
(a)	0	1.422	3.464	3.221	4.737	4.097
(b)	-1	1.017	3.334	2.924	3.032	3.940
(c)	-2	1.624	3.924	2.949	3.167	4.673

Figure S65. Three DHHCA (**3**) charge states (green) docked into a model of LmbB1 (grey). Measurements to key active site positions (i.e. active site Fe2+, Thr51, His72, Tyr141 are in angstroms. L-DOPA from 6ON3/SsDDO is overlayed in pink. (a) DHHCA charge state 0, (b) DHHCA charge state -1, (c) DHHCA charge state -2

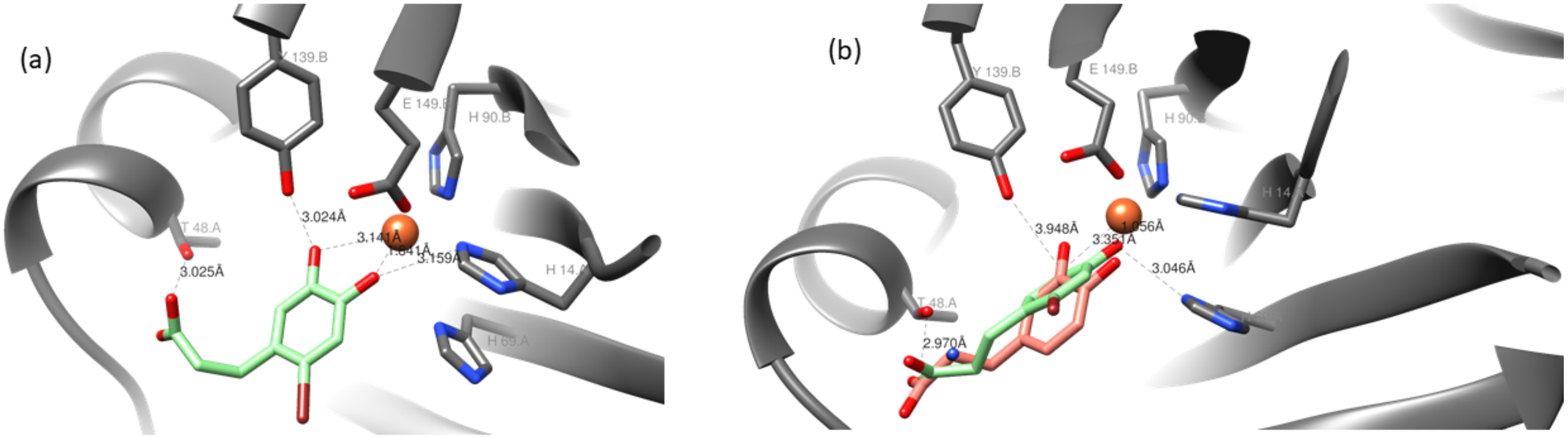

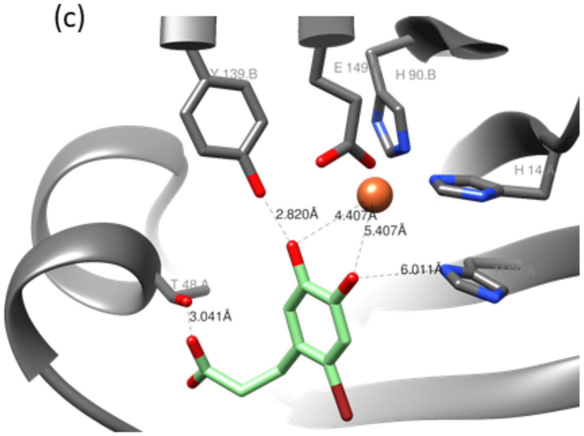

	Charge	#0 Fe2 201.A FE - 1 O (Å)	#0 Fe2 201.A FE - 1 O (Å)	1 O - #0 THR 51.C OG1 (Å)	1 O - #0 HIS 72.C ND1 (Å)	#3.6 1 O - #0 TYR 141.D OH (Å)
(a)	0	1.841	3.141	3.025	3.159	3.024
(b)	-1	1.056	3.351	2.970	3.046	3.948
(c)	-2	4.407	5.407	3.041	6.011	2.820

Figure S66. Three 6-bromoDHHCA **(4)** charge states (green) docked into a model of LmbB1 (grey). Measurements to key active site positions (i.e. active site Fe^{2+}, Thr51, His72, Tyr141 are in angstroms. L-DOPA from 6ON3/SsDDO is overlayed in pink. (a) 6-bromoDHHCA charge state 0, (b) 6-bromoDHHCA charge state -1, (c) 6-bromoDHHCA charge state -2

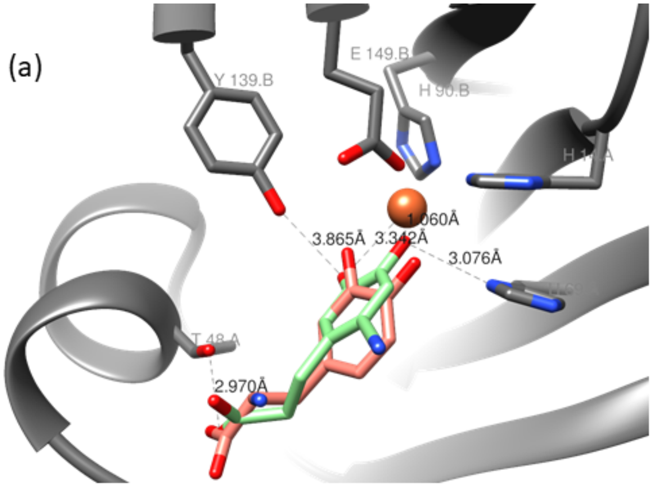

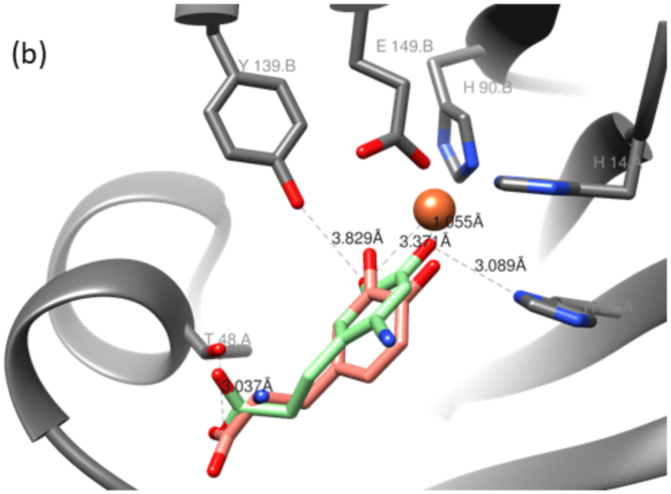

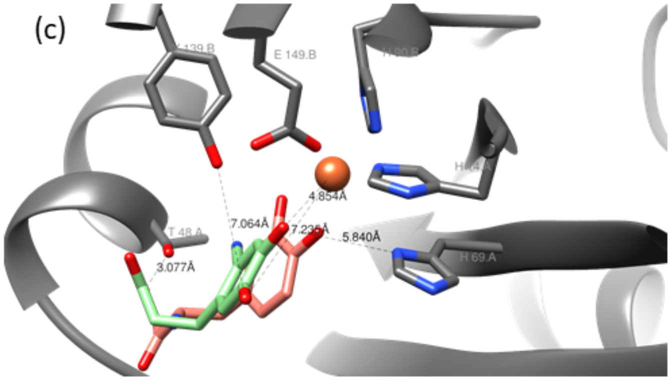

	Charge	#0 Fe2 201.A FE - O (Å)	#0 Fe2 201.A FE - 1 O (Å)	1 O - #0 THR 51.C OG1 (Å)	1 O - #0 HIS 72.C ND1 (Å)	#3.6 1 O - #0 TYR 141.D OH (Å)
(a)	0	3.342	1.060	2.970	3.076	3.865
(b)	-1	3.371	1.055	3.037	3.089	3.829
(c)	-2	4.854	7.235	3.077	5.840	7.064

Figure S67. Three 6-cyanoDHHCA (**5**) charge states (green) docked into a model of LmbB1 (grey). Measurements to key active site positions (i.e. active site Fe2+, Thr51, His72, Tyr141 are in angstroms. L-DOPA from 6ON3/SsDDO is overlayed in pink. (a) 6-cyanoDHHCA charge state 0, (b) 6-cyanoDHHCA charge state -1, (c) 6-cyanoDHHCA charge state -2

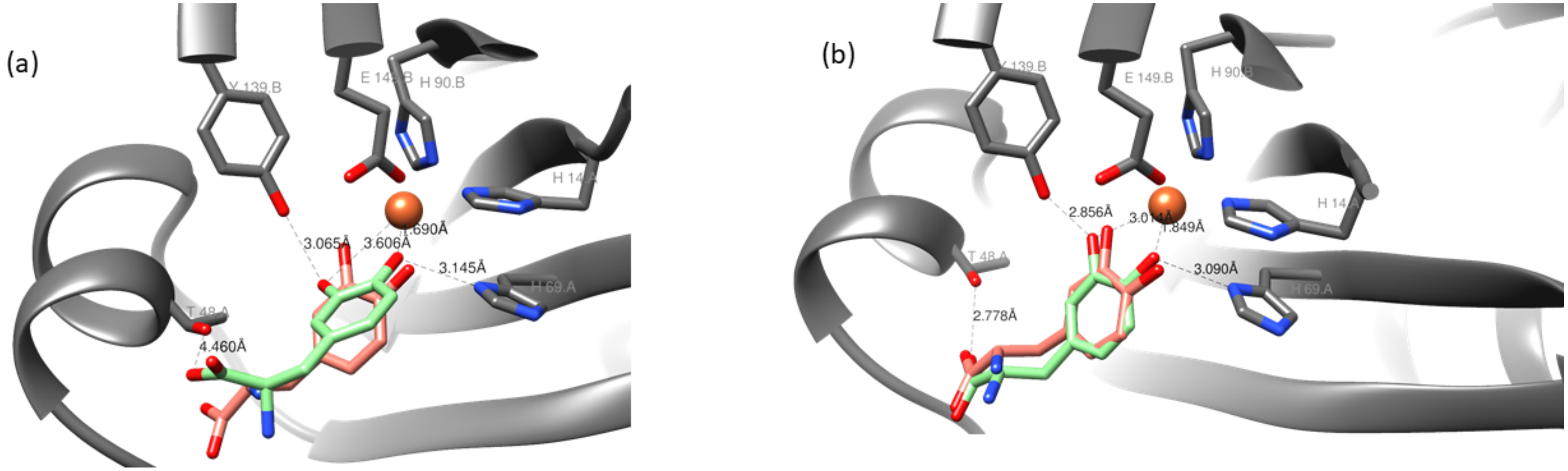

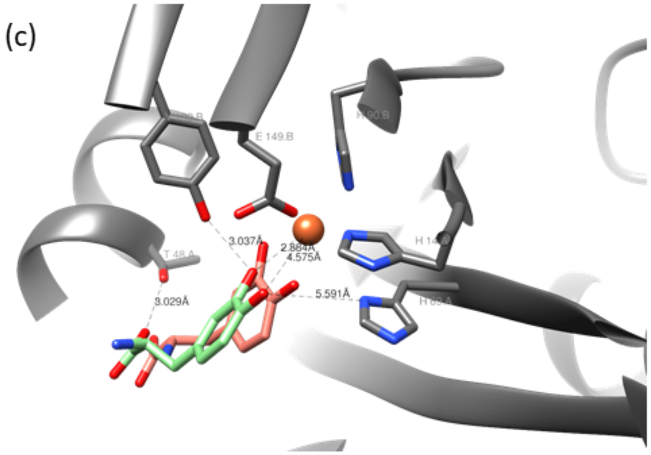

	Charge	#0 Fe2 201.A FE - 1 O (Å)	#0 Fe2 201.A FE - 1 O (Å)	1 O - #0 THR 51.C OG1 (Å)	1 O - #0 HIS 72.C ND1 (Å)	#3.6 1 O - #0 TYR 141.D OH (Å)
(a)	0	3.606	1.690	4.460	3.145	3.065
(b)	-1	3.014	1.849	2.778	3.090	2.856
(c)	-2	2.884	4.575	3.029	5.591	3.037

Figure S68. Three L-DOPA (**1**) charge states (green) docked into a model of LmbB1 (grey). Measurements to key active site positions (i.e. active site Fe^{2+}, Thr51, His72, Tyr141 are in angstroms. L-DOPA from 6ON3/SsDDO is overlayed in pink. (a) L-DOPA charge state 0, (b) L-DOPA charge state -1, (c) L-DOPA charge state -2

Redox potential and pK_a values for catecholic substrates

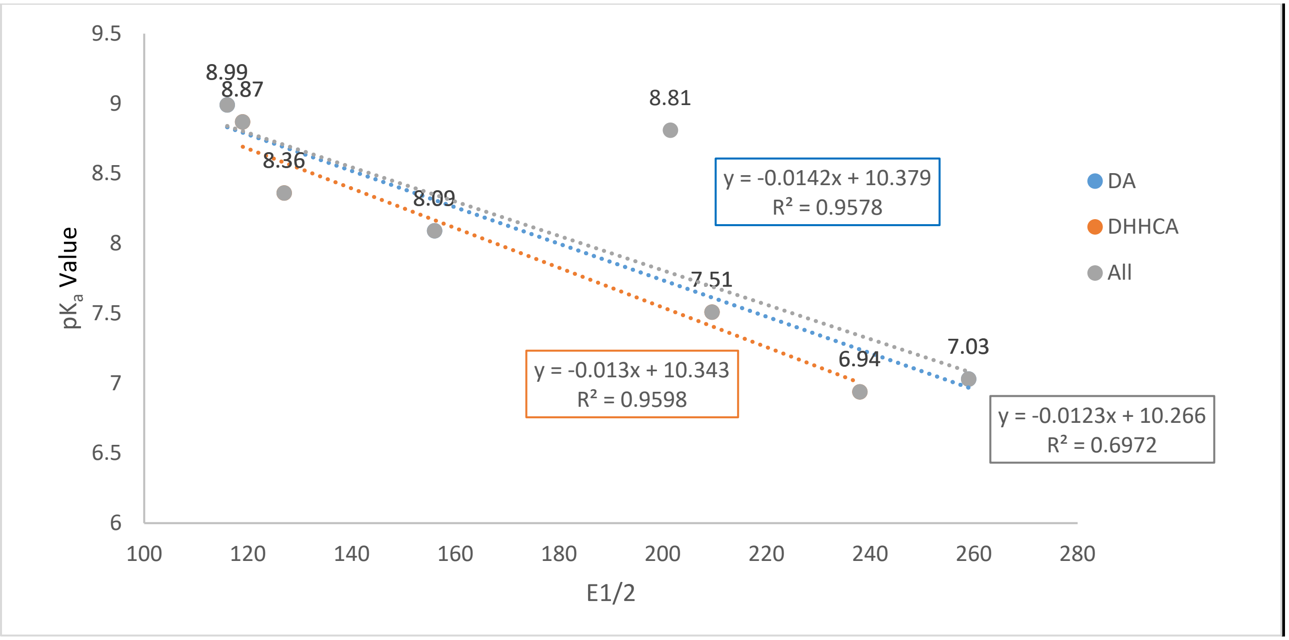

Figure S69. Experimental $E_{1/2}$ at pH 7.4 for each 6-X/dopamine, 6-X/DHHCA and L-DOPA plotted against the experimentally determined pK_a value for each. It was necessary to estimate the E_{pc} for L-DOPA (**1**) and 6-nitroDHHCA (**6**) because the reduction is not readily apparent within the CV (Figures S36 and S46).

References

(1) Johnson, K. A. New Standards for Collecting and Fitting Steady State Kinetic Data. *Beilstein J. Org. Chem.* **2019**, *15* (1), 16–29. https://doi.org/10.3762/bjoc.15.2.

(2) Johnson, K. A.; Simpson, Z. B.; Blom, T. FitSpace Explorer: An Algorithm to Evaluate Multidimensional Parameter Space in Fitting Kinetic Data. *Anal Biochem* **2009**, *387* (1), 30-41. https://doi.org/10.1016/j.ab.2008.12.025.
